# Supplementary figures and images for: Targeted protein degradation systems to enhance Wnt signaling (part 1 of 2)
Source: eLife. 2024 Jun 7;13:RP93908. doi: 10.7554/eLife.93908 (PMC11161174; doi:10.7554/eLife.93908)

# 4F3-RSPO2RA

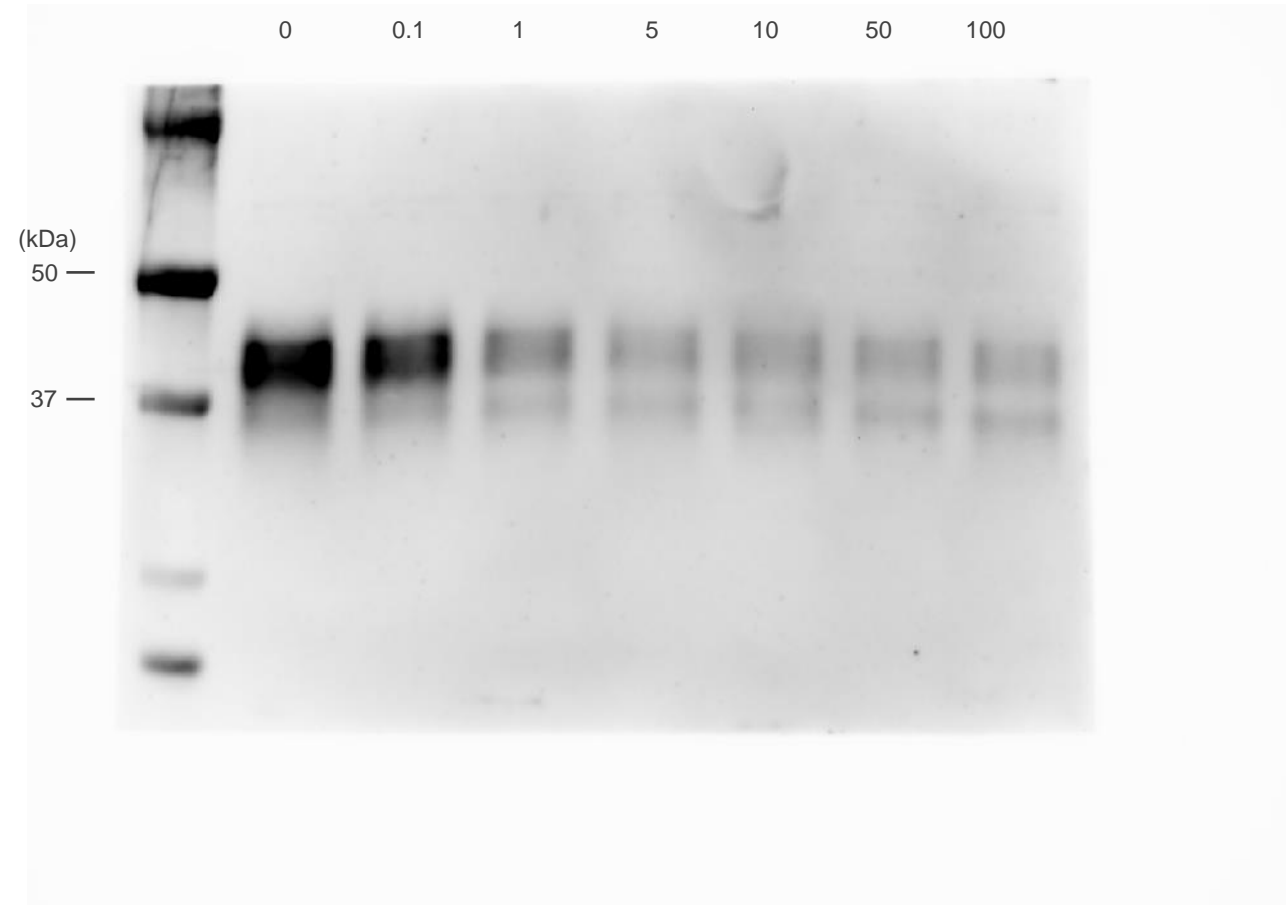

Supplement: Figure 6—source data 1. [file elife-93908-fig6-data1.zip › Figure 6A anti-ASGR1 with 4F3-RSPO2RA treatment Labelled Raw Data.pdf]

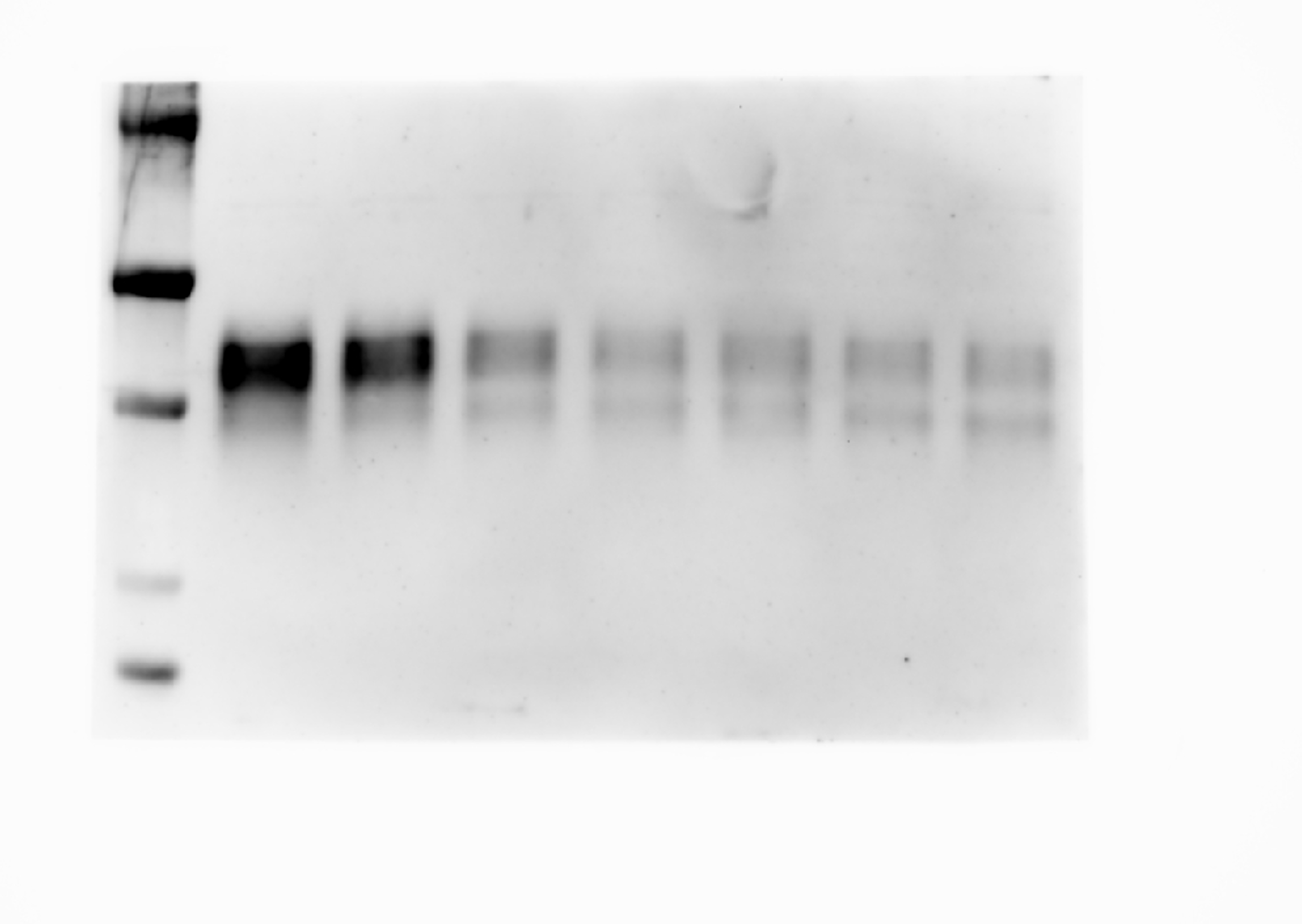

Supplement: Figure 6—source data 1. [file elife-93908-fig6-data1.zip › Figure 6A anti-ASGR1 with 4F3-RSPO2RA treatment Raw Data.tif]

**8G8-RSPO2RA**

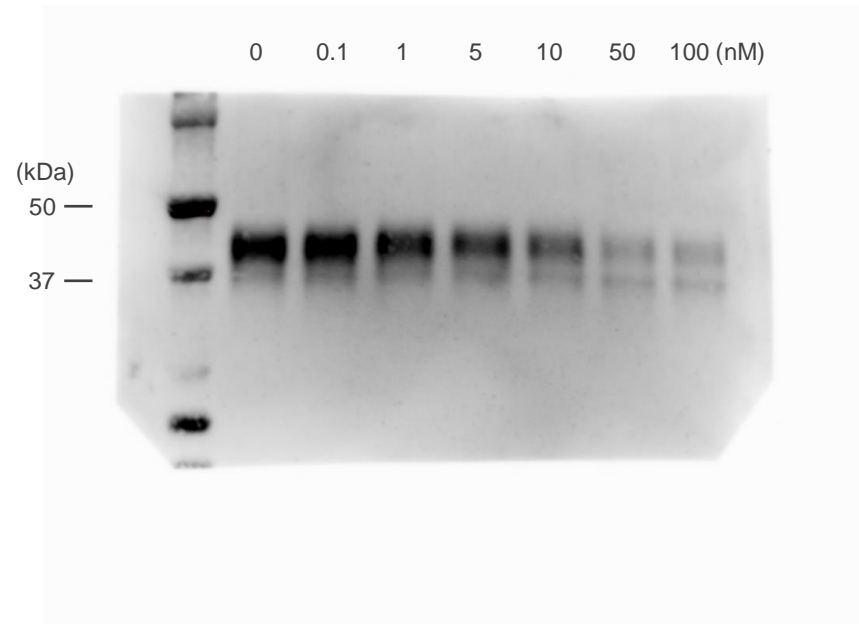

Supplement: Figure 6—source data 1. [file elife-93908-fig6-data1.zip › Figure 6A anti-ASGR1 with 8G8-RSPO2RA treatment Labelled Raw Data.pdf]

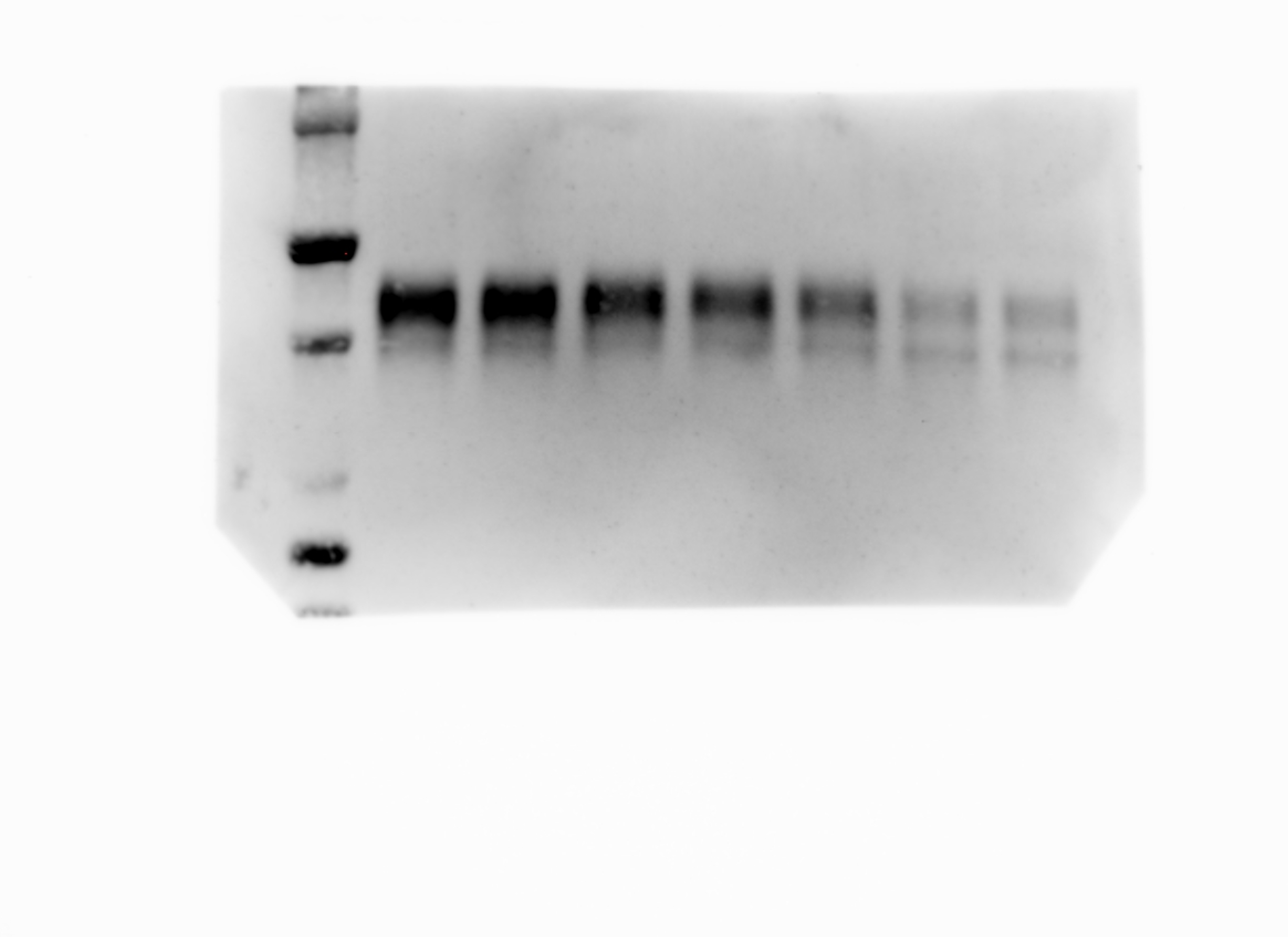

Supplement: Figure 6—source data 1. [file elife-93908-fig6-data1.zip › Figure 6A anti-ASGR1 with 8G8-RSPO2RA treatment Raw Data.tif]

8M24-RSPO2RA

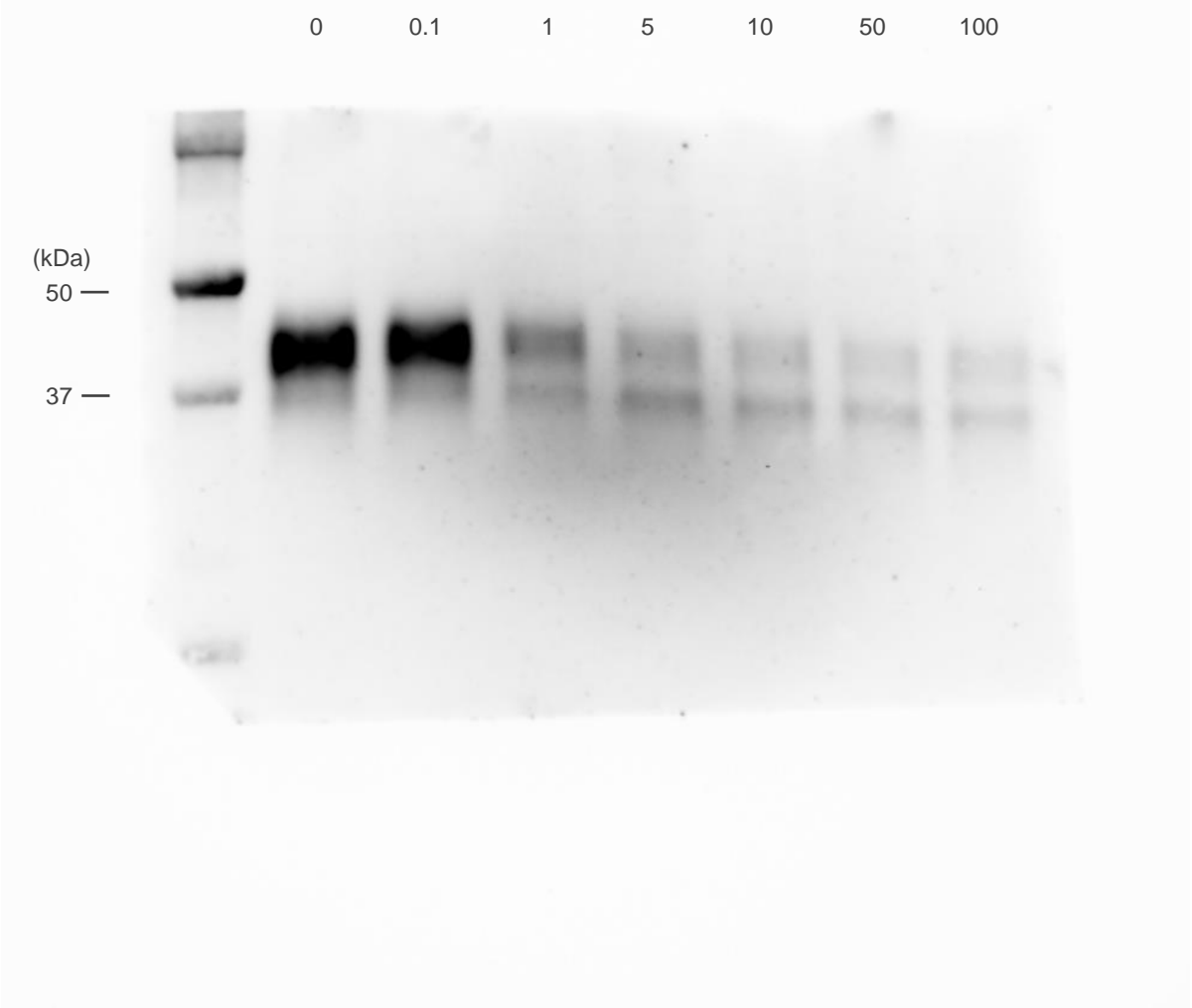

Supplement: Figure 6—source data 1. [file elife-93908-fig6-data1.zip › Figure 6A anti-ASGR1 with 8M24-RSPO2RA treatment Labelled Raw Data.pdf]

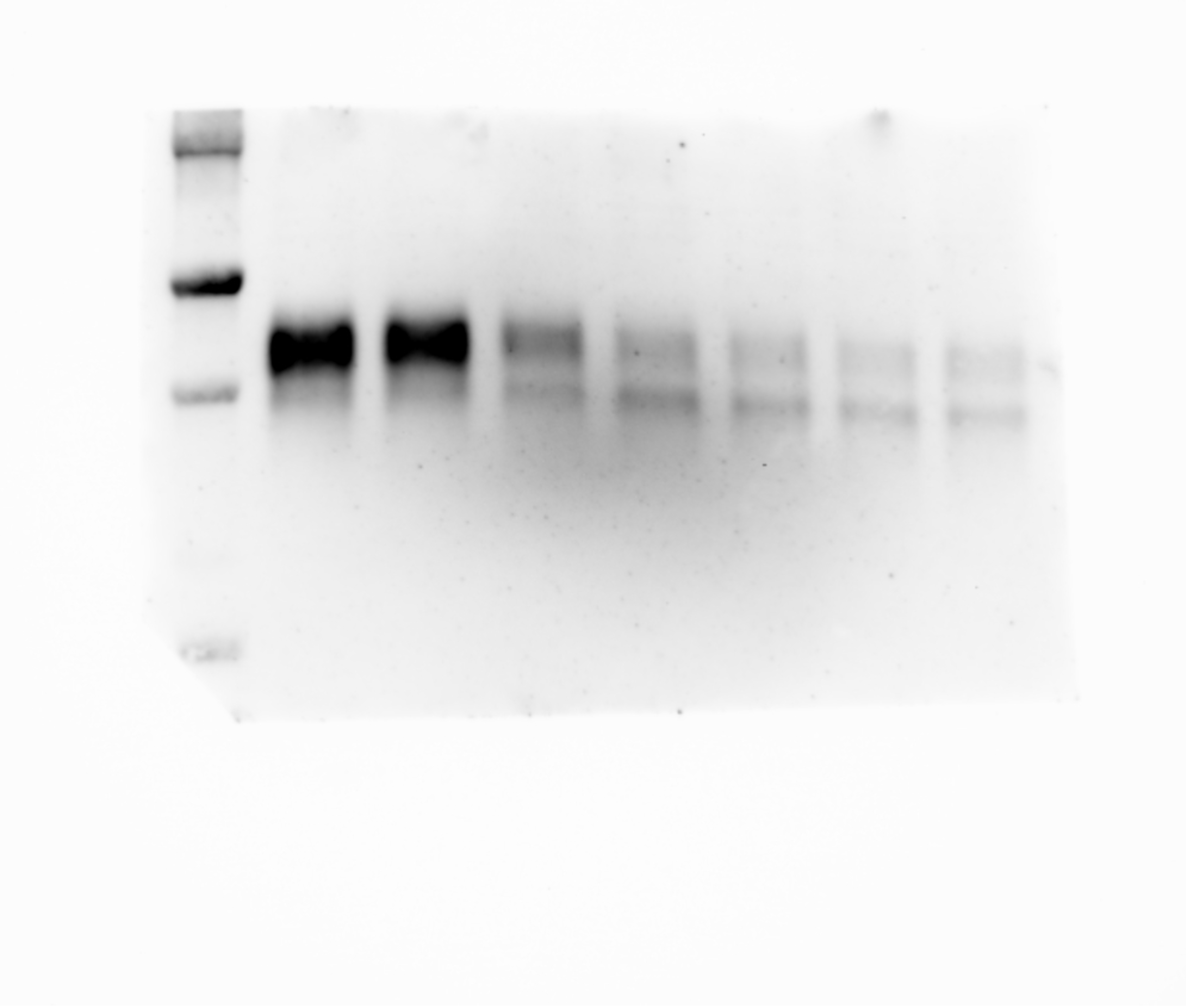

Supplement: Figure 6—source data 1. [file elife-93908-fig6-data1.zip › Figure 6A anti-ASGR1 with 8M24-RSPO2RA treatment Raw Data.tif]

**4F3-RSPO2RA**

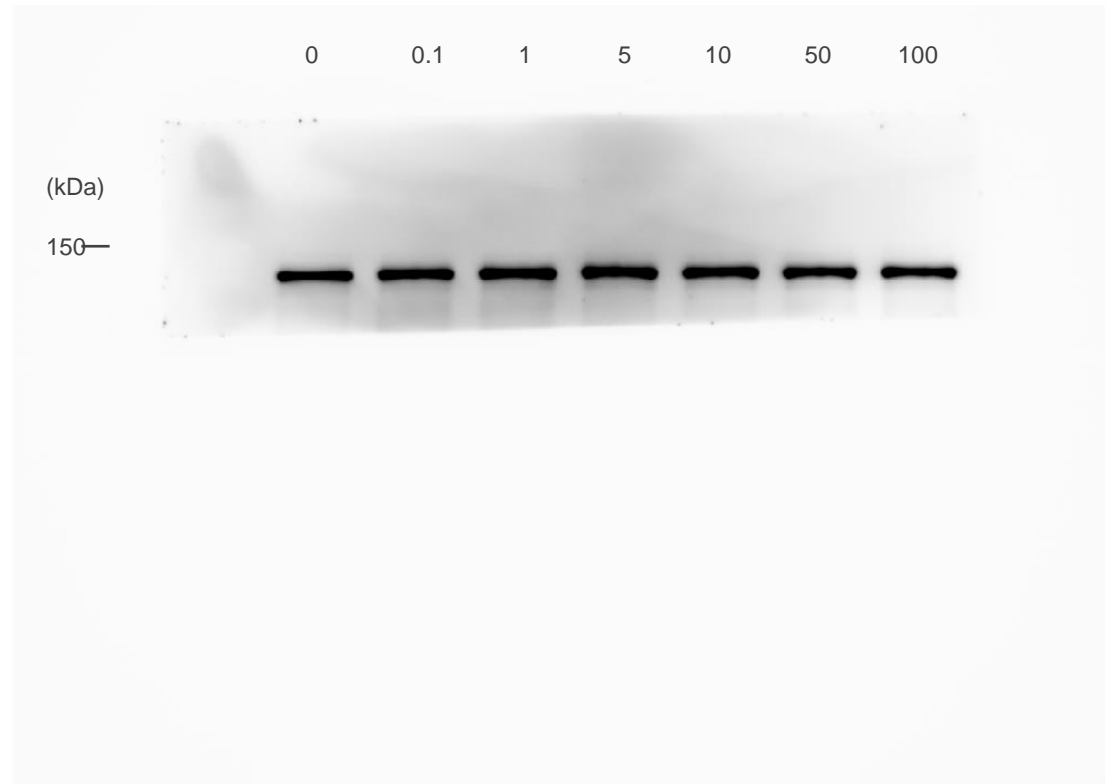

Supplement: Figure 6—source data 1. [file elife-93908-fig6-data1.zip › Figure 6A anti-Vinculin with 4F3-RSPO2RA treatment Labelled Raw Data.pdf]

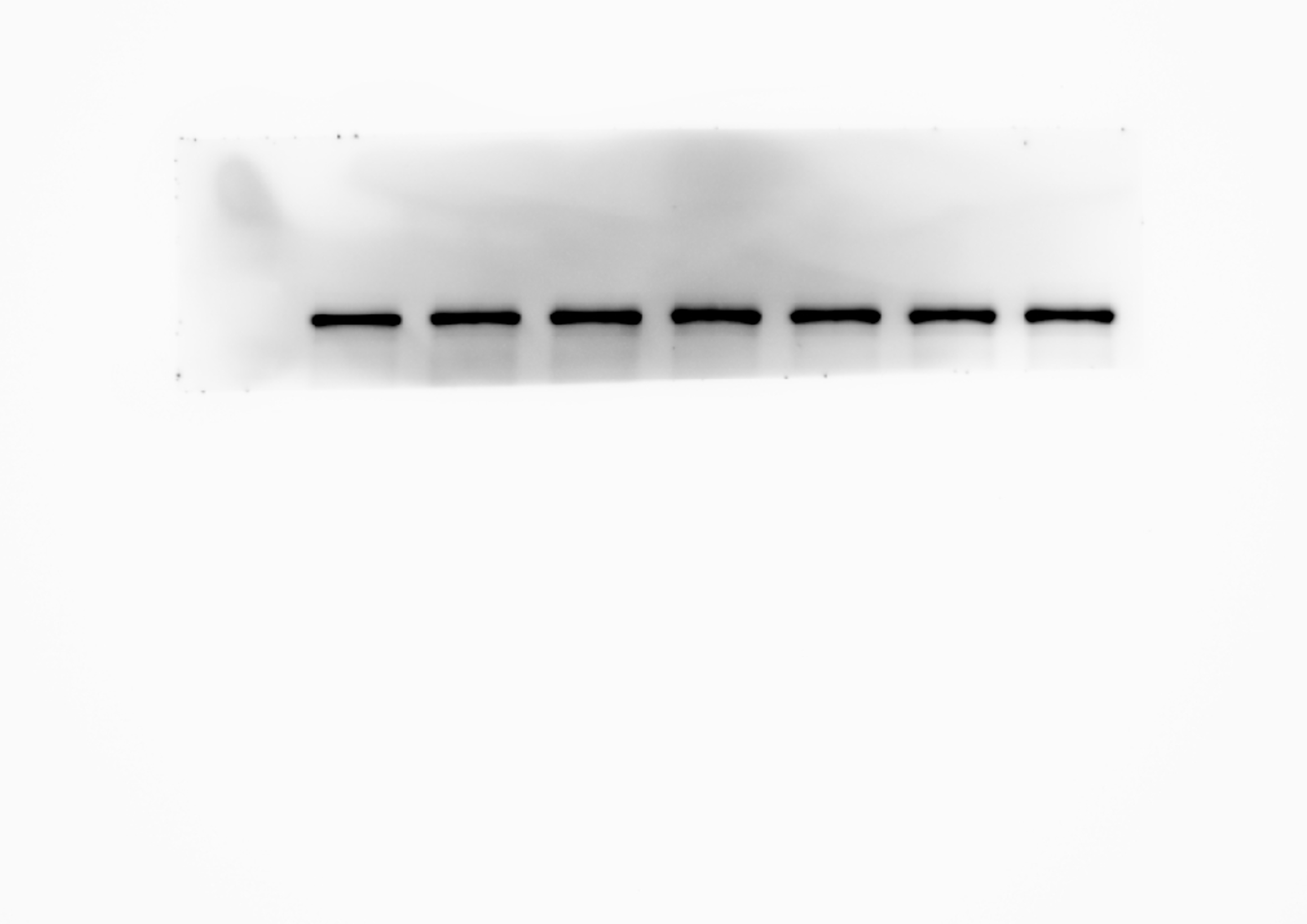

Supplement: Figure 6—source data 1. [file elife-93908-fig6-data1.zip › Figure 6A anti-Vinculin with 4F3-RSPO2RA treatment Raw Data.tif]

### 8G8-RSPO2RA

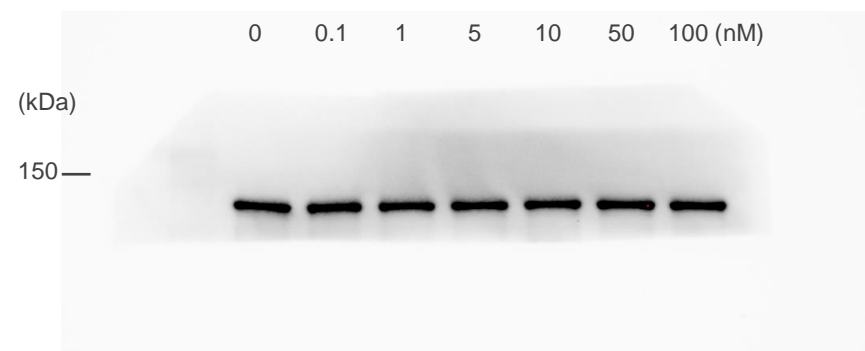

Supplement: Figure 6—source data 1. [file elife-93908-fig6-data1.zip › Figure 6A anti-Vinculin with 8G8-RSPO2RA treatment Labelled Raw Data.pdf]

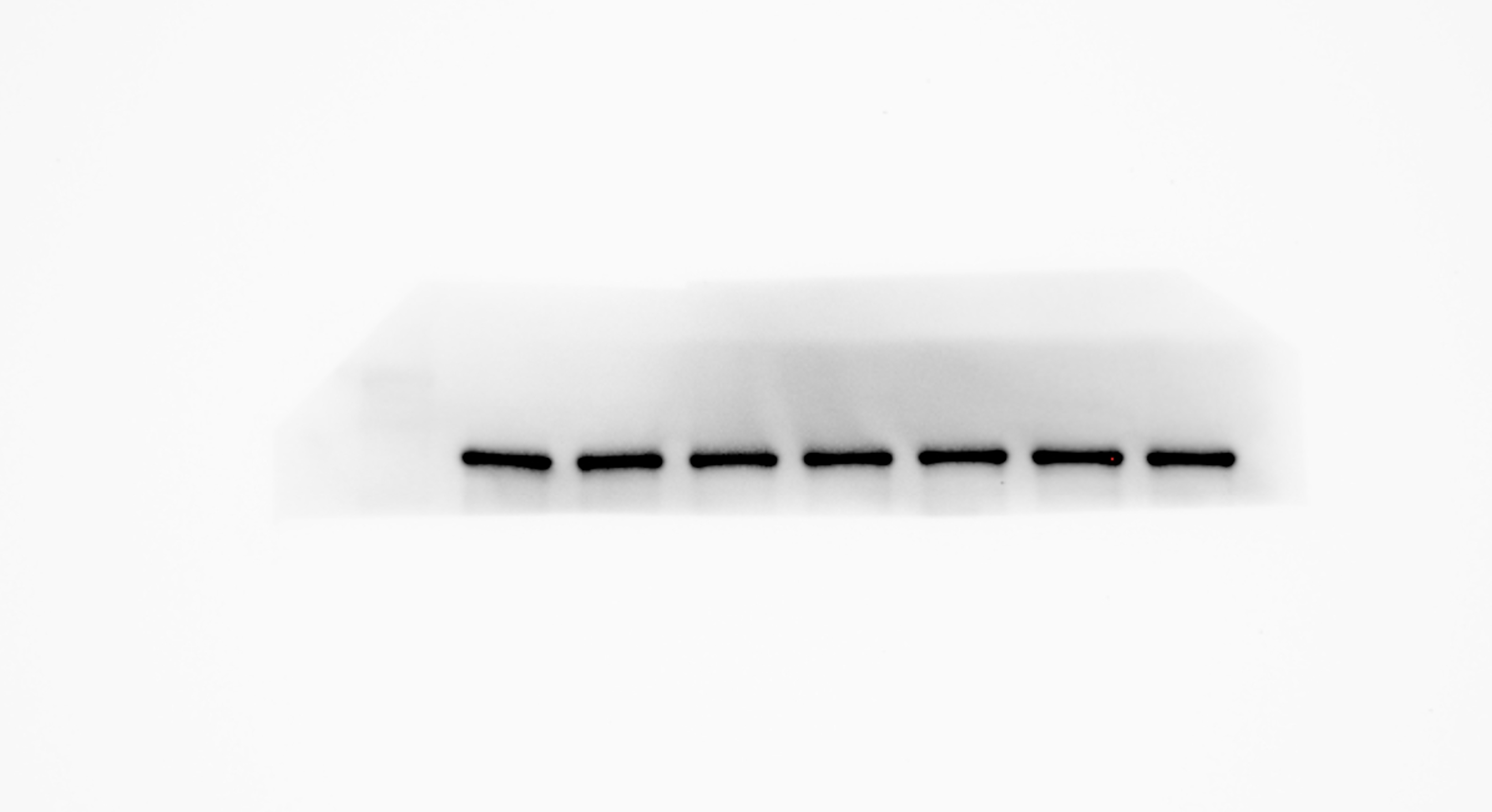

Supplement: Figure 6—source data 1. [file elife-93908-fig6-data1.zip › Figure 6A anti-Vinculin with 8G8-RSPO2RA treatment Raw Data.tif]

**8M24-RSPO2RA**

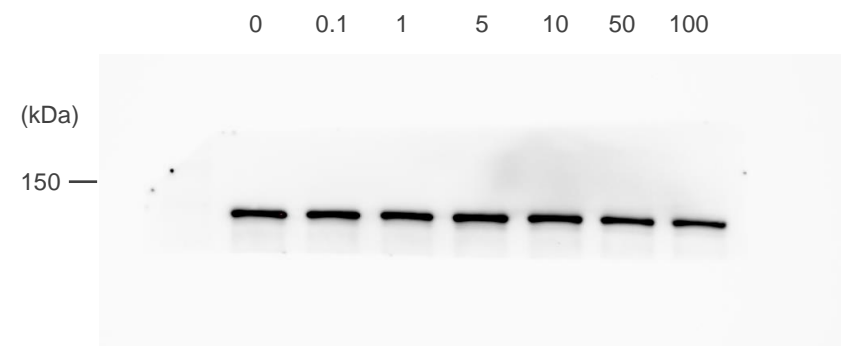

Supplement: Figure 6—source data 1. [file elife-93908-fig6-data1.zip › Figure 6A anti-Vinculin with 8M24-RSPO2RA treatment Labelled Raw Data.pdf]

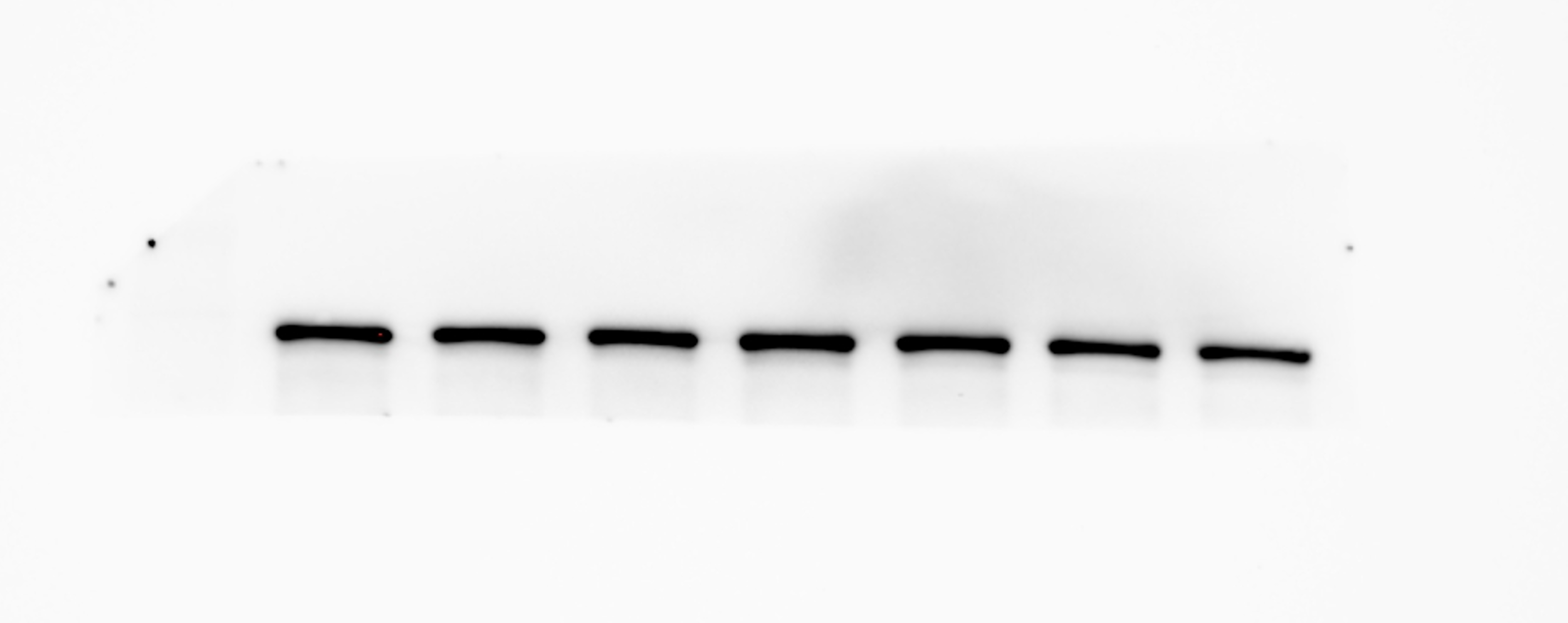

Supplement: Figure 6—source data 1. [file elife-93908-fig6-data1.zip › Figure 6A anti-Vinculin with 8M24-RSPO2RA treatment Raw Data.tif]

**4F3-RSPO2RA**

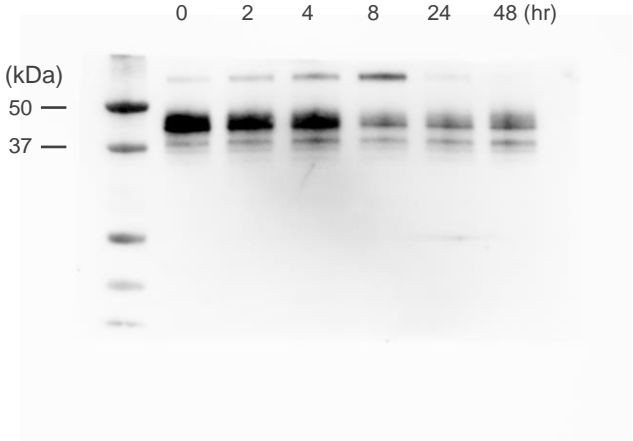

Supplement: Figure 6—source data 2. [file elife-93908-fig6-data2.zip › Figure 6B anti-ASGR1 with 4F3-RSPO2RA treatment Labelled Raw Data.pdf]

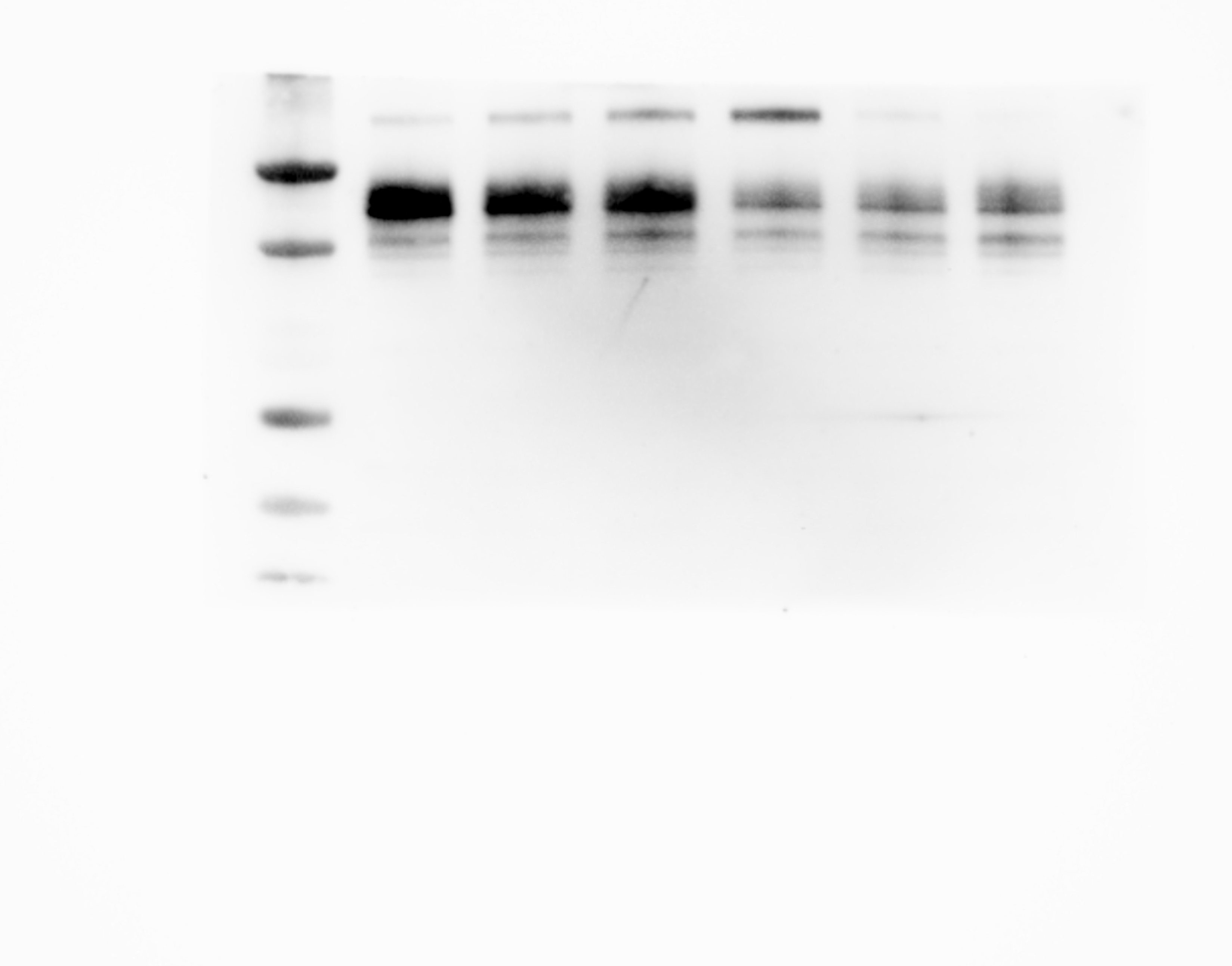

Supplement: Figure 6—source data 2. [file elife-93908-fig6-data2.zip › Figure 6B anti-ASGR1 with 4F3-RSPO2RA treatment Raw Data.tif]

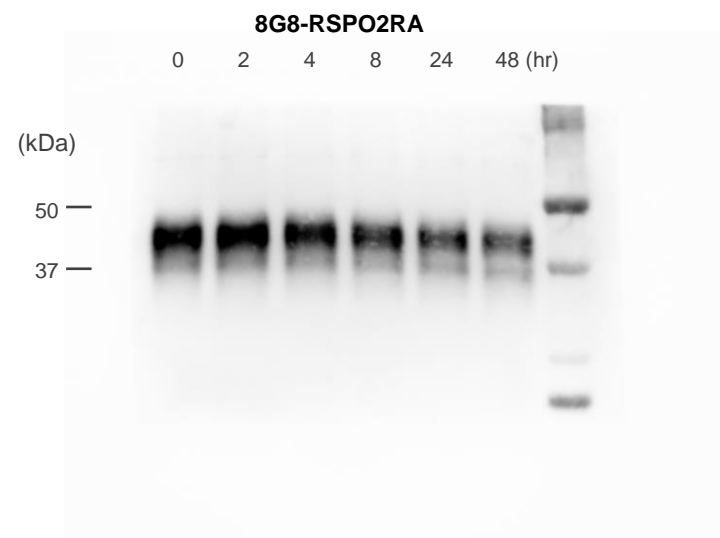

Supplement: Figure 6—source data 2. [file elife-93908-fig6-data2.zip › Figure 6B anti-ASGR1 with 8G8-RSPO2RA treatment Labelled Raw Data.pdf]

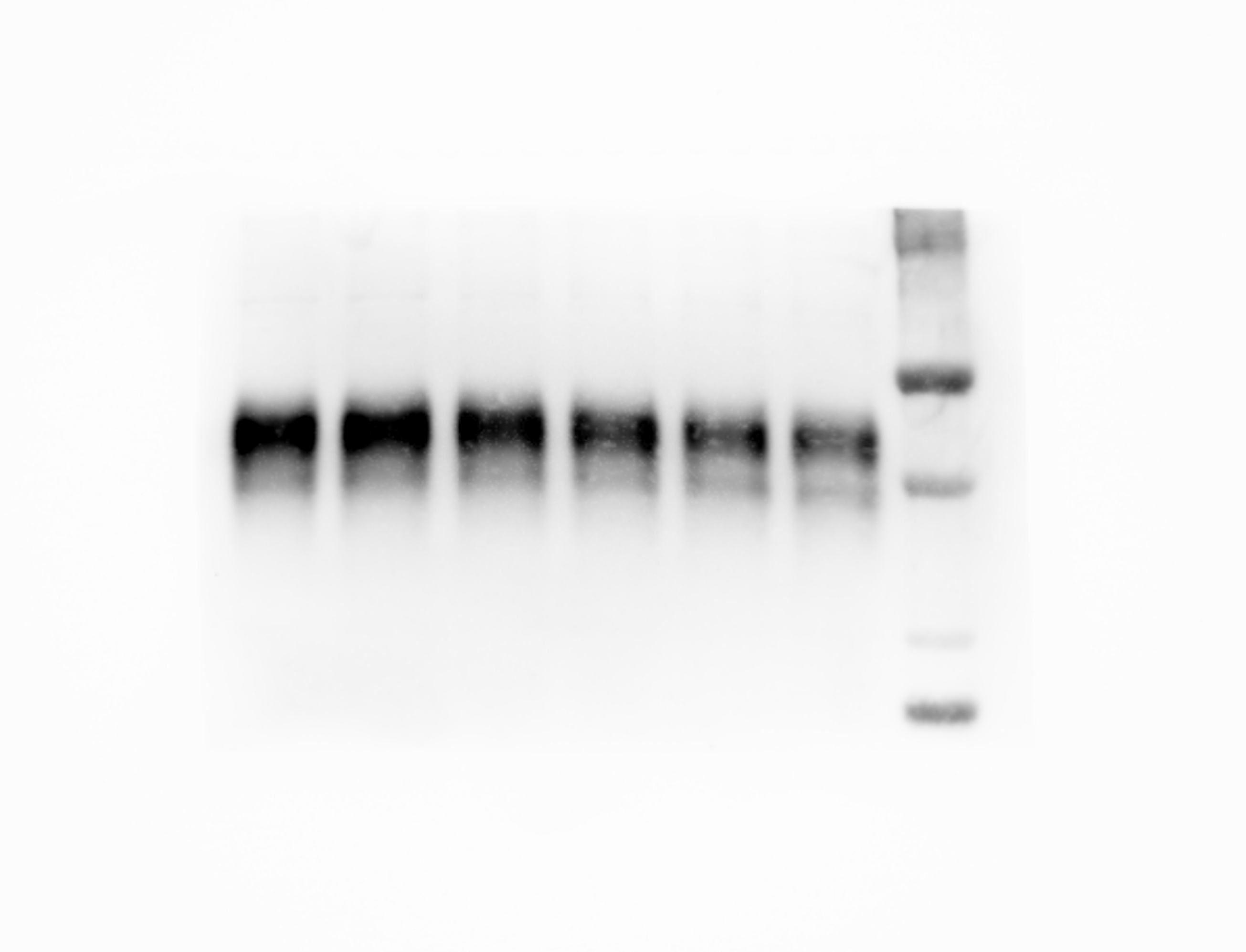

Supplement: Figure 6—source data 2. [file elife-93908-fig6-data2.zip › Figure 6B anti-ASGR1 with 8G8-RSPO2RA treatment Raw Data.tif]

8M24-RSPO2RA

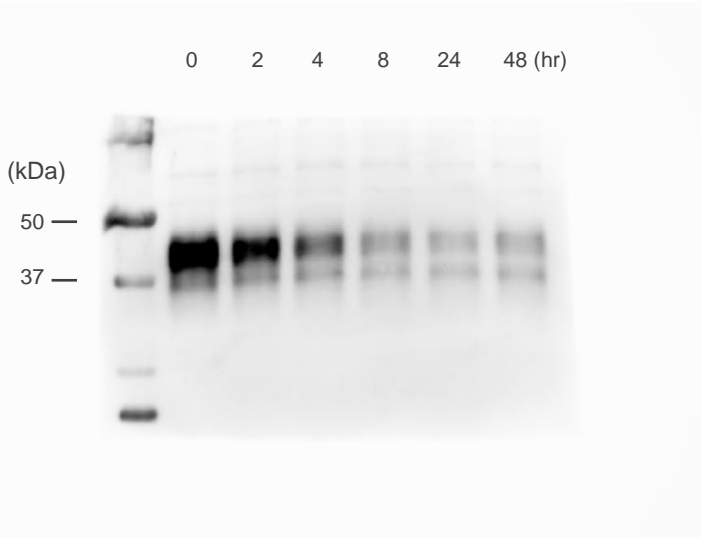

Supplement: Figure 6—source data 2. [file elife-93908-fig6-data2.zip › Figure 6B anti-ASGR1 with 8M24-RSPO2RA treatment Labelled Raw Data.pdf]

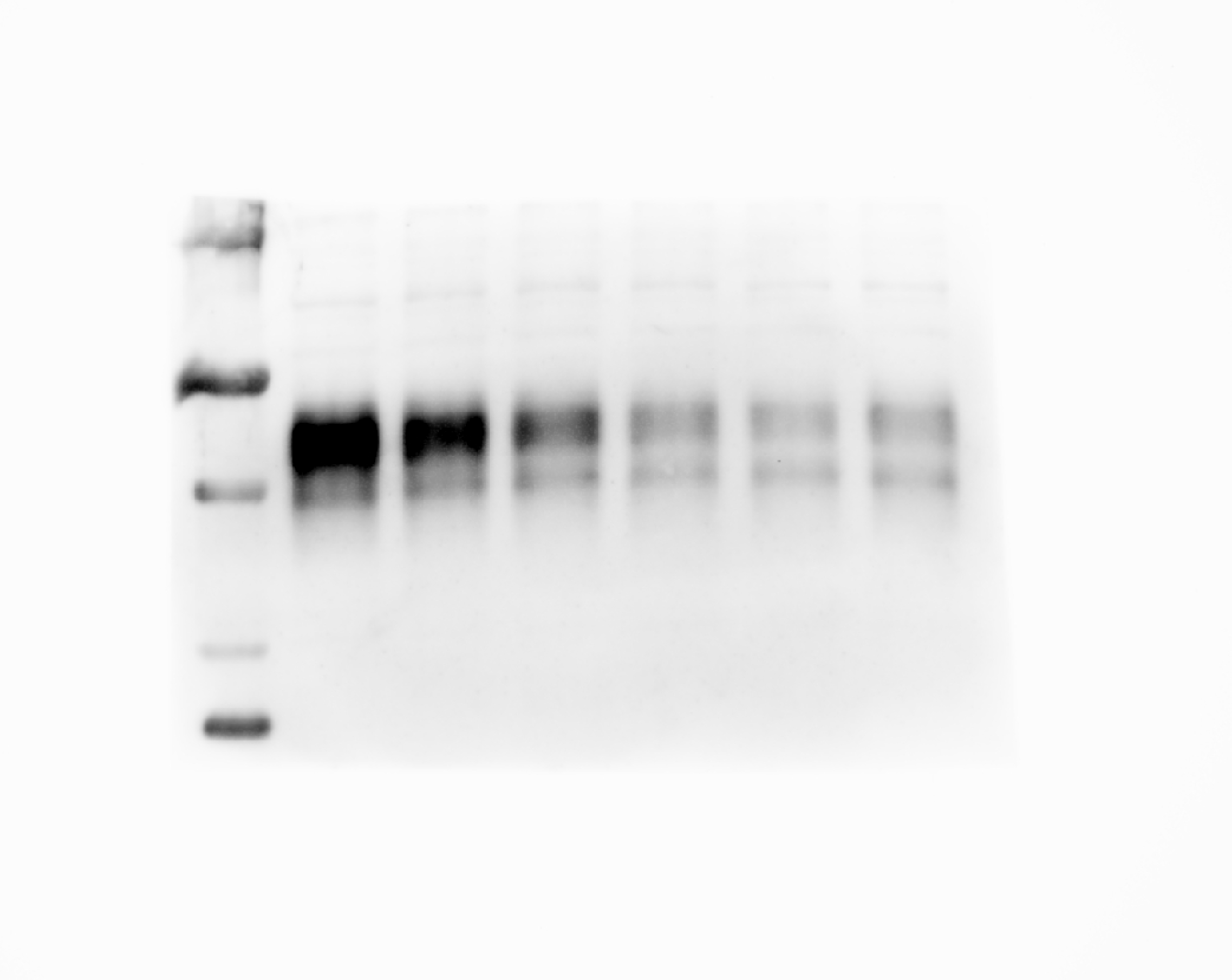

Supplement: Figure 6—source data 2. [file elife-93908-fig6-data2.zip › Figure 6B anti-ASGR1 with 8M24-RSPO2RA treatment Raw Data.tif]

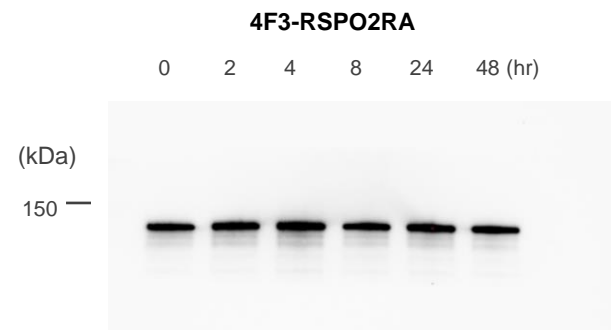

Supplement: Figure 6—source data 2. [file elife-93908-fig6-data2.zip › Figure 6B anti-Vinculin with 4F3-RSPO2RA treatment Labelled Raw Data.pdf]

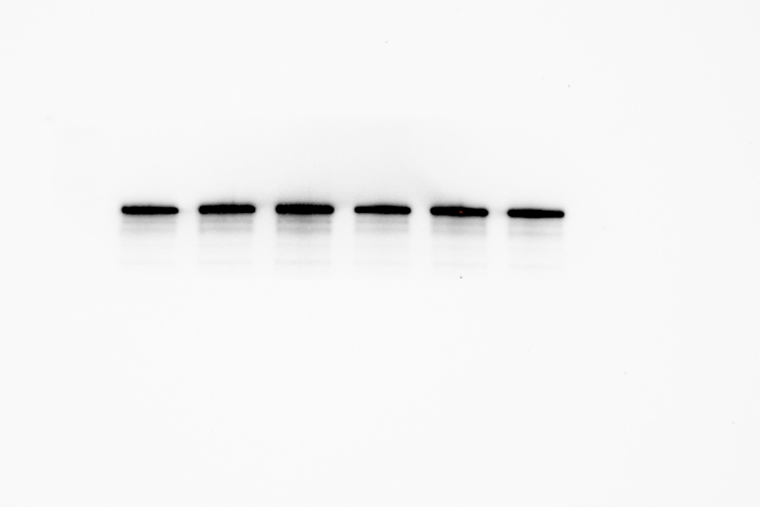

Supplement: Figure 6—source data 2. [file elife-93908-fig6-data2.zip › Figure 6B anti-Vinculin with 4F3-RSPO2RA treatment Raw Data.tif]

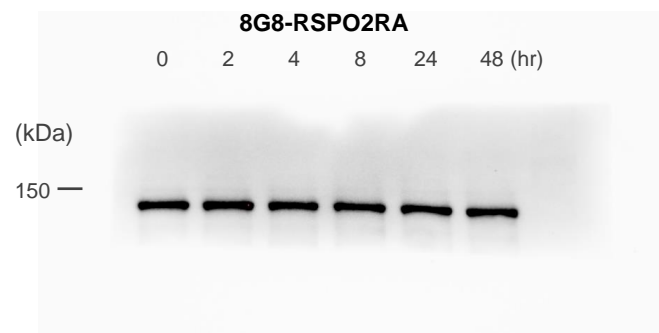

Supplement: Figure 6—source data 2. [file elife-93908-fig6-data2.zip › Figure 6B anti-Vinculin with 8G8-RSPO2RA treatment Labelled Raw Data.pdf]

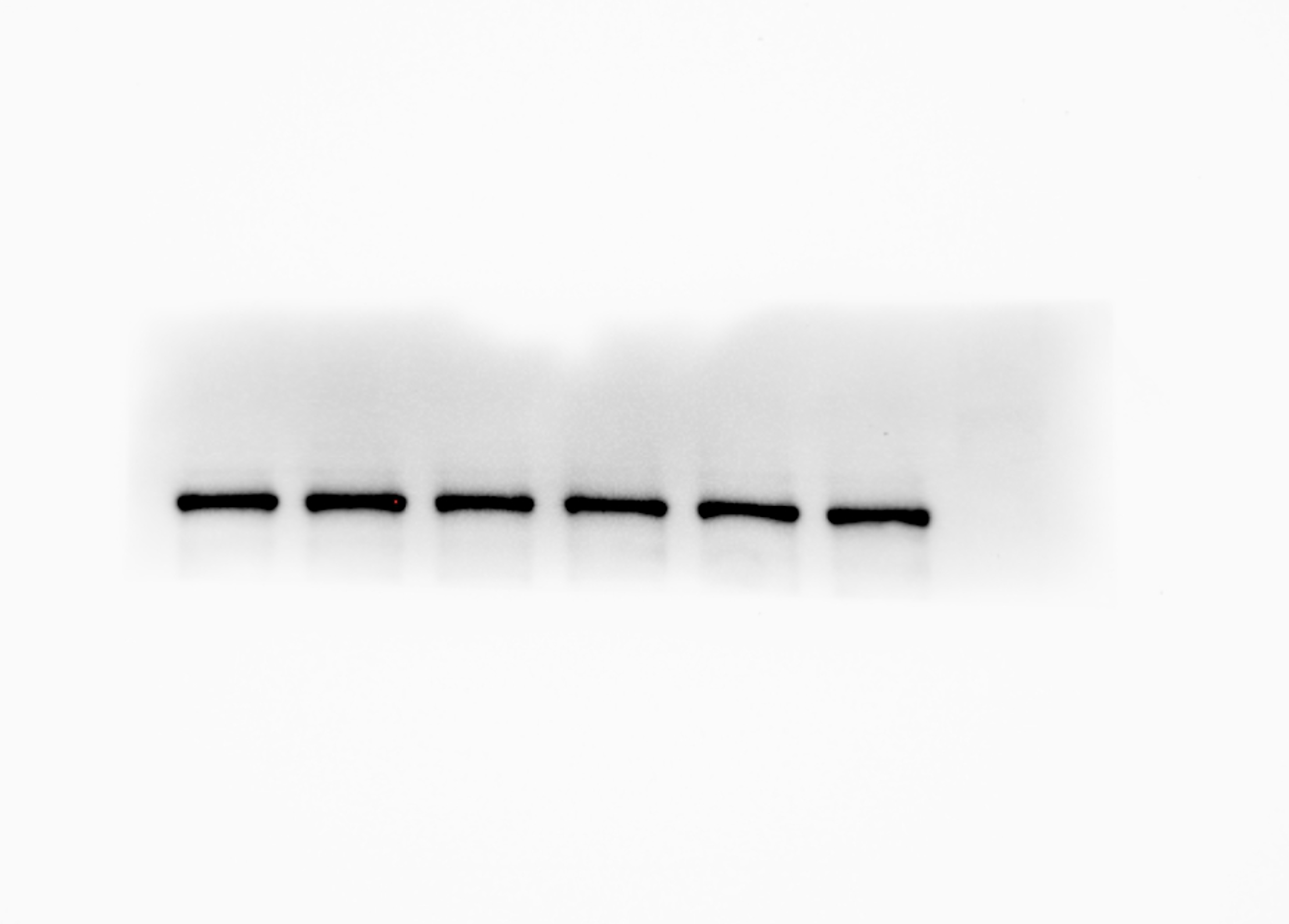

Supplement: Figure 6—source data 2. [file elife-93908-fig6-data2.zip › Figure 6B anti-Vinculin with 8G8-RSPO2RA treatment Raw Data.tif]

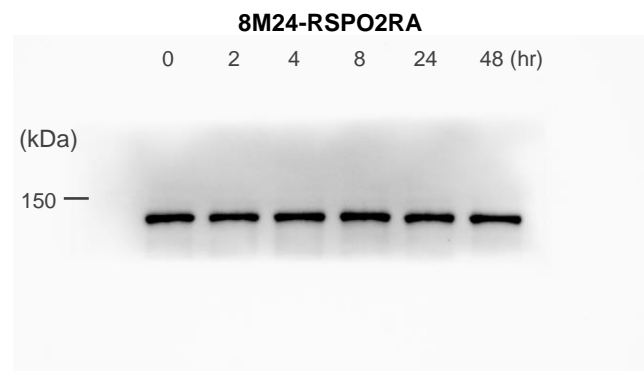

Supplement: Figure 6—source data 2. [file elife-93908-fig6-data2.zip › Figure 6B anti-Vinculin with 8M24-RSPO2RA treatment Labelled Raw Data.pdf]

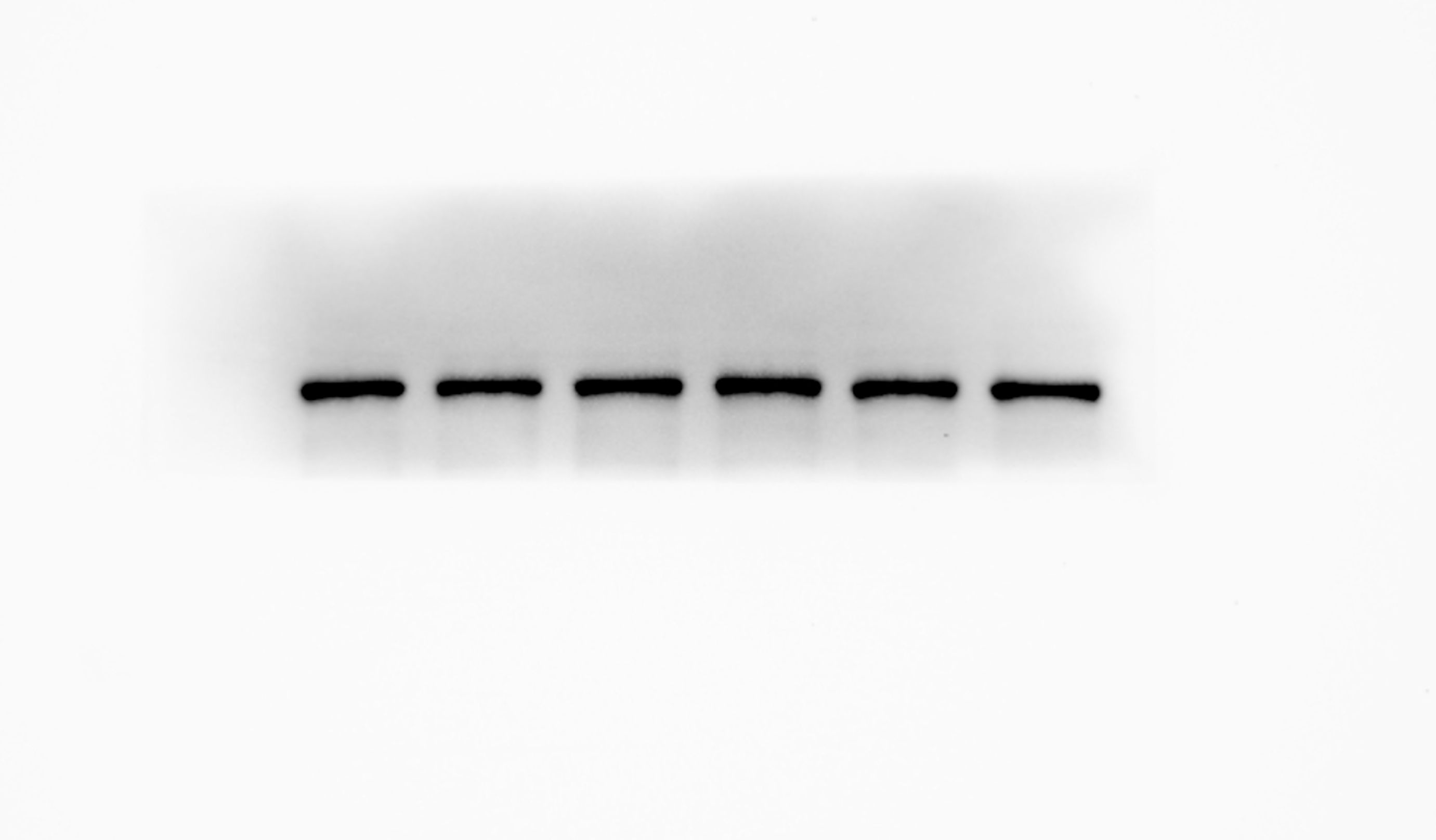

Supplement: Figure 6—source data 2. [file elife-93908-fig6-data2.zip › Figure 6B anti-Vinculin with 8M24-RSPO2RA treatment Raw Data.tif]

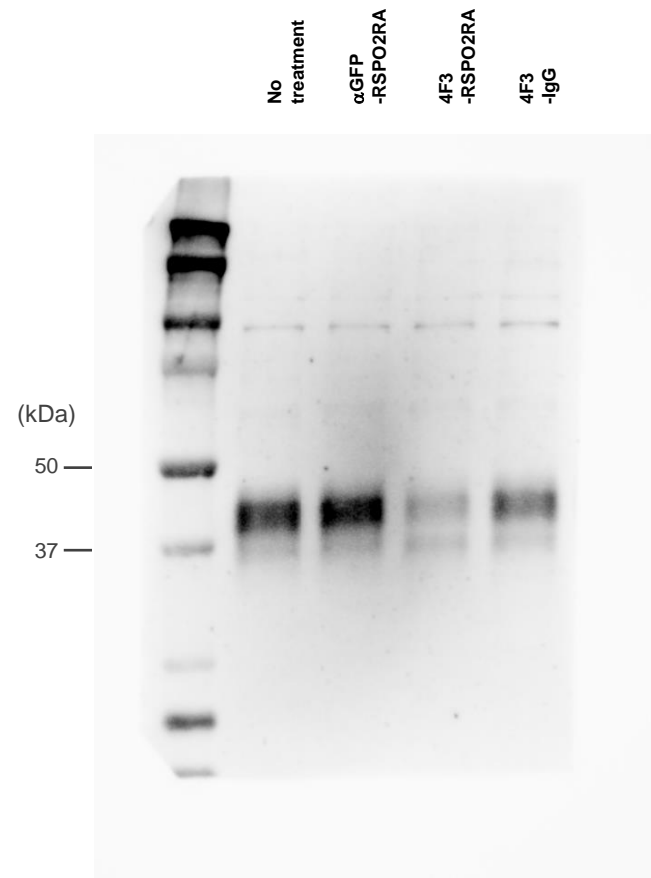

Supplement: Figure 6—source data 3. [file elife-93908-fig6-data3.zip › Figure 6C anti-ASGR1 with 4F3- RSPO2RA or IgG treatment Labelled Raw Data.pdf]

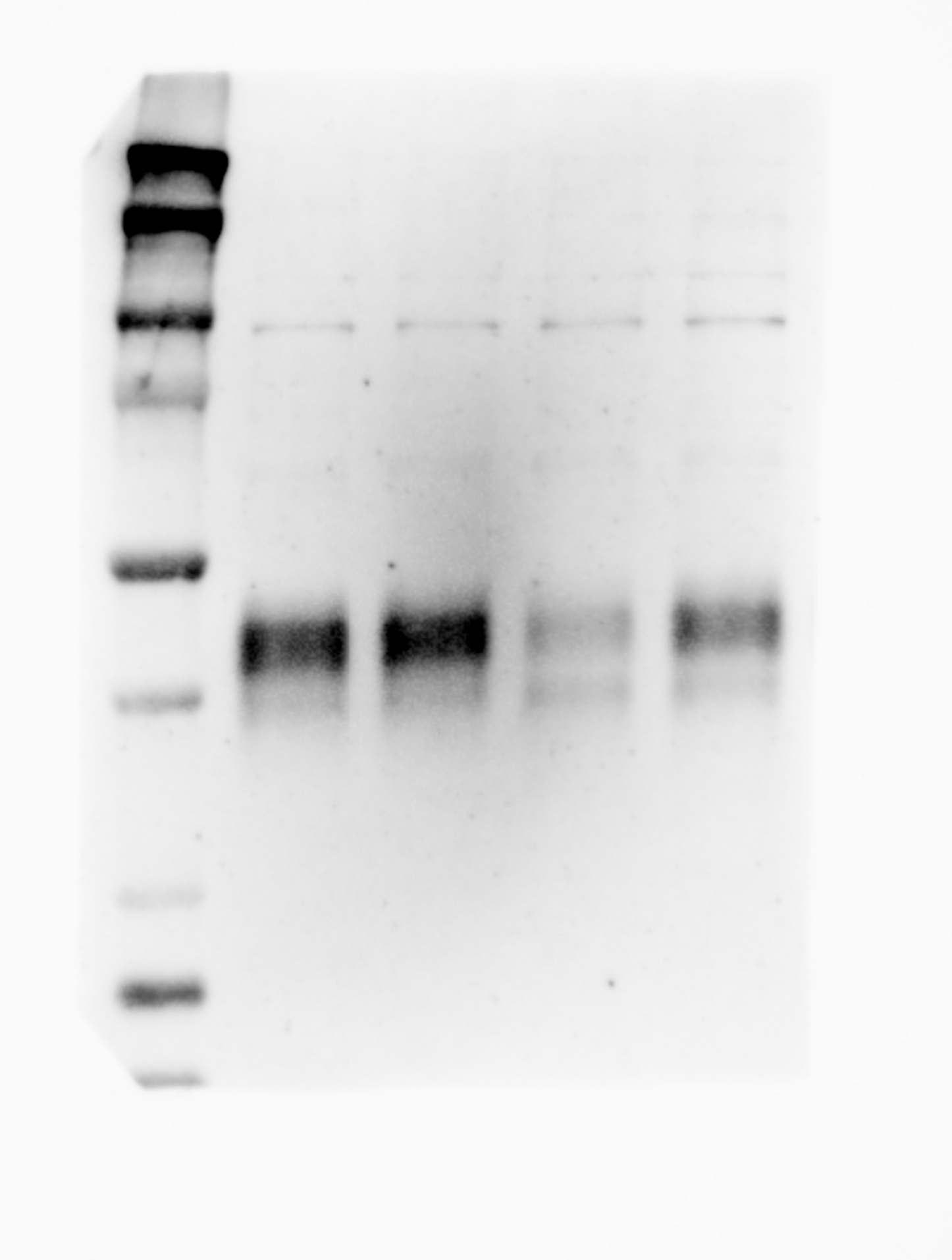

Supplement: Figure 6—source data 3. [file elife-93908-fig6-data3.zip › Figure 6C anti-ASGR1 with 4F3- RSPO2RA or IgG treatment Raw Data.tif]

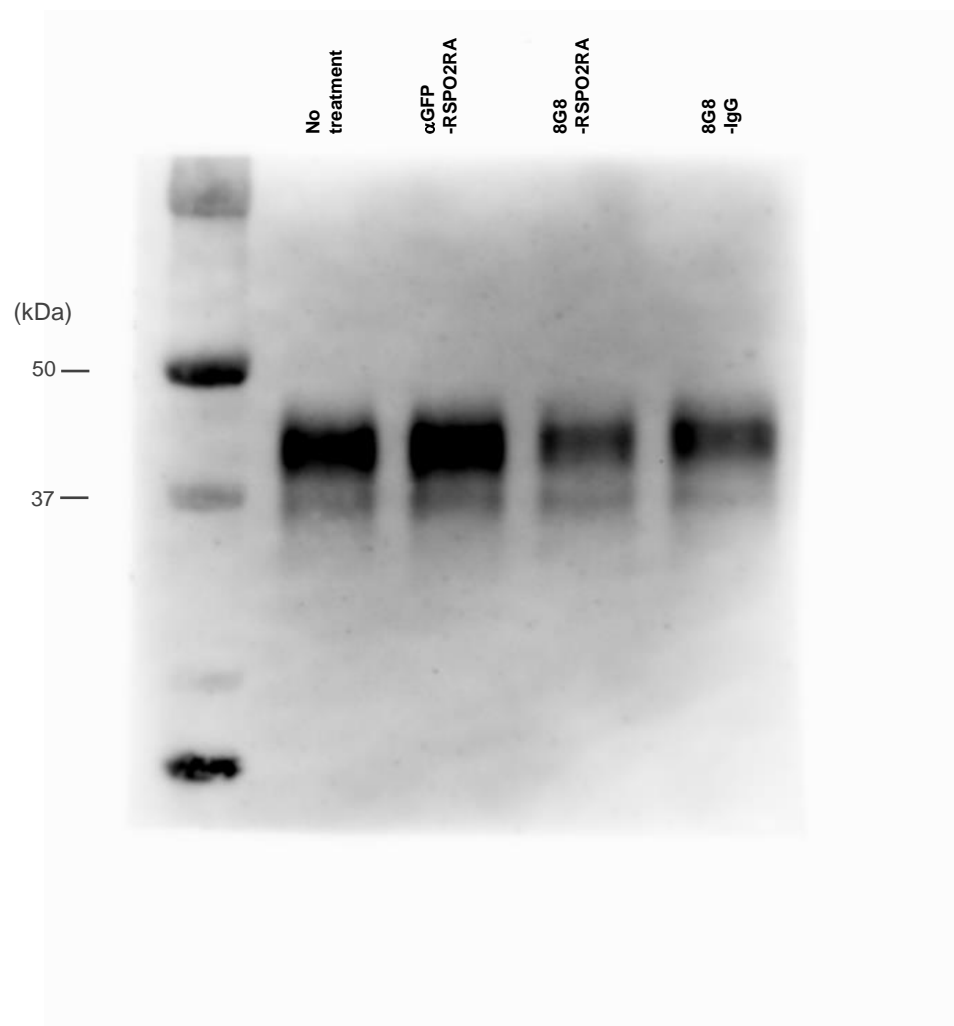

Supplement: Figure 6—source data 3. [file elife-93908-fig6-data3.zip › Figure 6C anti-ASGR1 with 8G8- RSPO2RA or IgG treatment Lablled Raw Data.pdf]

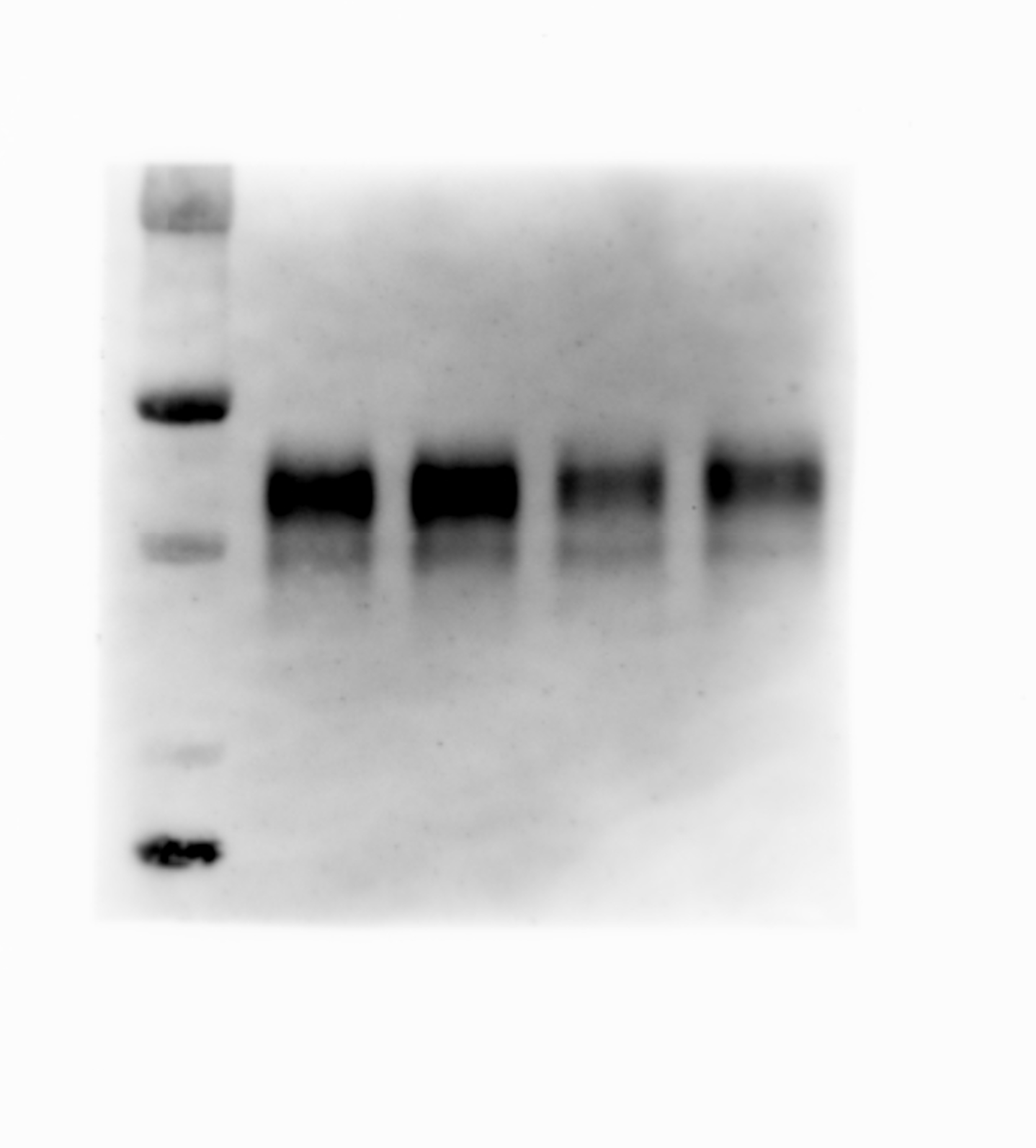

Supplement: Figure 6—source data 3. [file elife-93908-fig6-data3.zip › Figure 6C anti-ASGR1 with 8G8- RSPO2RA or IgG treatment Raw Data.tif]

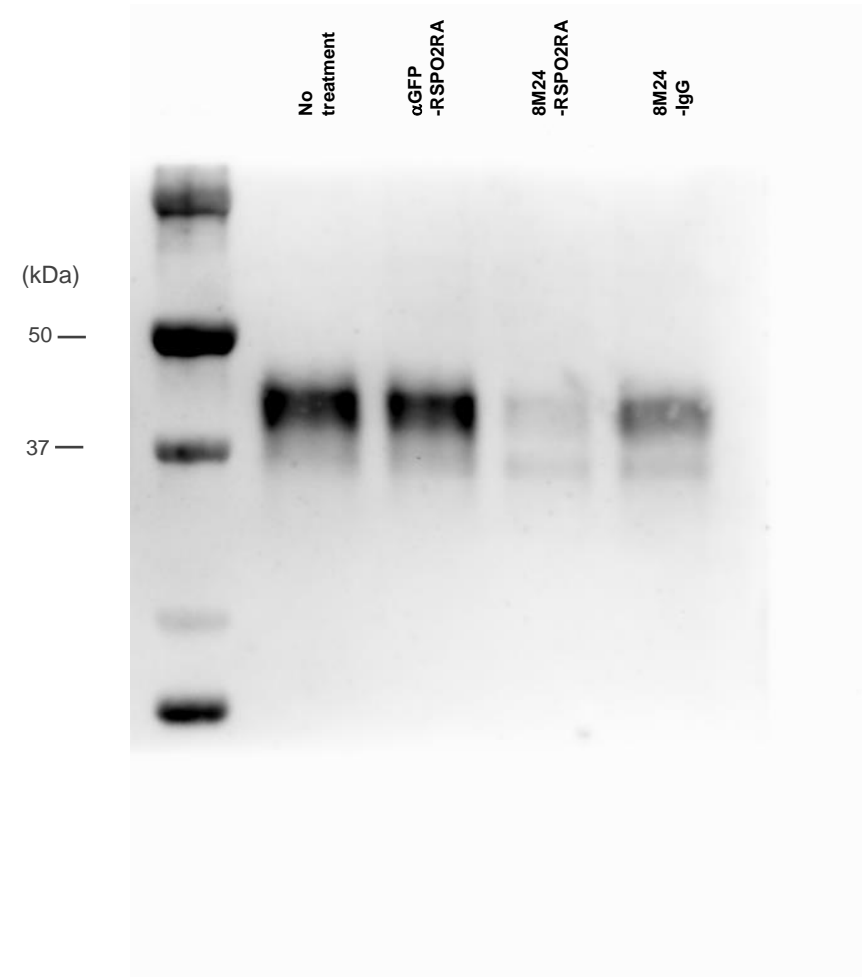

Supplement: Figure 6—source data 3. [file elife-93908-fig6-data3.zip › Figure 6C anti-ASGR1 with 8M24- RSPO2RA or IgG treatment Labelled Raw Data.pdf]

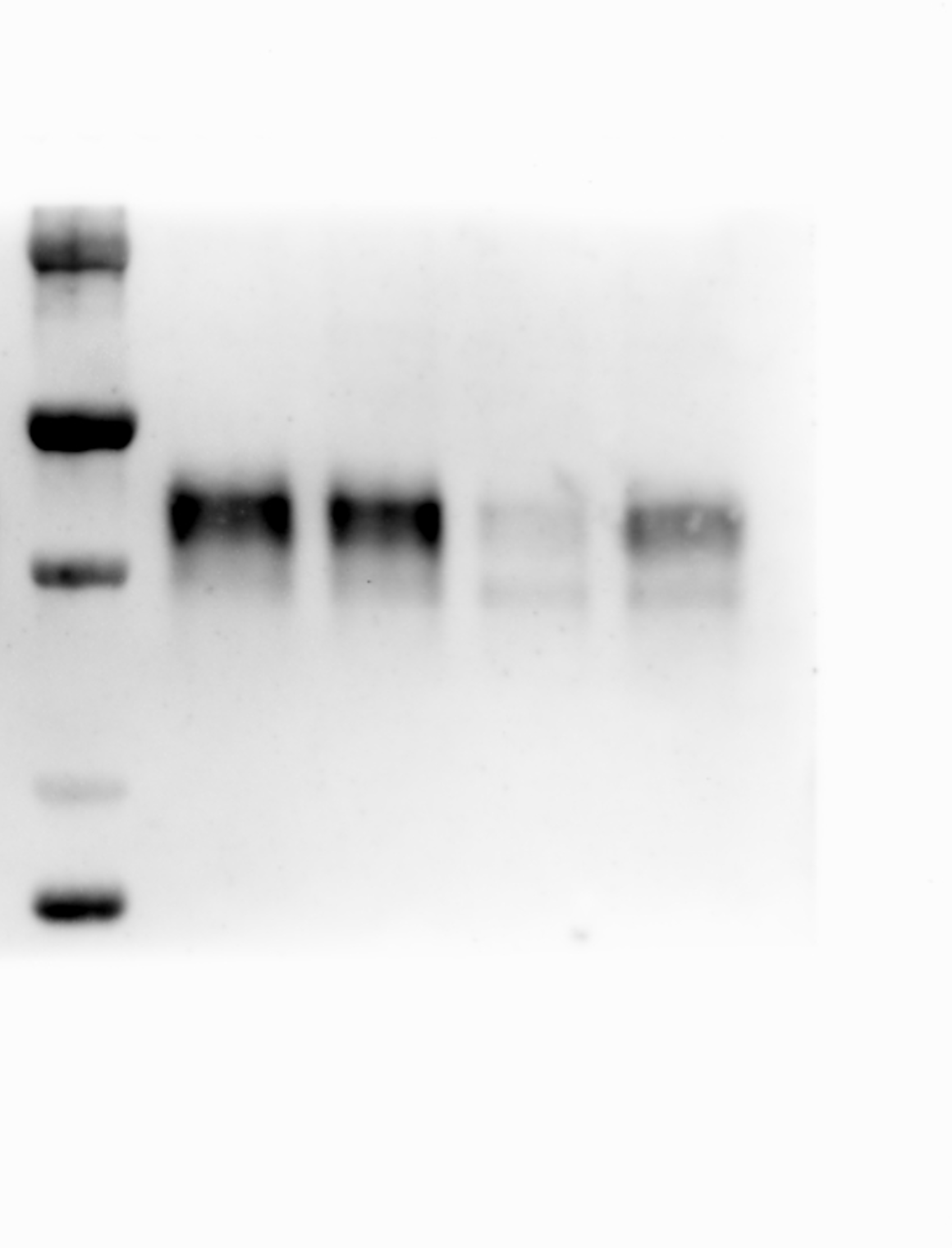

Supplement: Figure 6—source data 3. [file elife-93908-fig6-data3.zip › Figure 6C anti-ASGR1 with 8M24- RSPO2RA or IgG treatment Raw Data.tif]

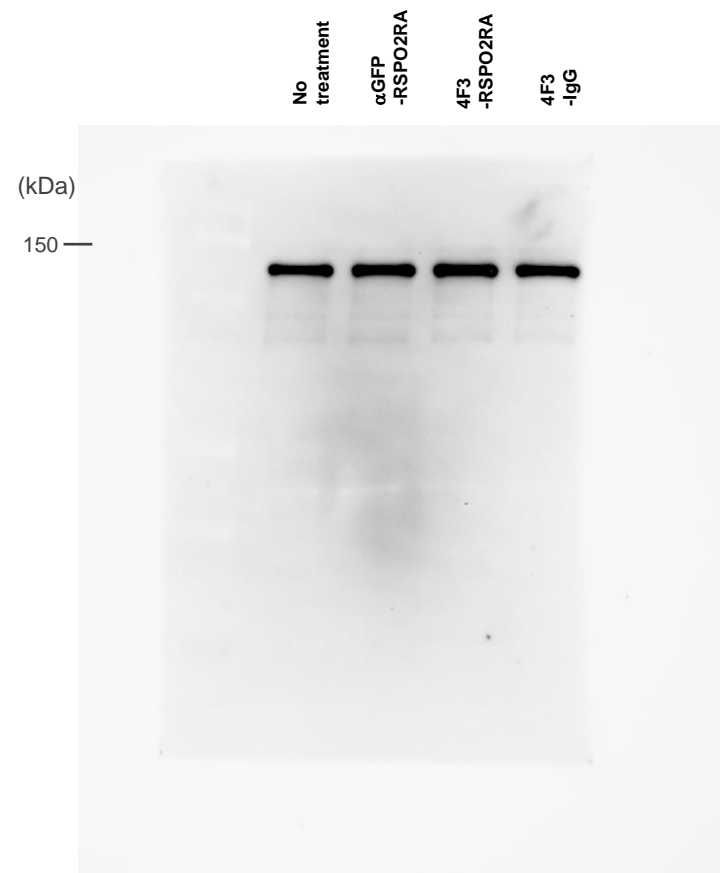

Supplement: Figure 6—source data 3. [file elife-93908-fig6-data3.zip › Figure 6C anti-Vinculin with 4F3- RSPO2RA or IgG treatment Lablled Raw Data.pdf]

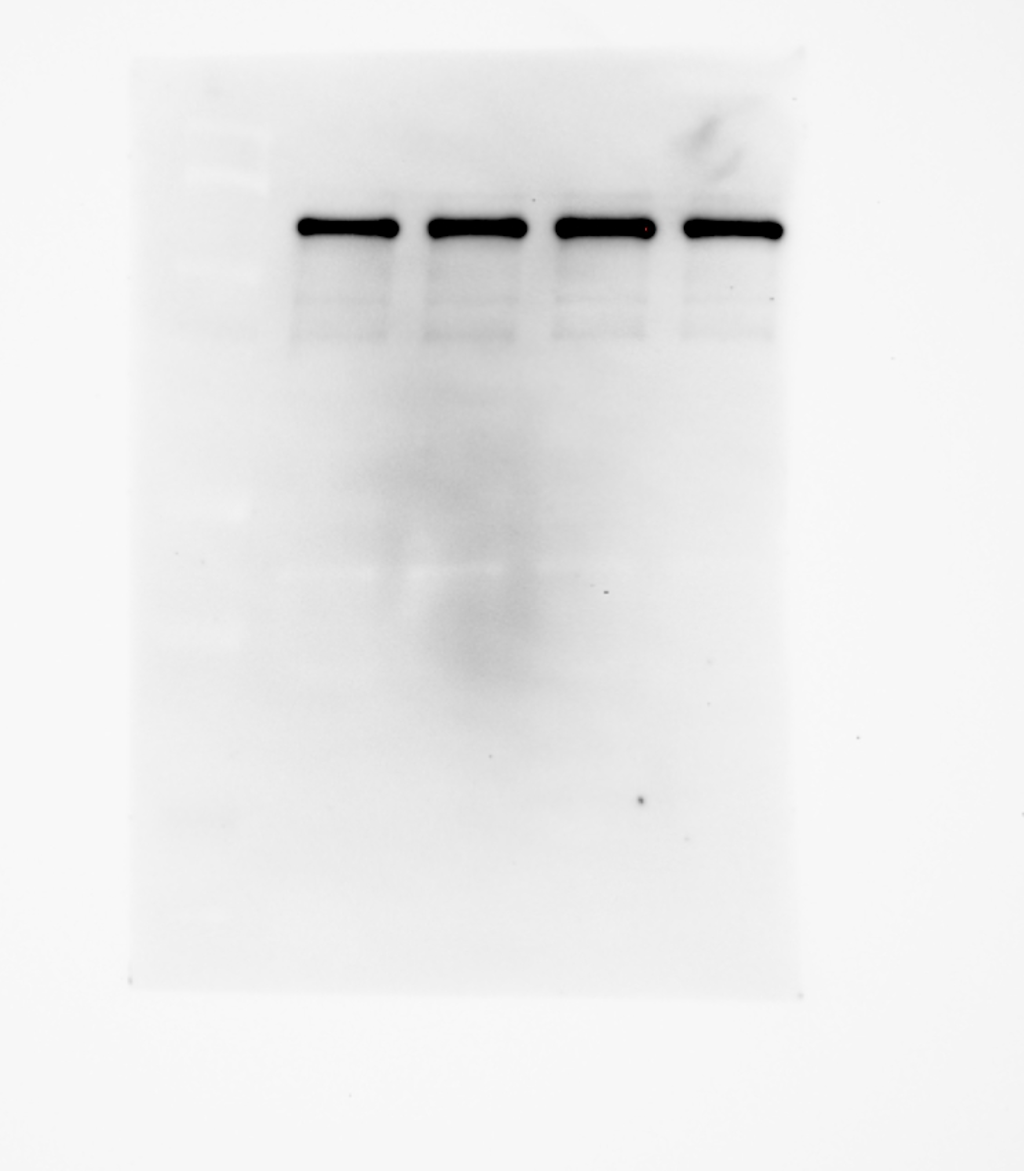

Supplement: Figure 6—source data 3. [file elife-93908-fig6-data3.zip › Figure 6C anti-Vinculin with 4F3- RSPO2RA or IgG treatment Raw Data.tif]

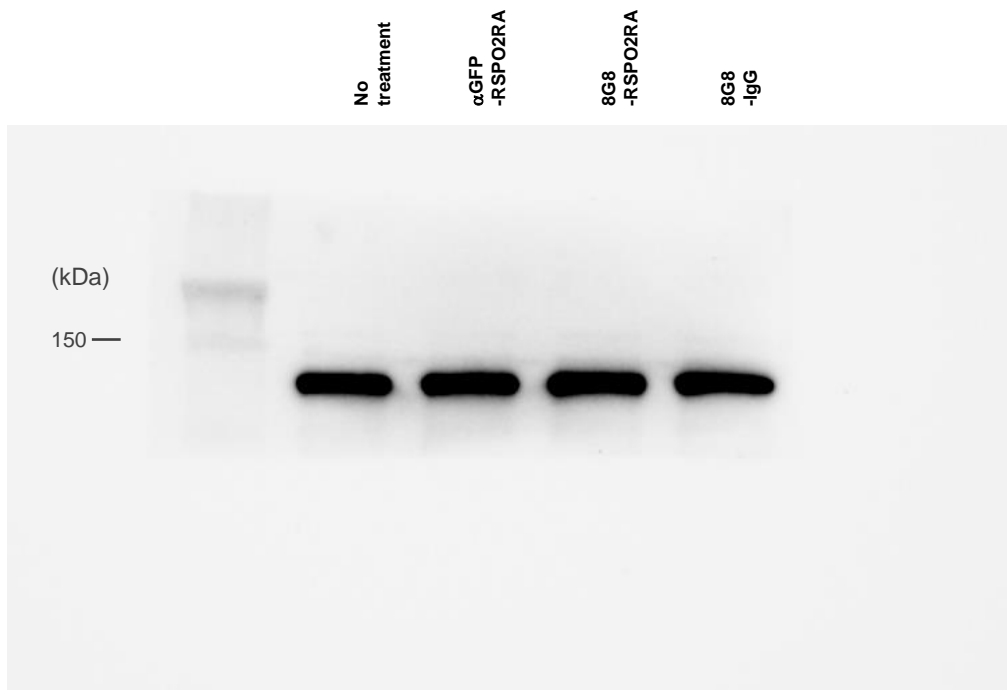

Supplement: Figure 6—source data 3. [file elife-93908-fig6-data3.zip › Figure 6C anti-Vinculin with 8G8- RSPO2RA or IgG treatment Lablled Raw Data.pdf]

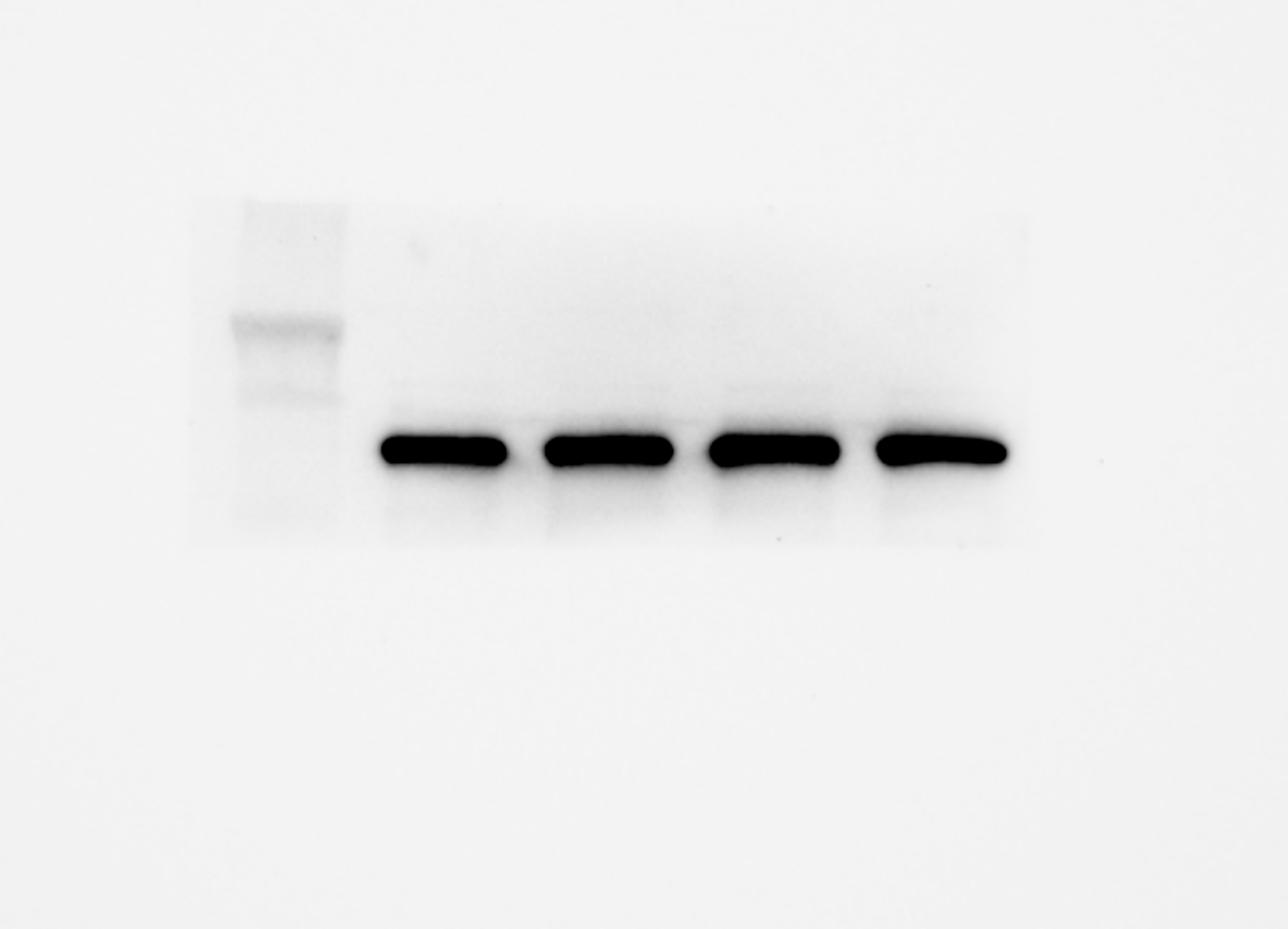

Supplement: Figure 6—source data 3. [file elife-93908-fig6-data3.zip › Figure 6C anti-Vinculin with 8G8- RSPO2RA or IgG treatment Raw Data.tif]

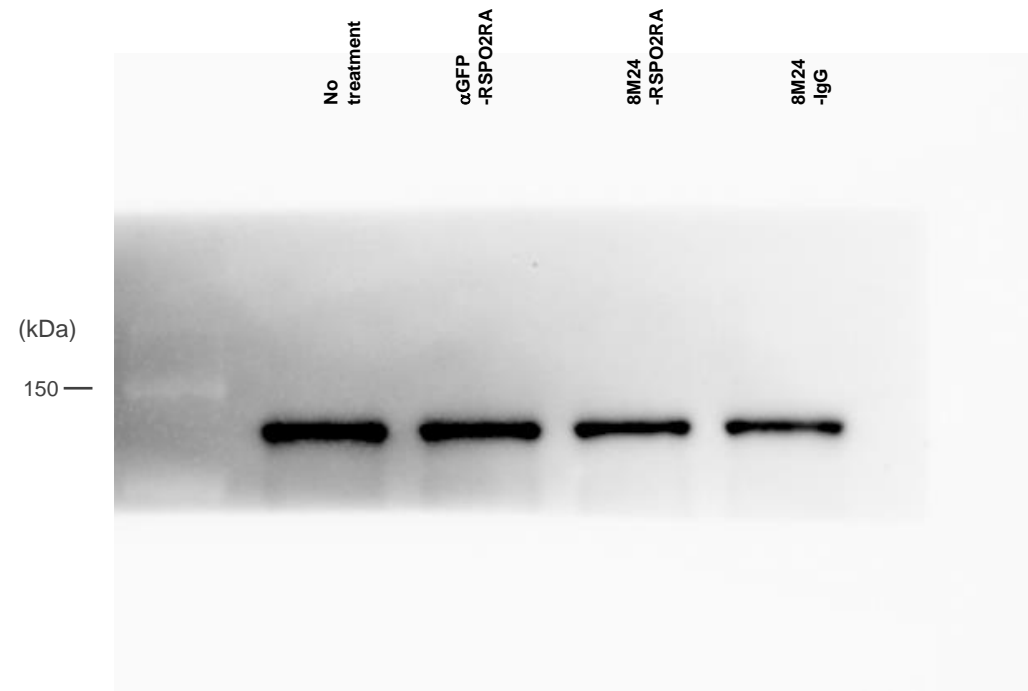

Supplement: Figure 6—source data 3. [file elife-93908-fig6-data3.zip › Figure 6C anti-Vinculin with 8M24- RSPO2RA or IgG treatment Lablled Raw Data.pdf]

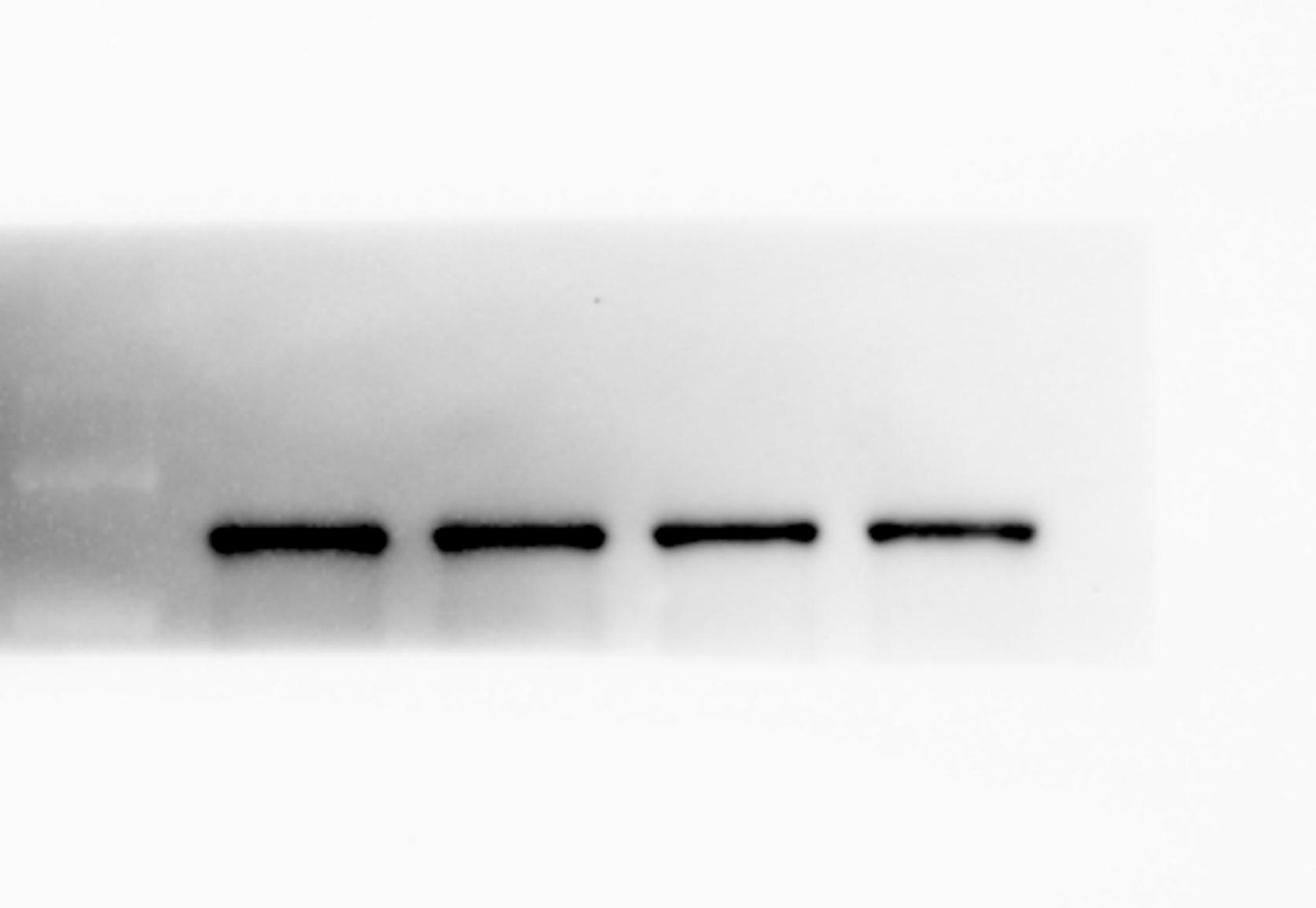

Supplement: Figure 6—source data 3. [file elife-93908-fig6-data3.zip › Figure 6C anti-Vinculin with 8M24- RSPO2RA or IgG treatment Raw Data.tif]

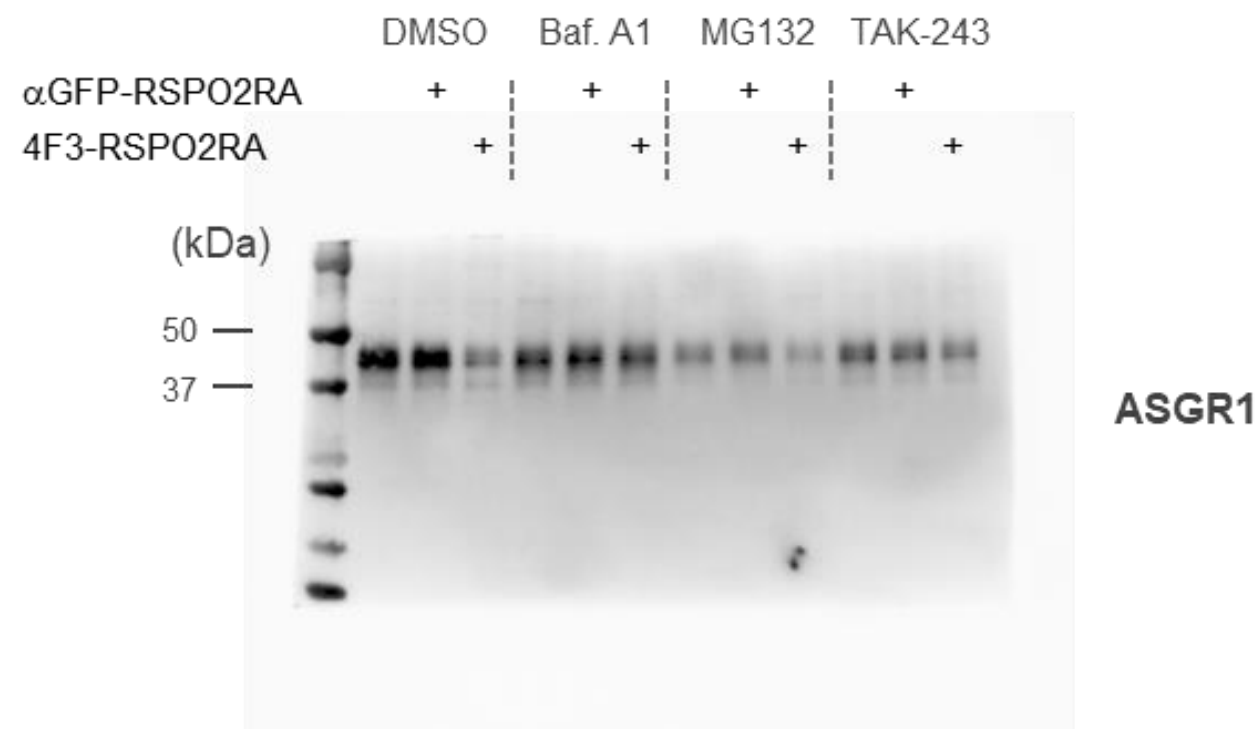

Supplement: Figure 6—source data 4. [file elife-93908-fig6-data4.zip › Figure 6D anti-ASGR1 with 4F3-RSPO2RA treatment Labelled Raw Data.pdf]

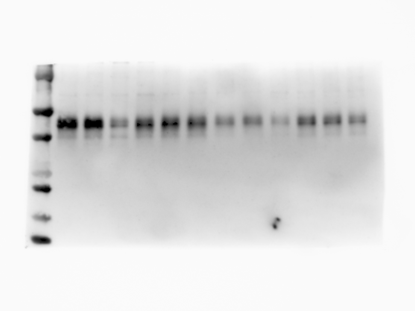

Supplement: Figure 6—source data 4. [file elife-93908-fig6-data4.zip › Figure 6D anti-ASGR1 with 4F3-RSPO2RA treatment Raw Data.tif]

|                      | DMSO | Baf. A1 | MG132 | TAK-243 |
|----------------------|------|---------|-------|---------|
| $\alpha$ GFP-RSP02RA | +    | +       | +     | +       |
| 8G8-RSP02RA          | +    | +       | +     | +       |

(kDa)

50 —

37 —

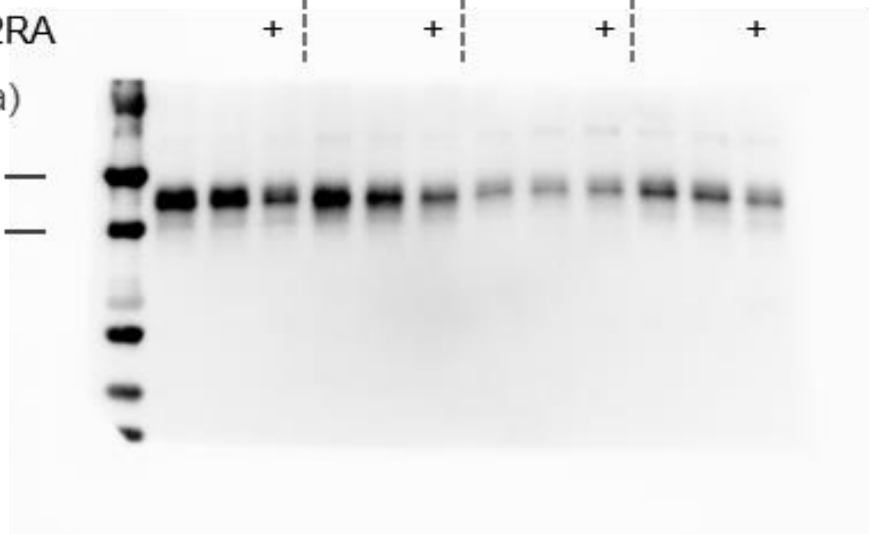

**ASGR1**

Supplement: Figure 6—source data 4. [file elife-93908-fig6-data4.zip › Figure 6D anti-ASGR1 with 8G8-RSPO2RA treatment Labelled Raw Data.pdf]

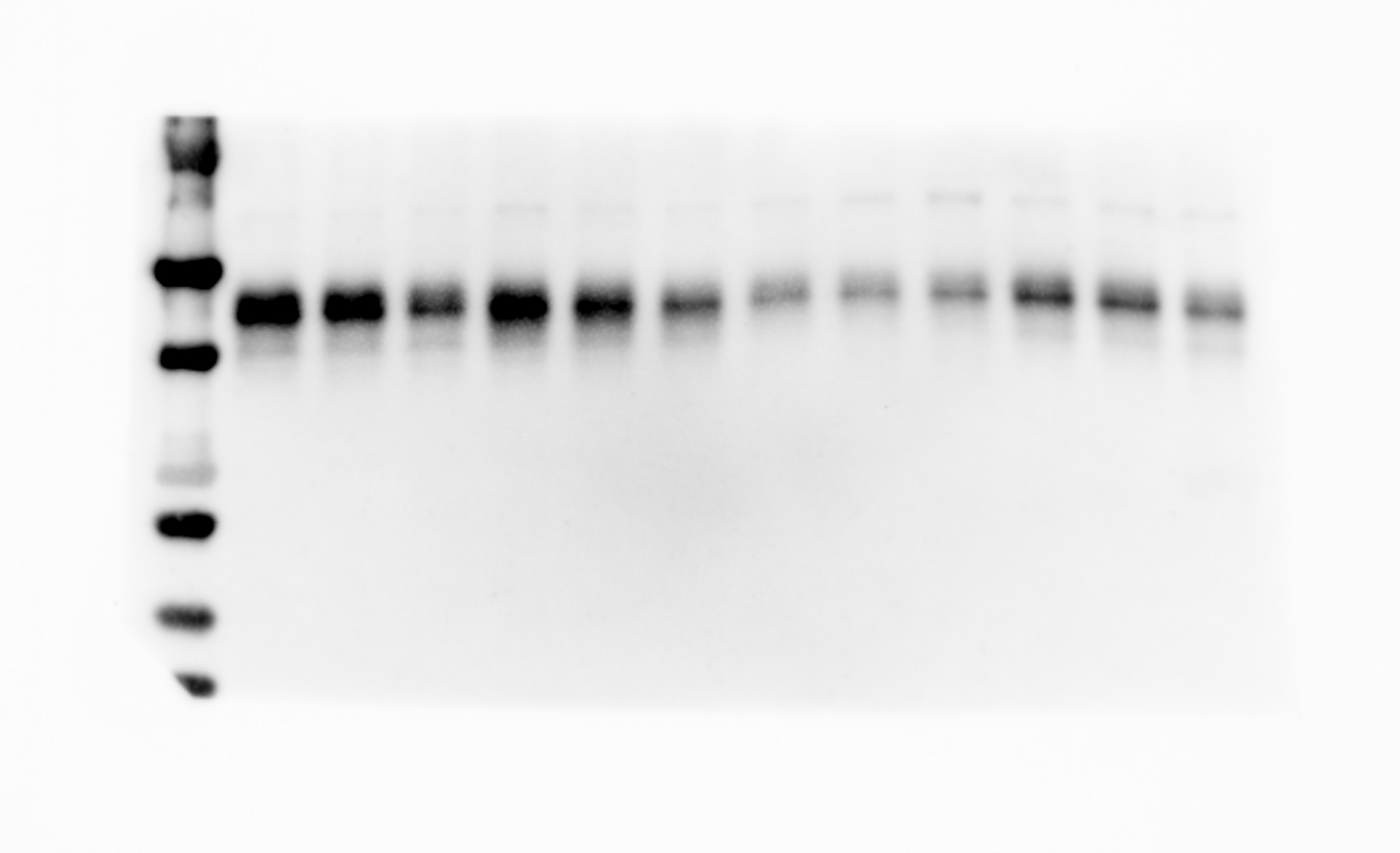

Supplement: Figure 6—source data 4. [file elife-93908-fig6-data4.zip › Figure 6D anti-ASGR1 with 8G8-RSPO2RA treatment Raw Data.tif]

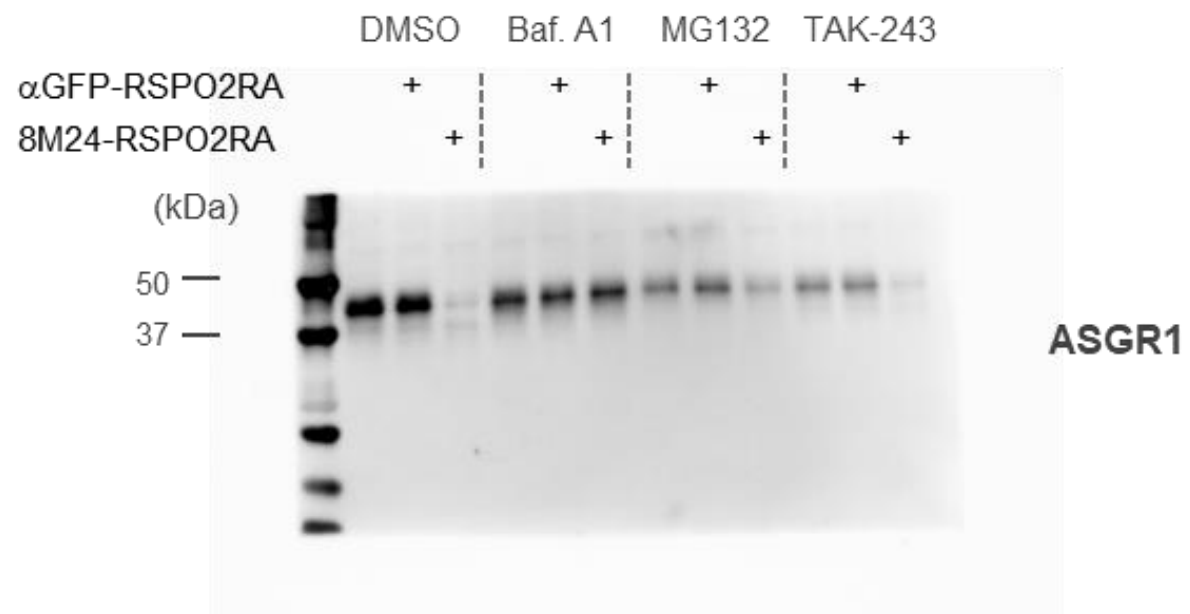

Supplement: Figure 6—source data 4. [file elife-93908-fig6-data4.zip › Figure 6D anti-ASGR1 with 8M24-RSPO2RA treatment Labelled Raw Data.pdf]

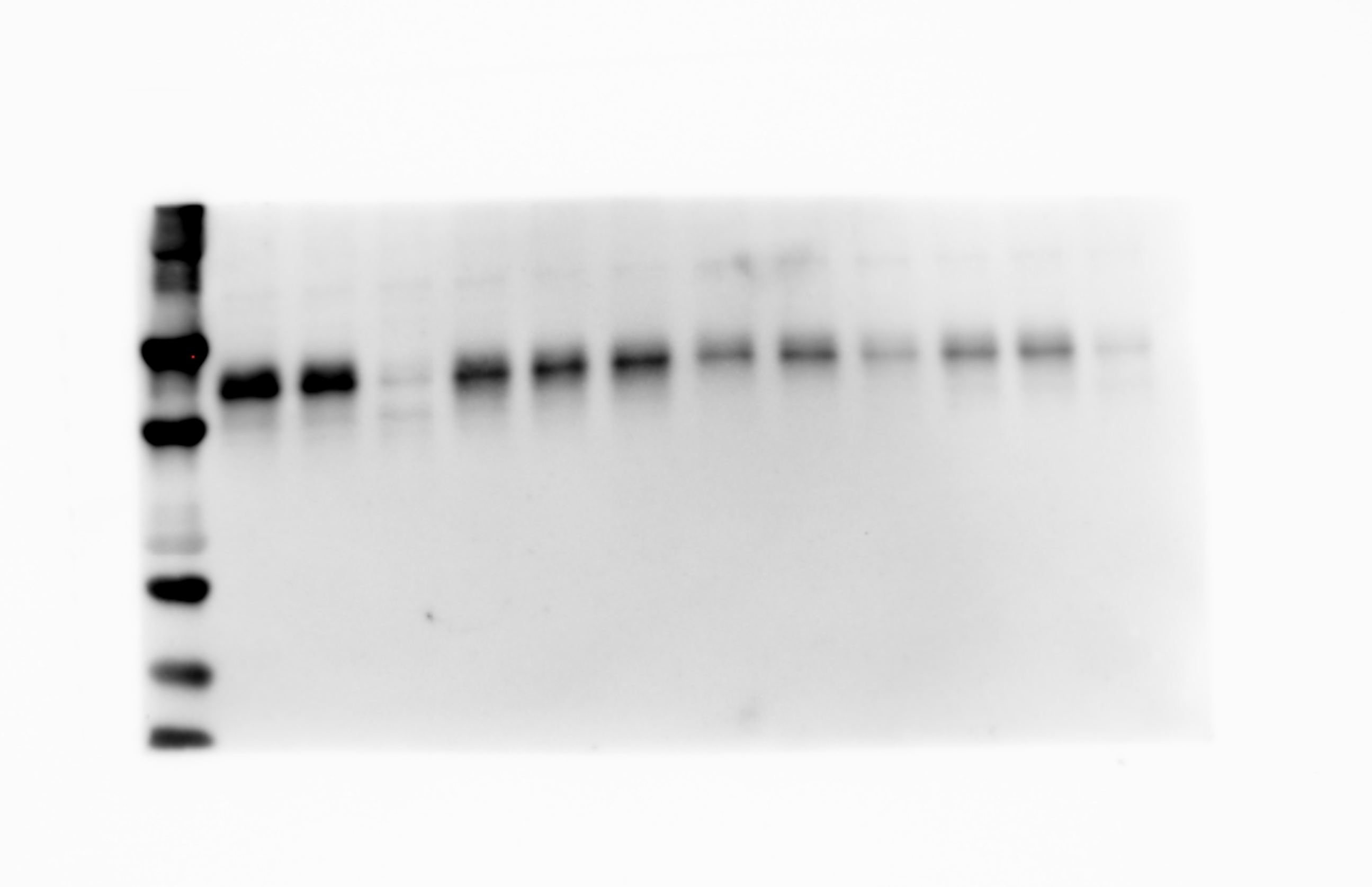

Supplement: Figure 6—source data 4. [file elife-93908-fig6-data4.zip › Figure 6D anti-ASGR1 with 8M24-RSPO2RA treatment Raw Data.tif]

|                      | DMSO | Baf. A1 | MG132 | TAK-243 |
|----------------------|------|---------|-------|---------|
| $\alpha$ GFP-RSP02RA | +    | +       | +     | +       |
| 4F3-RSP02RA          | +    | +       | +     | +       |

(kDa)

20 —  
15 —  
10 —

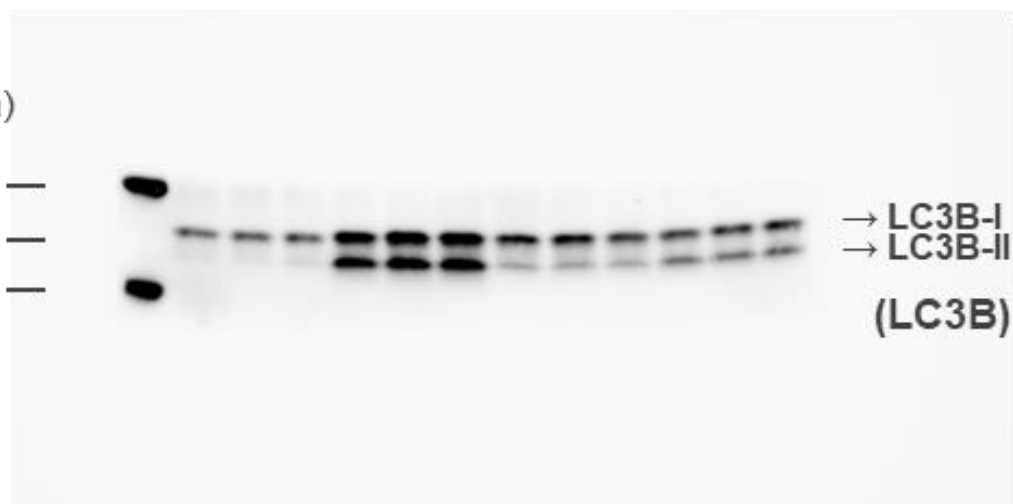

Supplement: Figure 6—source data 4. [file elife-93908-fig6-data4.zip › Figure 6D anti-LC3B with 4F3-RSPO2RA treatment Labelled Raw Data.pdf]

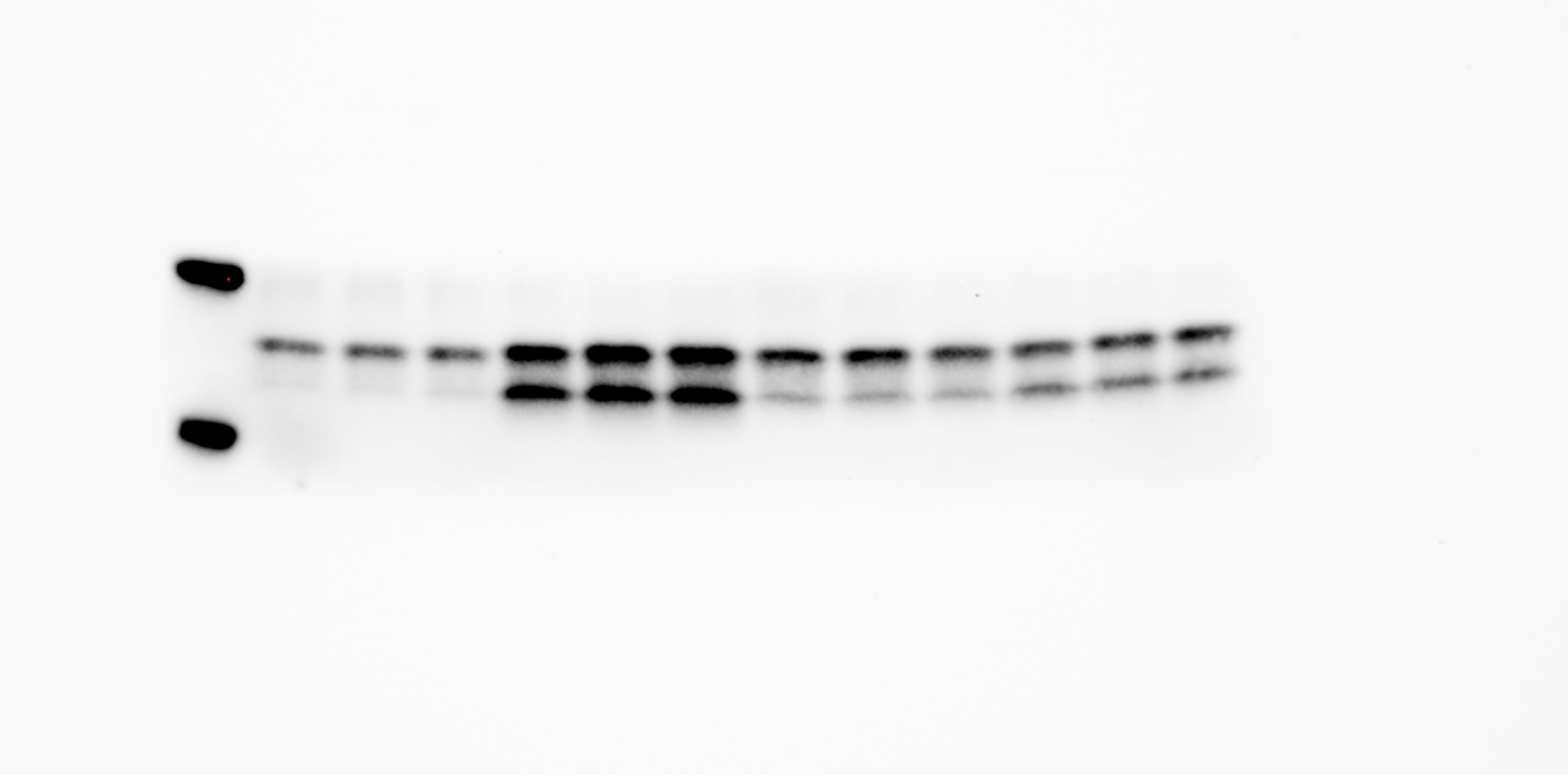

Supplement: Figure 6—source data 4. [file elife-93908-fig6-data4.zip › Figure 6D anti-LC3B with 4F3-RSPO2RA treatment Raw Data.tif]

|                      | DMSO | Baf. A1 | MG132 | TAK-243 |
|----------------------|------|---------|-------|---------|
| $\alpha$ GFP-RSPO2RA | +    | +       | +     | +       |
| 8G8-RSPO2RA          | +    | +       | +     | +       |

(kDa)

20 —  
15 —  
10 —

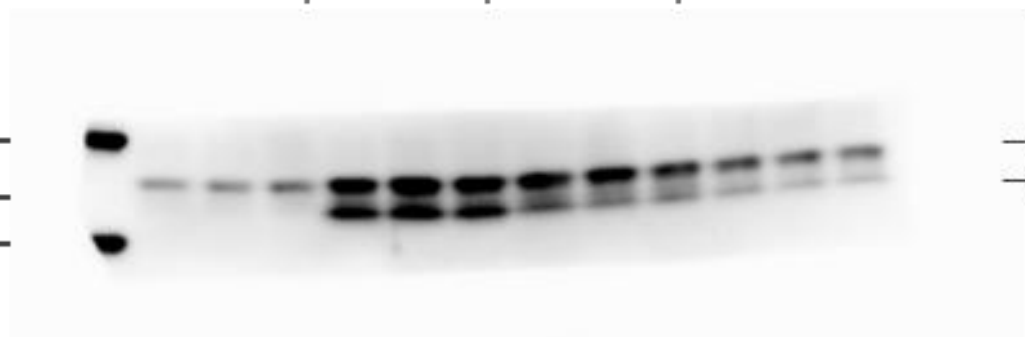

→ LC3B-I  
→ LC3B-II  
(LC3B)

Supplement: Figure 6—source data 4. [file elife-93908-fig6-data4.zip › Figure 6D anti-LC3B with 8G8-RSPO2RA treatment Labelled Raw Data.pdf]

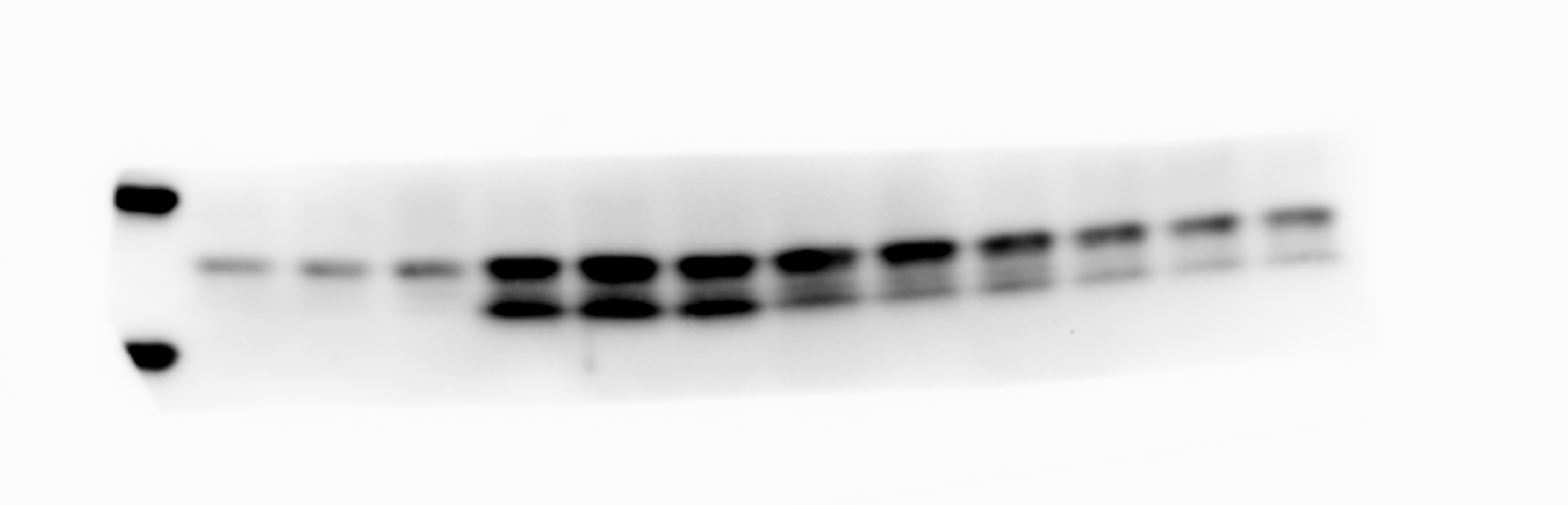

Supplement: Figure 6—source data 4. [file elife-93908-fig6-data4.zip › Figure 6D anti-LC3B with 8G8-RSPO2RA treatment Raw Data.tif]

|                      | DMSO | Baf. A1 | MG132 | TAK-243 |
|----------------------|------|---------|-------|---------|
| $\alpha$ GFP-RSPO2RA | +    | +       | +     | +       |
| 8M24-RSPO2RA         | +    | +       | +     | +       |

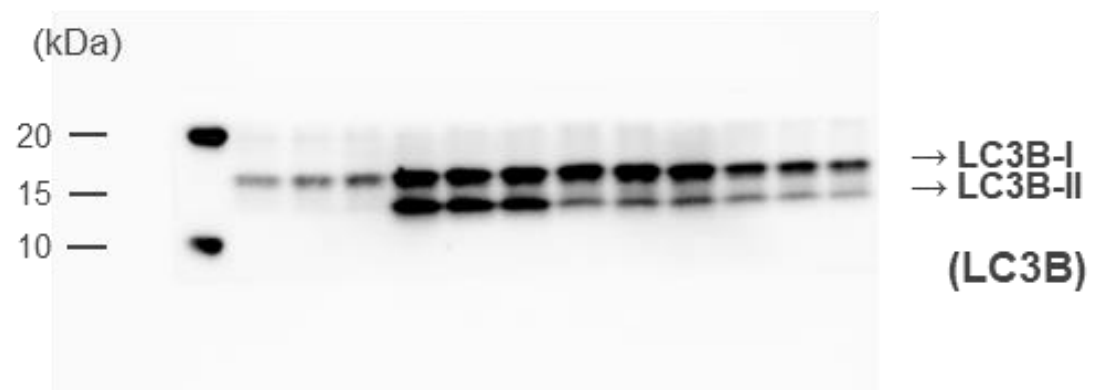

Supplement: Figure 6—source data 4. [file elife-93908-fig6-data4.zip › Figure 6D anti-LC3B with 8M24-RSPO2RA treatment Labelled Raw Data.pdf]

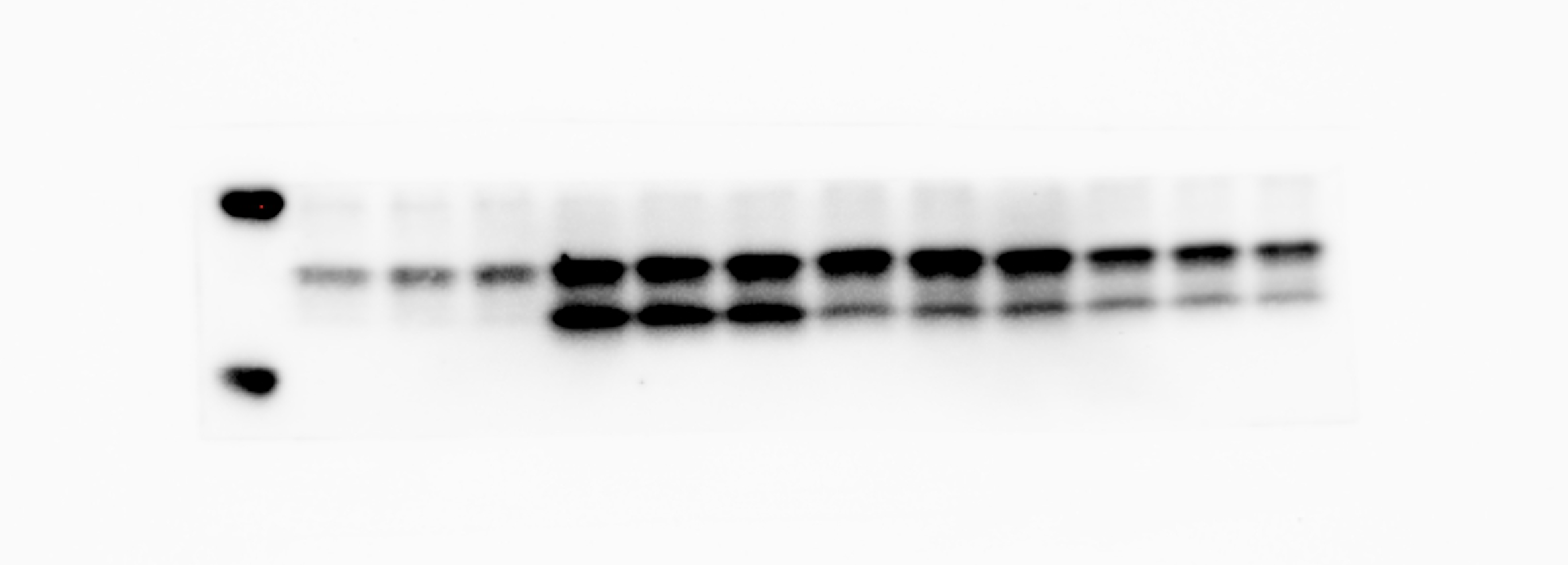

Supplement: Figure 6—source data 4. [file elife-93908-fig6-data4.zip › Figure 6D anti-LC3B with 8M24-RSPO2RA treatment Raw Data.tif]

|                      | DMSO | Baf. A1 | MG132 | TAK-243 |
|----------------------|------|---------|-------|---------|
| $\alpha$ GFP-RSPO2RA | +    | +       | +     | +       |
| 4F3-RSPO2RA          | +    | +       | +     | +       |

(kDa)

250 —  
150 —  
100 —  
75 —  
50 —  
37 —

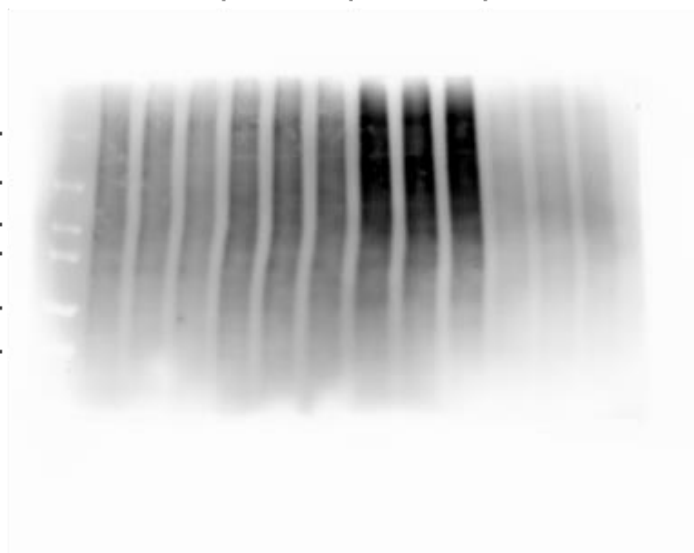

**Ubiquitin**

Supplement: Figure 6—source data 4. [file elife-93908-fig6-data4.zip › Figure 6D anti-Ubiquitin with 4F3-RSPO2RA treatment Labelled Raw Data.pdf]

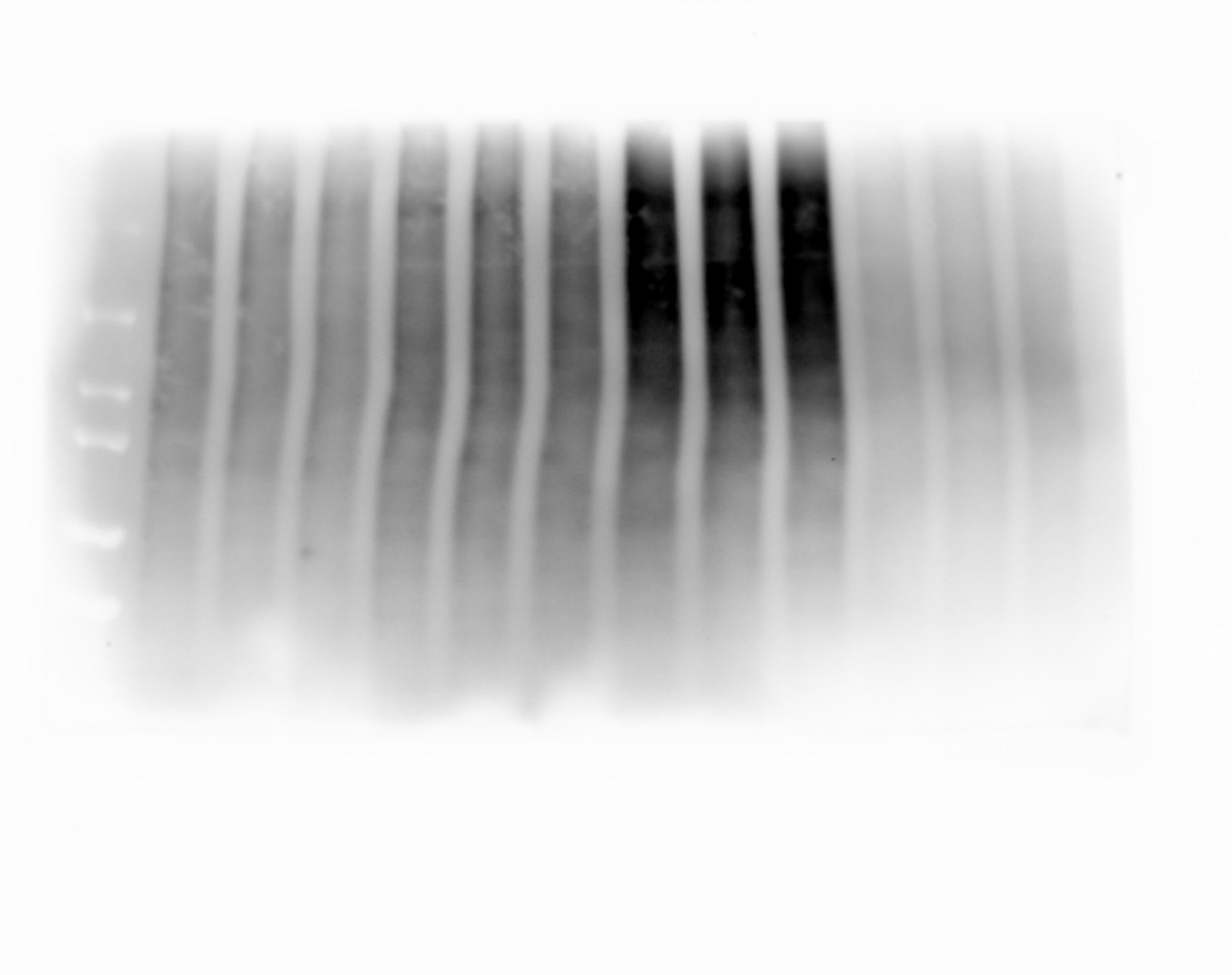

Supplement: Figure 6—source data 4. [file elife-93908-fig6-data4.zip › Figure 6D anti-Ubiquitin with 4F3-RSPO2RA treatment Raw Data.tif]

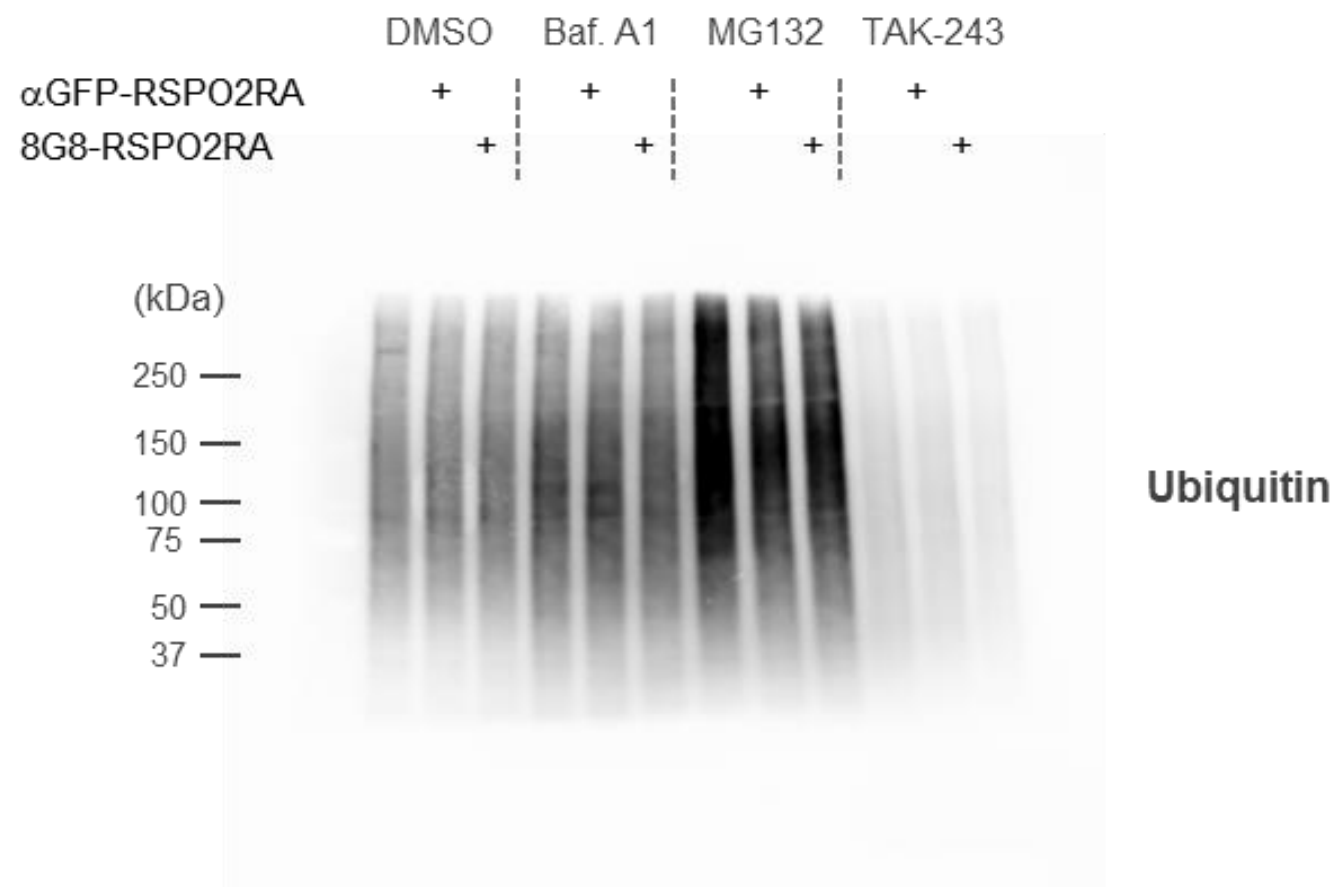

Supplement: Figure 6—source data 4. [file elife-93908-fig6-data4.zip › Figure 6D anti-Ubiquitin with 8G8-RSPO2RA treatment Labelled Raw Data.pdf]

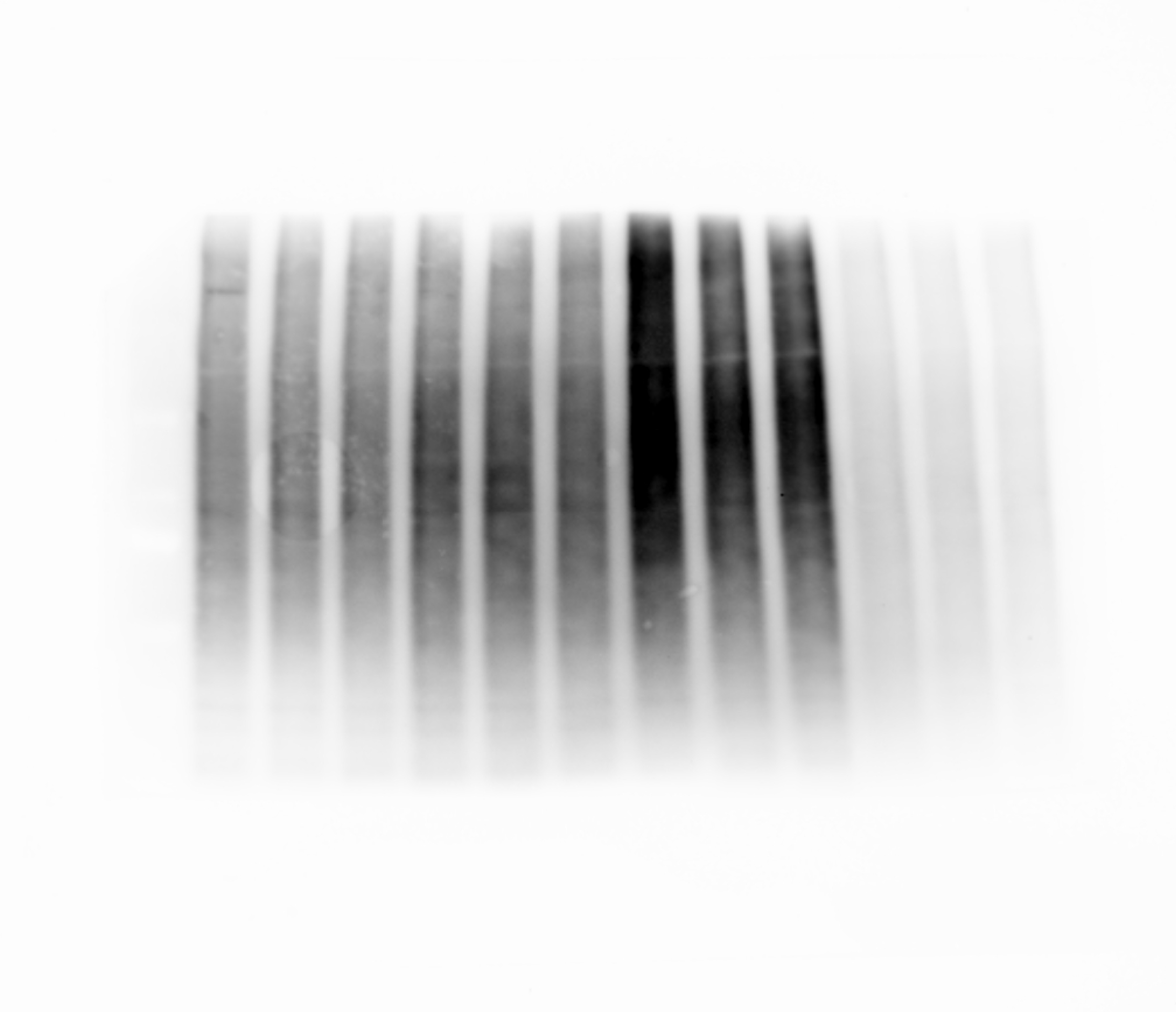

Supplement: Figure 6—source data 4. [file elife-93908-fig6-data4.zip › Figure 6D anti-Ubiquitin with 8G8-RSPO2RA treatment Raw Data.tif]

|                      | DMSO | Baf. A1 | MG132 | TAK-243 |
|----------------------|------|---------|-------|---------|
| $\alpha$ GFP-RSPO2RA | +    | +       | +     | +       |
| 8M24-RSPO2RA         | +    | +       | +     | +       |

(kDa)

250 —  
150 —  
100 —  
75 —  
50 —  
37 —

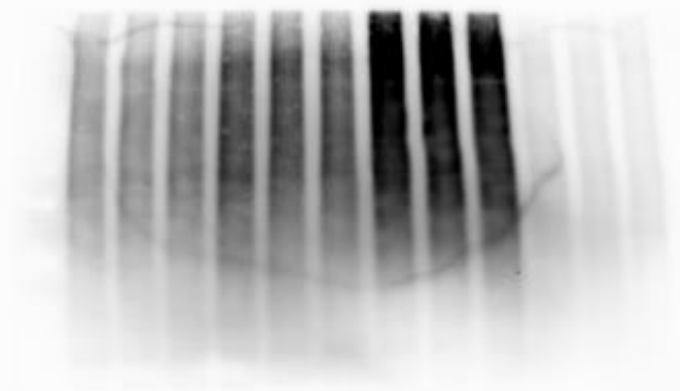

**Ubiquitin**

Supplement: Figure 6—source data 4. [file elife-93908-fig6-data4.zip › Figure 6D anti-Ubiquitin with 8M24-RSPO2RA treatment Labelled Raw Data.pdf]

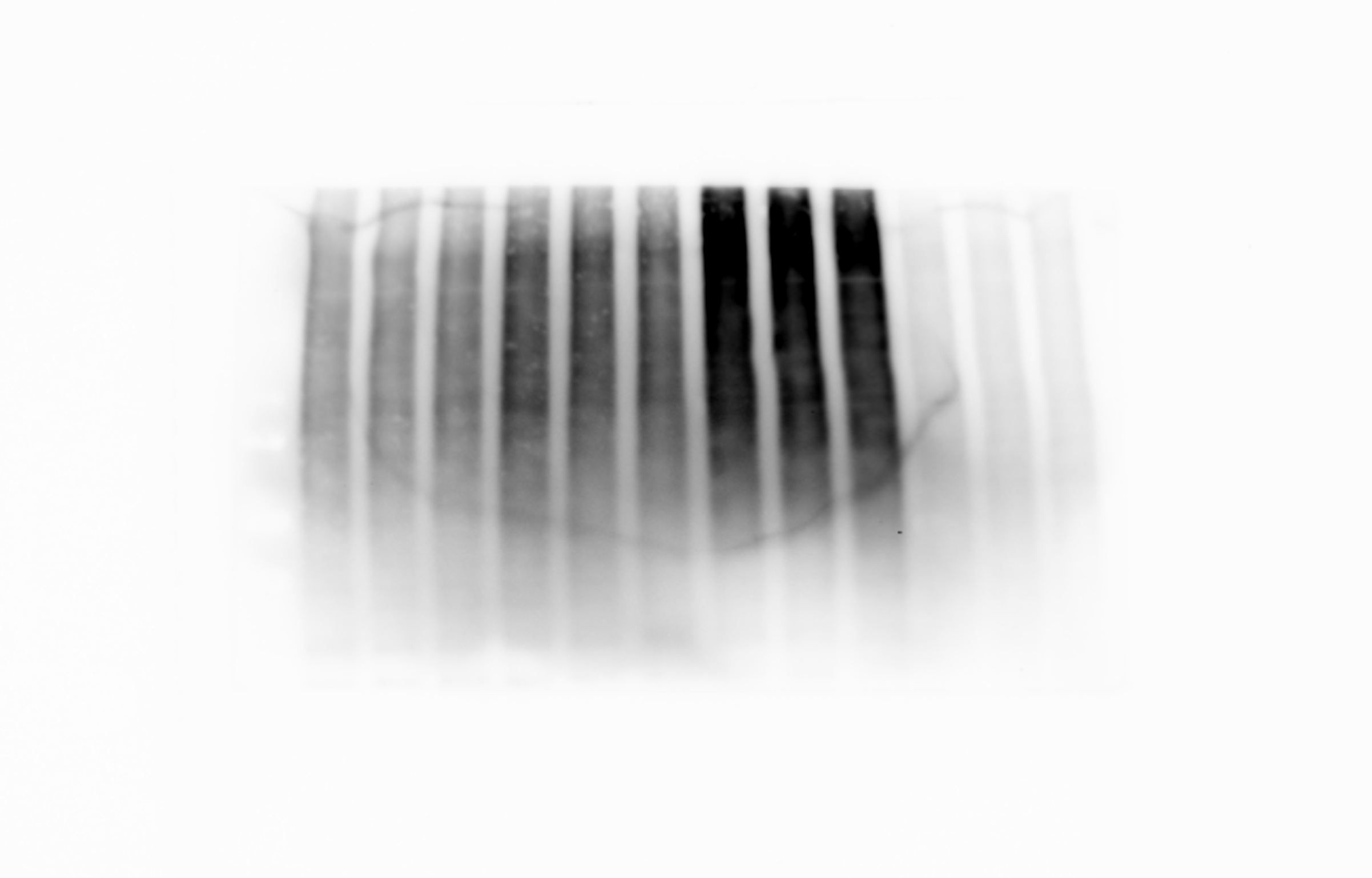

Supplement: Figure 6—source data 4. [file elife-93908-fig6-data4.zip › Figure 6D anti-Ubiquitin with 8M24-RSPO2RA treatment Raw Data.tif]

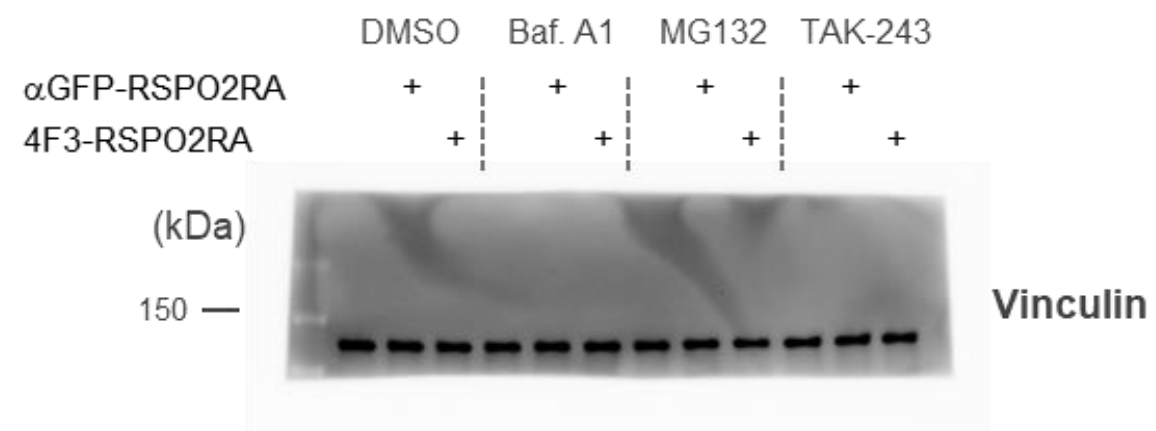

Supplement: Figure 6—source data 4. [file elife-93908-fig6-data4.zip › Figure 6D anti-Vinculin with 4F3-RSPO2RA treatment Labelled Raw Data.pdf]

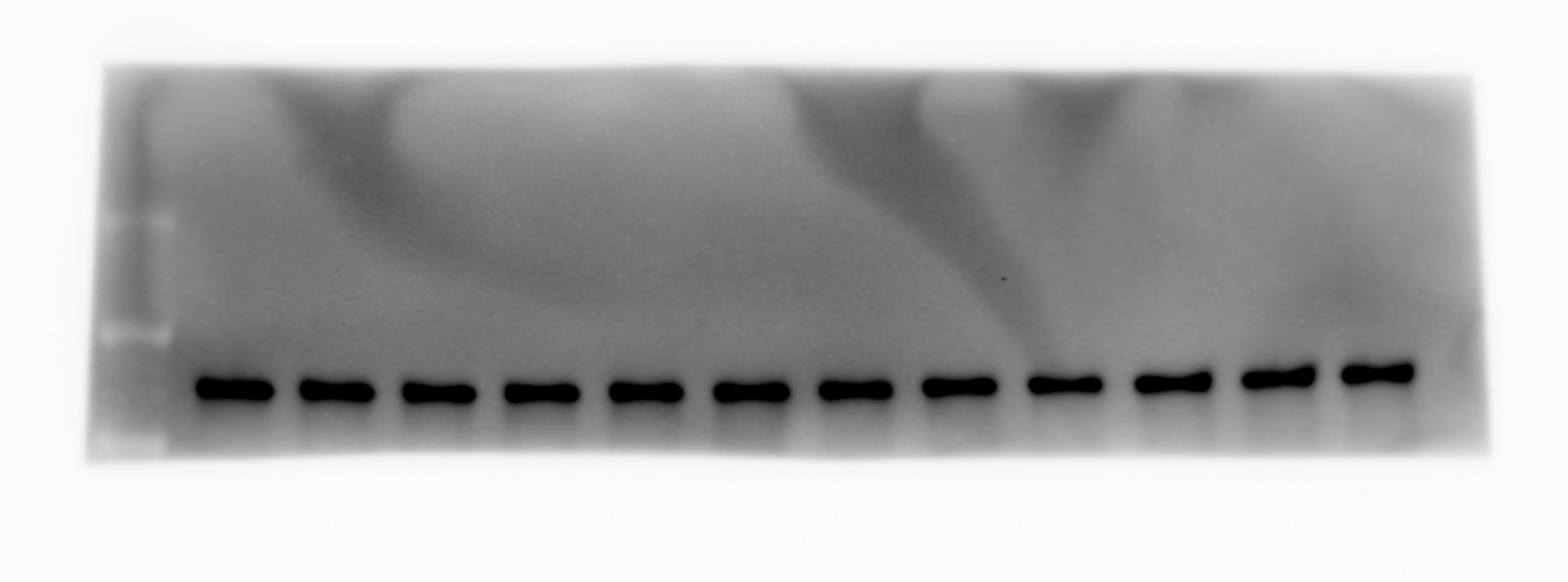

Supplement: Figure 6—source data 4. [file elife-93908-fig6-data4.zip › Figure 6D anti-Vinculin with 4F3-RSPO2RA treatment Raw Data.tif]

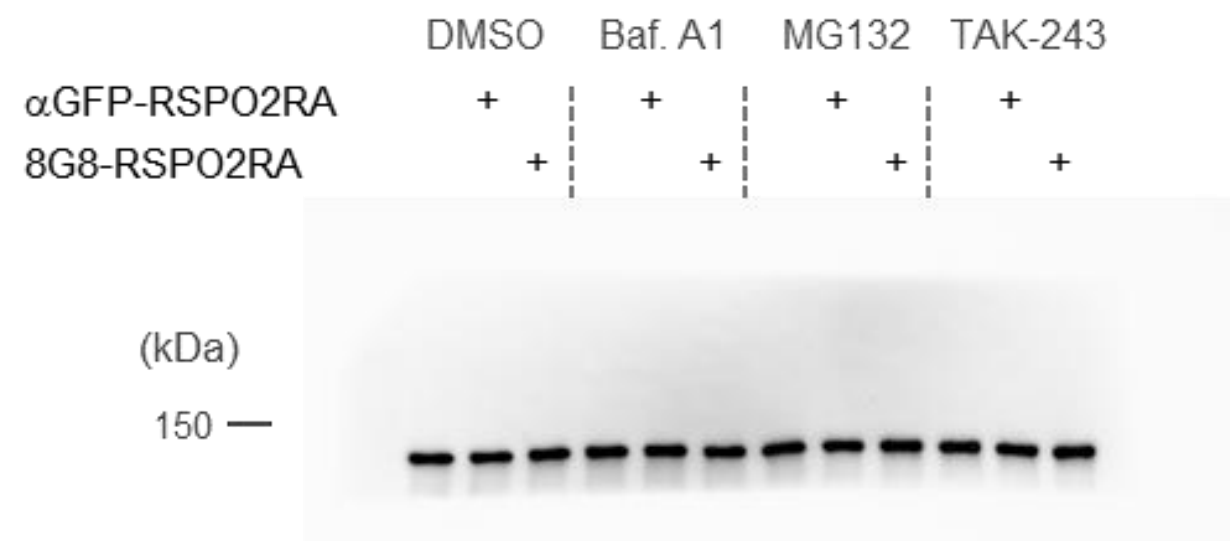

Supplement: Figure 6—source data 4. [file elife-93908-fig6-data4.zip › Figure 6D anti-Vinculin with 8G8-RSPO2RA treatment Labelled Raw Data.pdf]

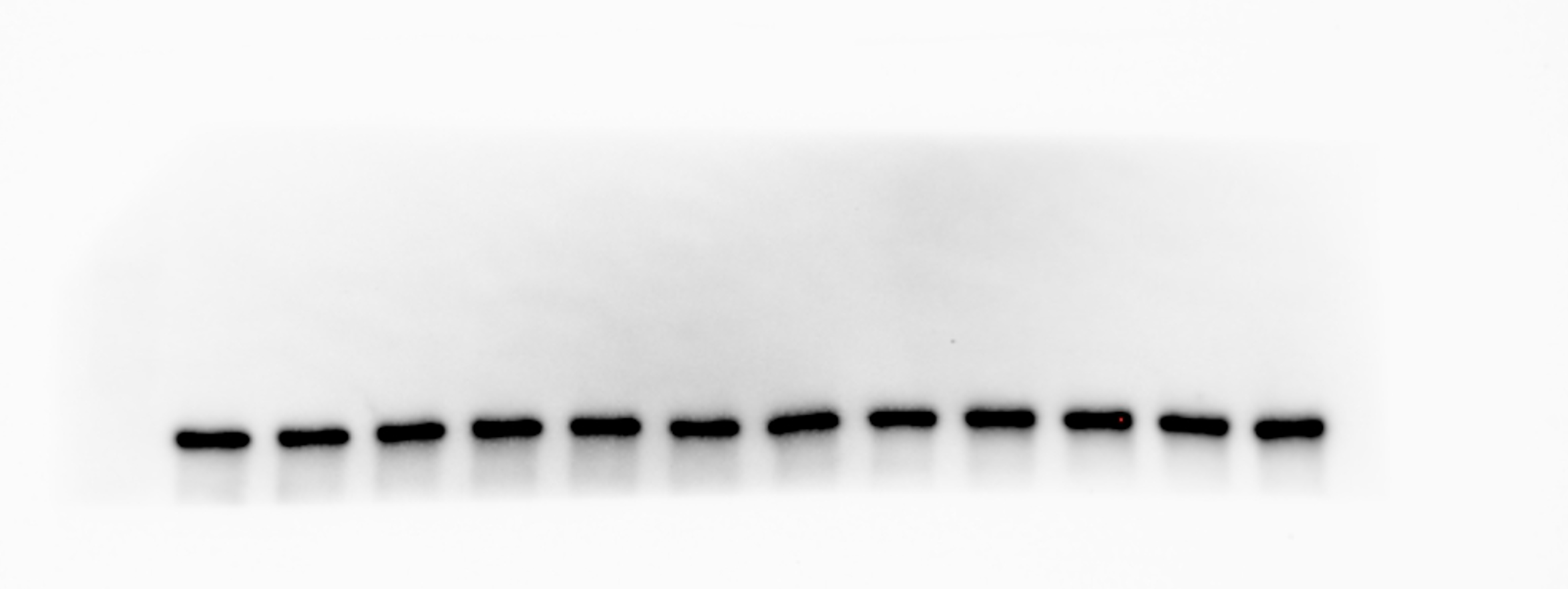

Supplement: Figure 6—source data 4. [file elife-93908-fig6-data4.zip › Figure 6D anti-Vinculin with 8G8-RSPO2RA treatment Raw Data.tif]

|                      | DMSO | Baf. A1 | MG132 | TAK-243 |
|----------------------|------|---------|-------|---------|
| $\alpha$ GFP-RSP02RA | +    | +       | +     | +       |
| 8M24-RSP02RA         | +    | +       | +     | +       |

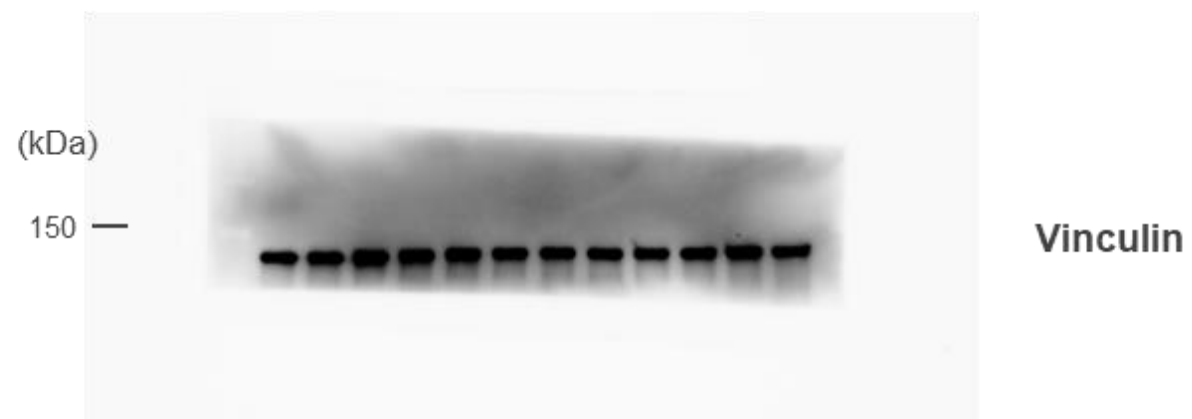

Supplement: Figure 6—source data 4. [file elife-93908-fig6-data4.zip › Figure 6D anti-Vinculin with 8M24-RSPO2RA treatment Labelled Raw Data.pdf]

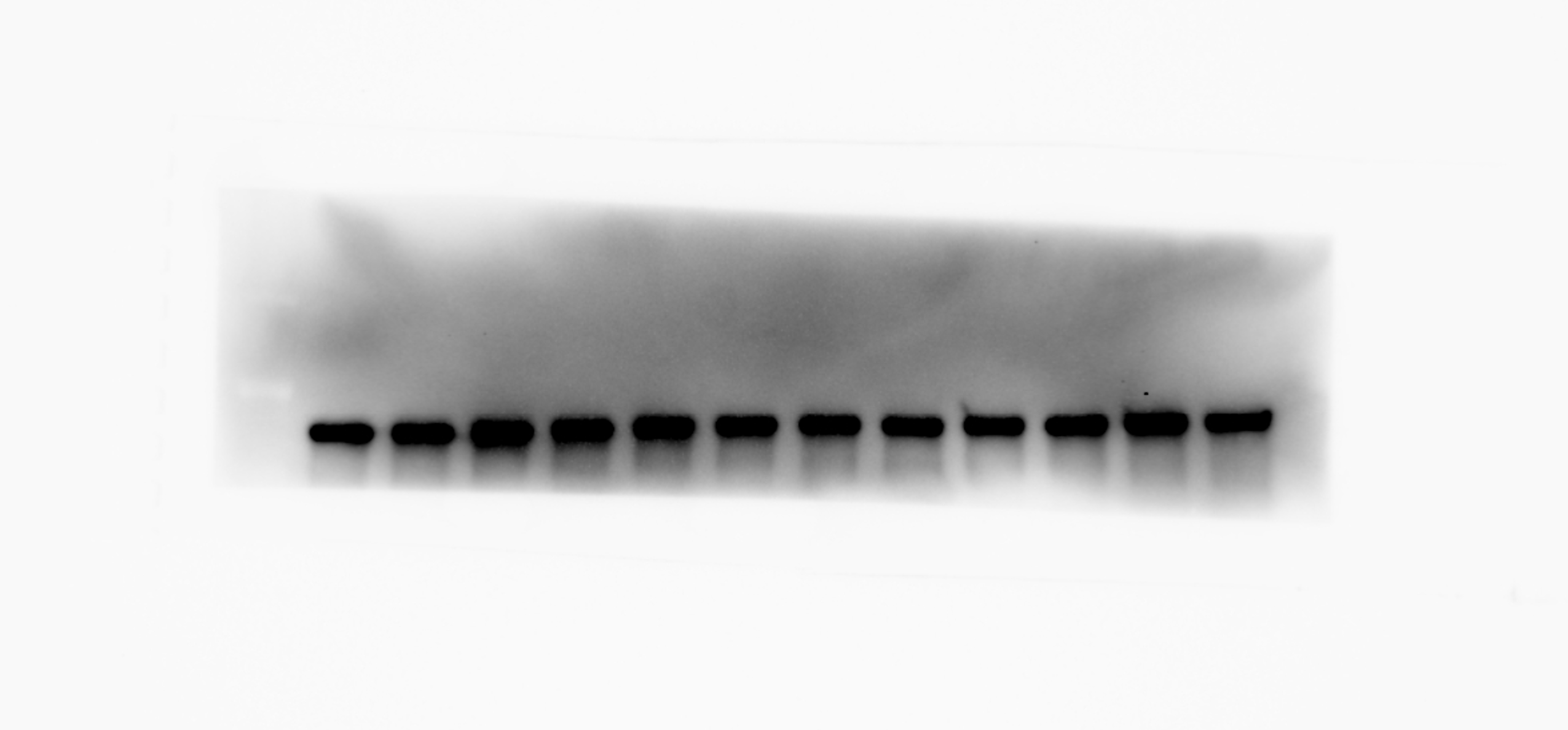

Supplement: Figure 6—source data 4. [file elife-93908-fig6-data4.zip › Figure 6D anti-Vinculin with 8M24-RSPO2RA treatment Raw Data.tif]

# 4F3-RSPO2RA

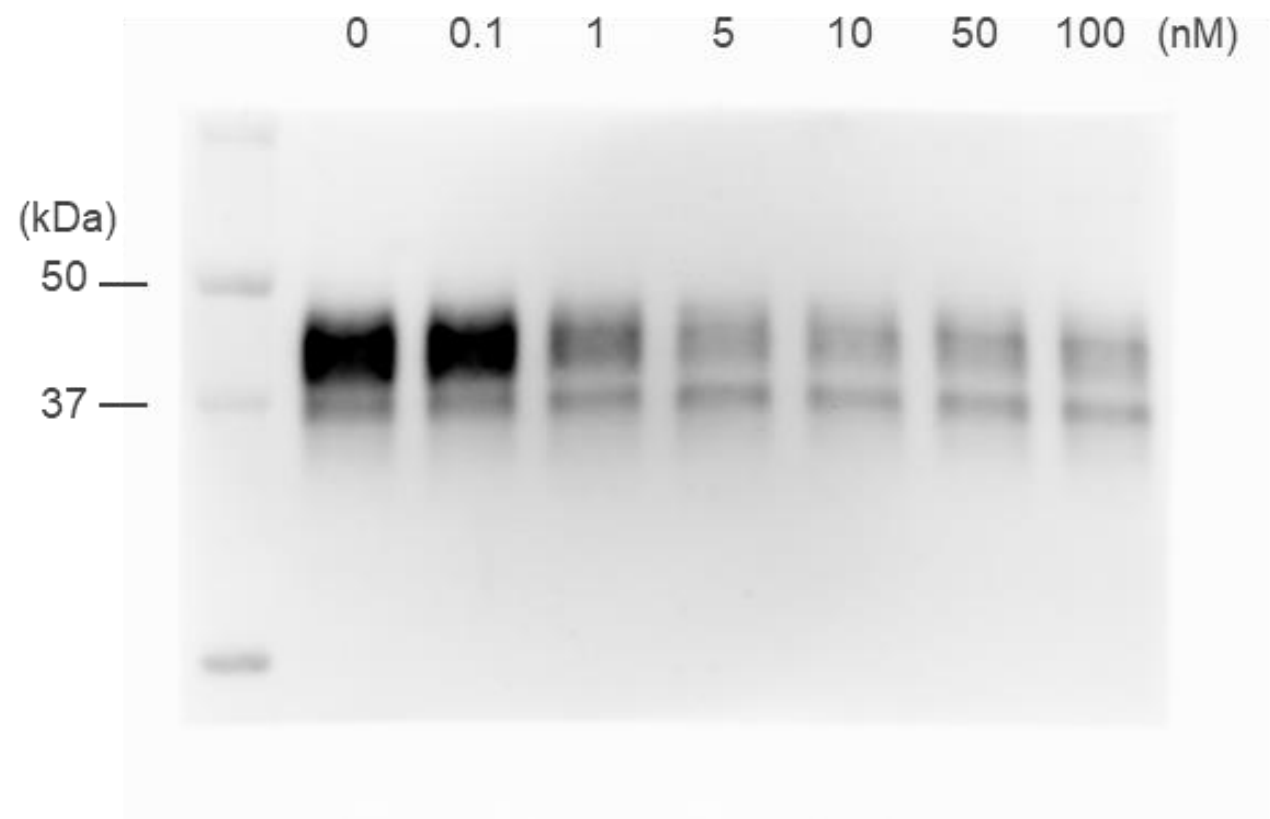

Supplement: Figure 6—figure supplement 1—source data 1. [file elife-93908-fig6-figsupp1-data1.zip › Figure 6S1A anti-ASGR1 with 4F3-RSPO2RA treatment Labelled Raw Data.pdf]

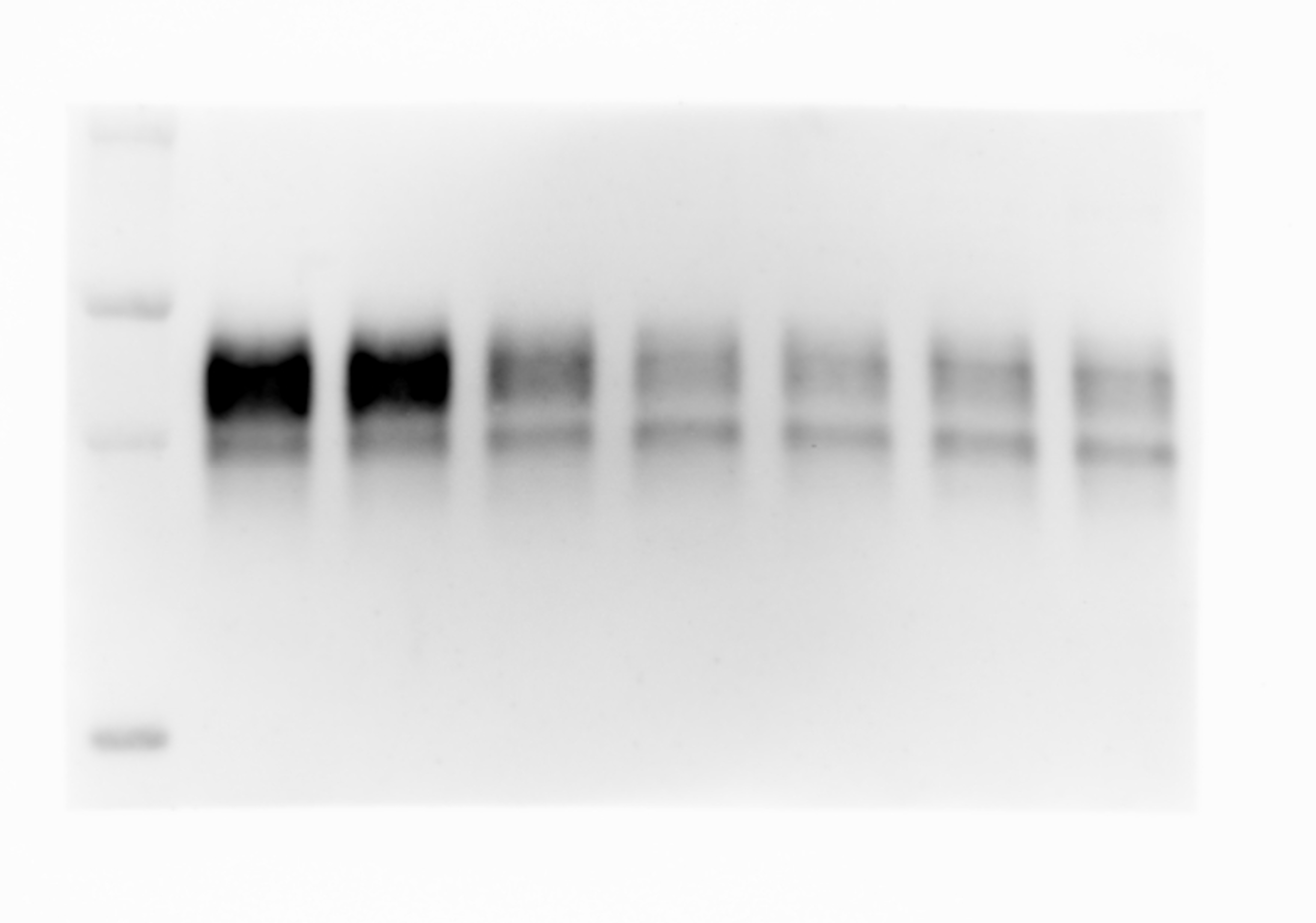

Supplement: Figure 6—figure supplement 1—source data 1. [file elife-93908-fig6-figsupp1-data1.zip › Figure 6S1A anti-ASGR1 with 4F3-RSPO2RA treatment Raw Data.tif]

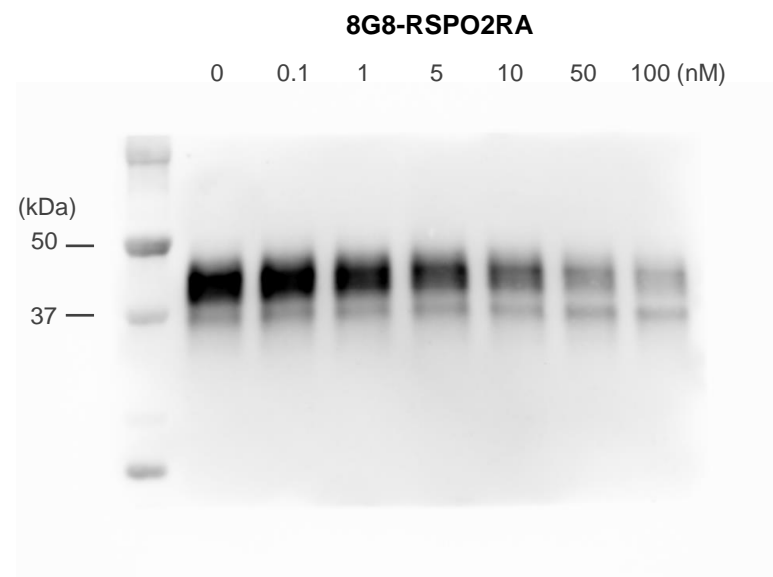

Supplement: Figure 6—figure supplement 1—source data 1. [file elife-93908-fig6-figsupp1-data1.zip › Figure 6S1A anti-ASGR1 with 8G8-RSPO2RA treatment Labelled Raw Data.pdf]

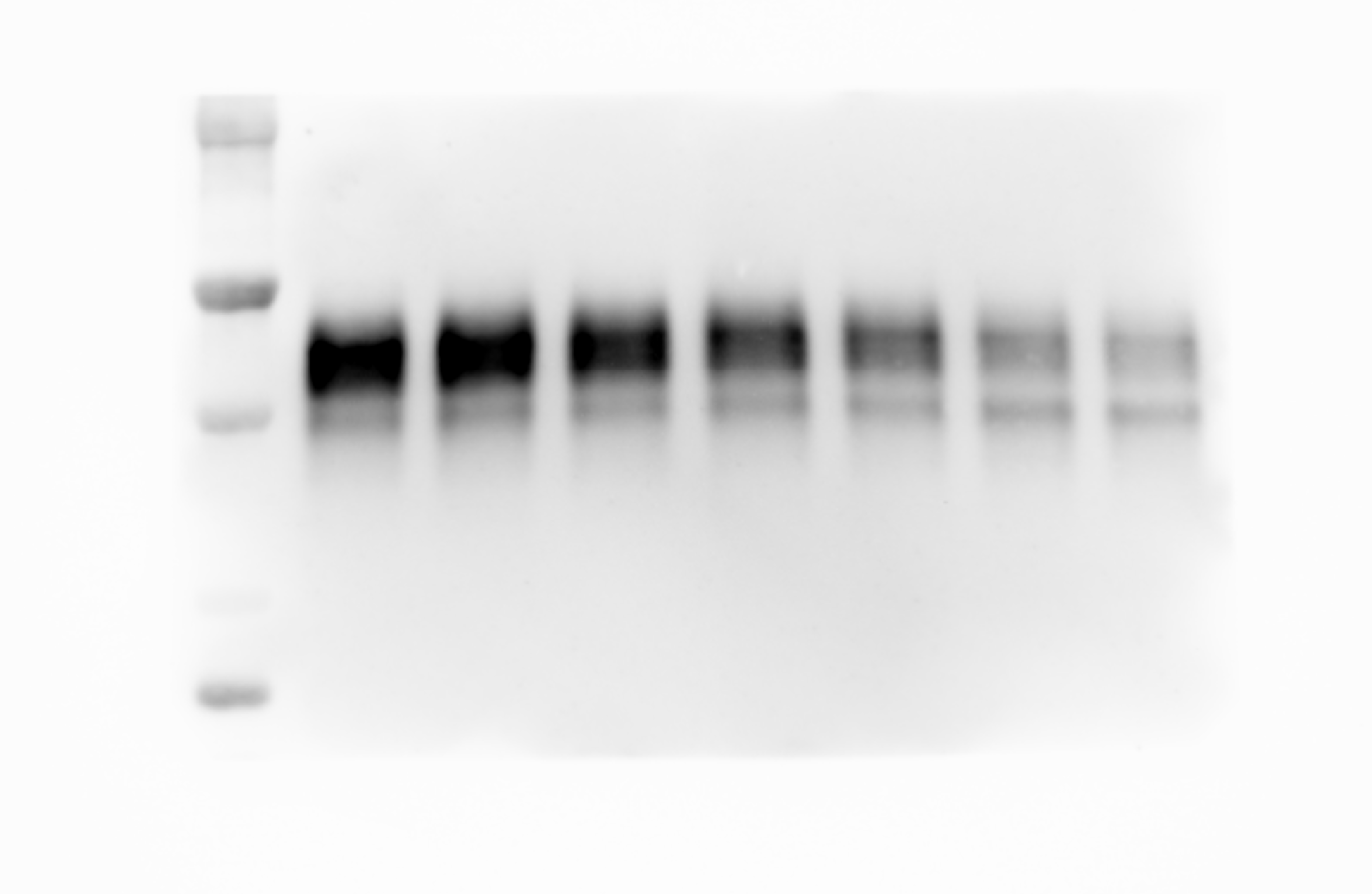

Supplement: Figure 6—figure supplement 1—source data 1. [file elife-93908-fig6-figsupp1-data1.zip › Figure 6S1A anti-ASGR1 with 8G8-RSPO2RA treatment Raw Data.tif]

# 8M24-RSPO2RA

0 0.1 1 5 10 50 100 (nM)

(kDa)

50 —

37 —

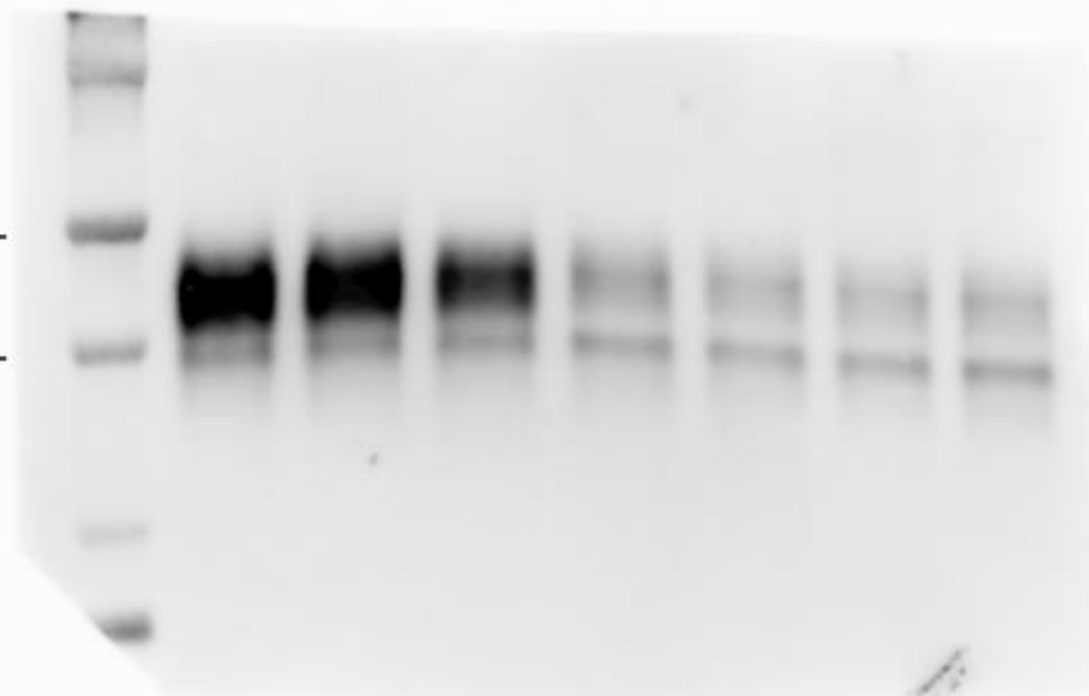

Supplement: Figure 6—figure supplement 1—source data 1. [file elife-93908-fig6-figsupp1-data1.zip › Figure 6S1A anti-ASGR1 with 8M24-RSPO2RA treatment Labelled Raw Data.pdf]

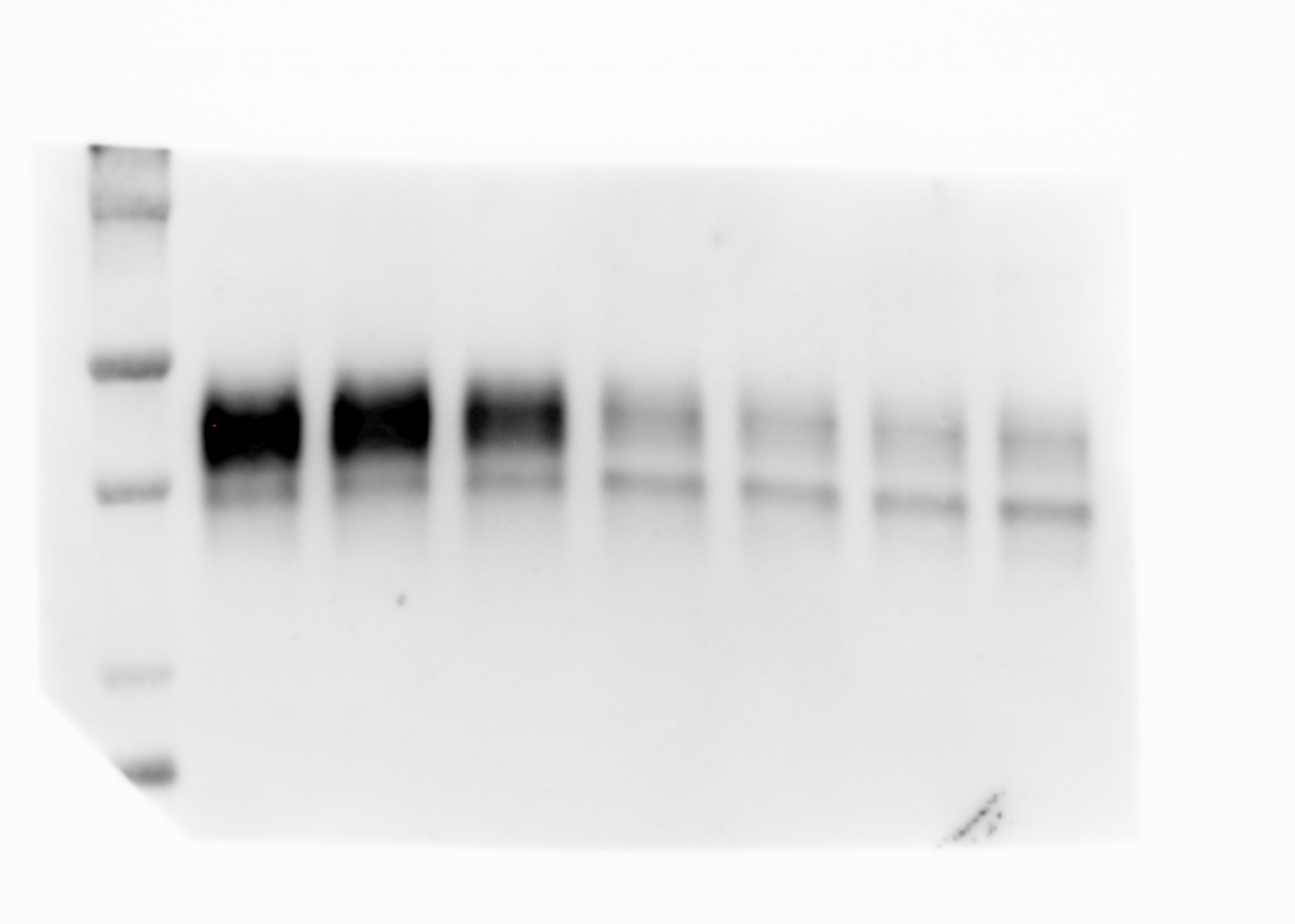

Supplement: Figure 6—figure supplement 1—source data 1. [file elife-93908-fig6-figsupp1-data1.zip › Figure 6S1A anti-ASGR1 with 8M24-RSPO2RA treatment Raw Data.tif]

# 4F3-RSPO2RA

0 0.1 1 5 10 50 100 (nM)

(kDa)

150—

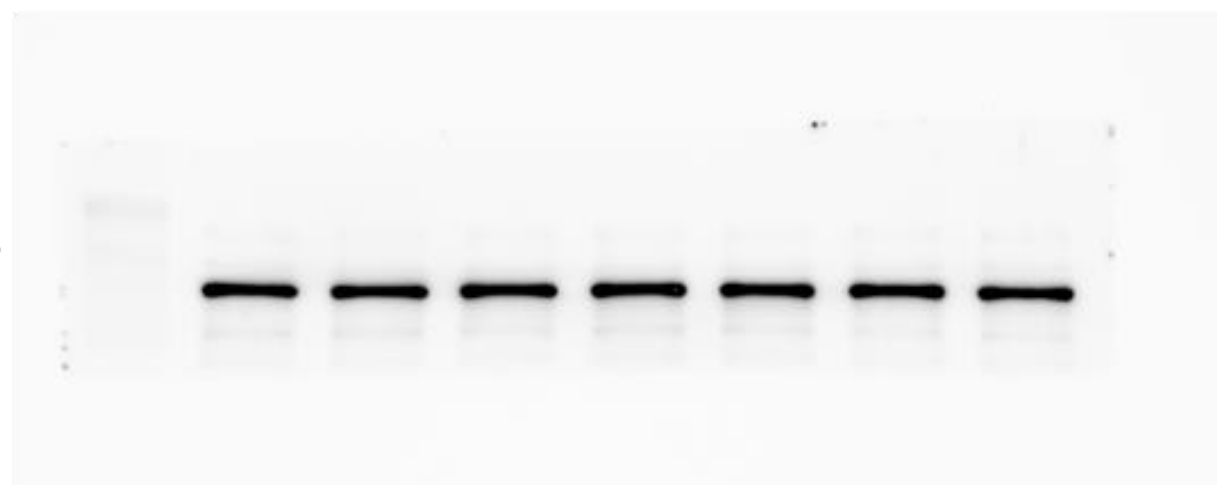

Supplement: Figure 6—figure supplement 1—source data 1. [file elife-93908-fig6-figsupp1-data1.zip › Figure 6S1A anti-Vinculin with 4F3-RSPO2RA treatment Labelled Raw Data.pdf]

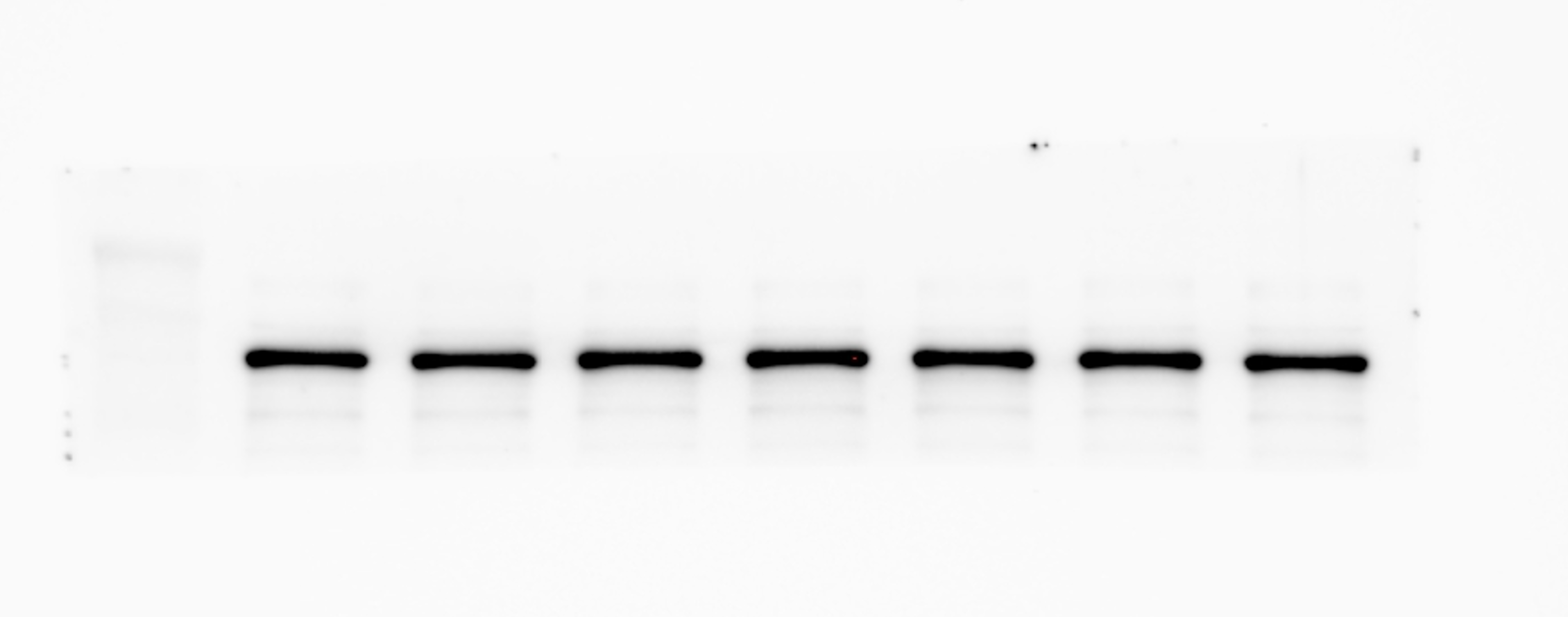

Supplement: Figure 6—figure supplement 1—source data 1. [file elife-93908-fig6-figsupp1-data1.zip › Figure 6S1A anti-Vinculin with 4F3-RSPO2RA treatment Raw Data.tif]

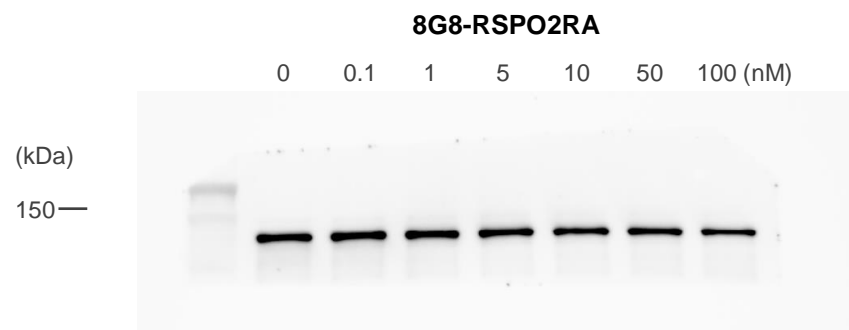

Supplement: Figure 6—figure supplement 1—source data 1. [file elife-93908-fig6-figsupp1-data1.zip › Figure 6S1A anti-Vinculin with 8G8-RSPO2RA treatment Labelled Raw Data.pdf]

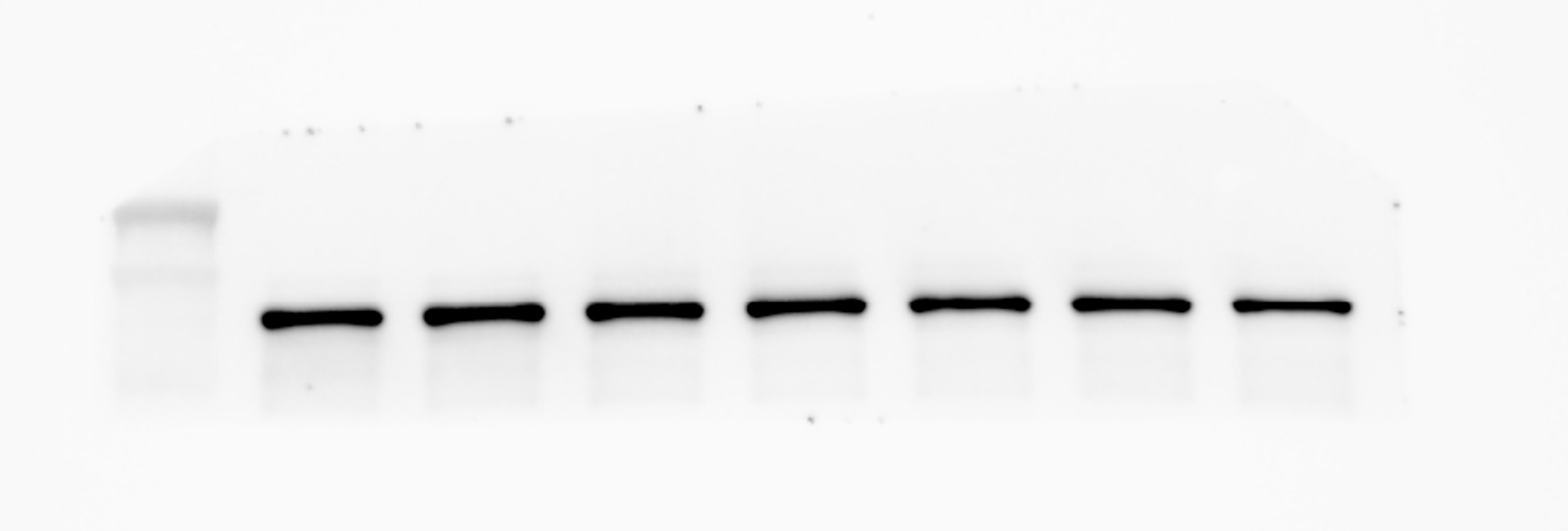

Supplement: Figure 6—figure supplement 1—source data 1. [file elife-93908-fig6-figsupp1-data1.zip › Figure 6S1A anti-Vinculin with 8G8-RSPO2RA treatment Raw Data.tif]

### 8M24-RSPO2RA

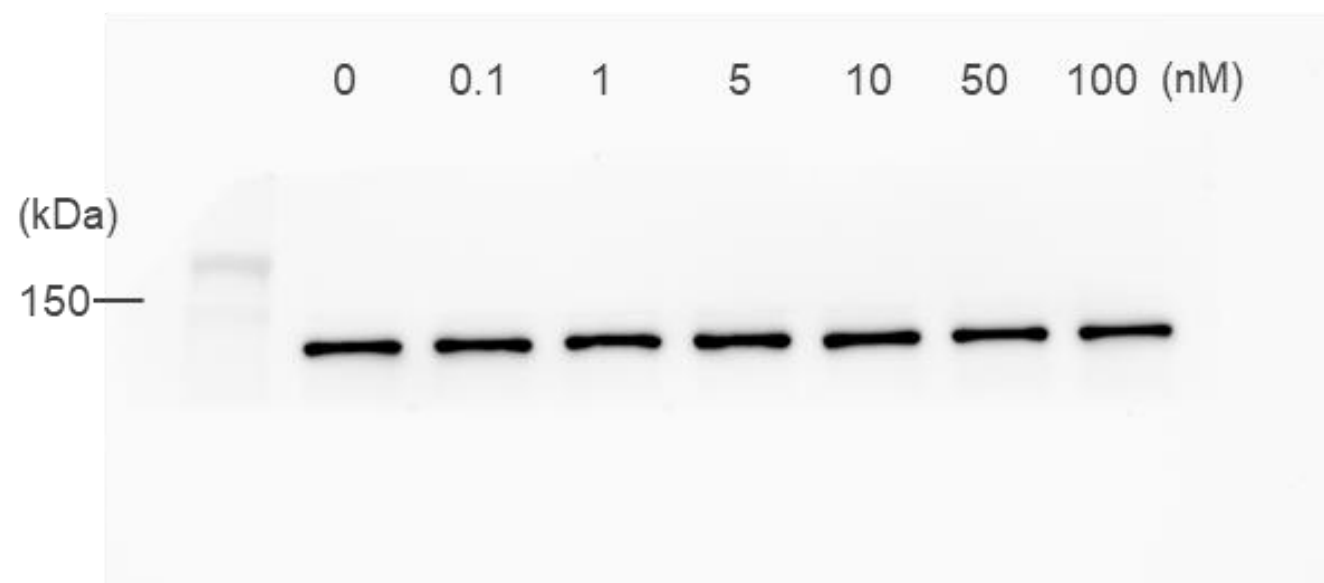

Supplement: Figure 6—figure supplement 1—source data 1. [file elife-93908-fig6-figsupp1-data1.zip › Figure 6S1A anti-Vinculin with 8M24-RSPO2RA treatment Labelled Raw Data.pdf]

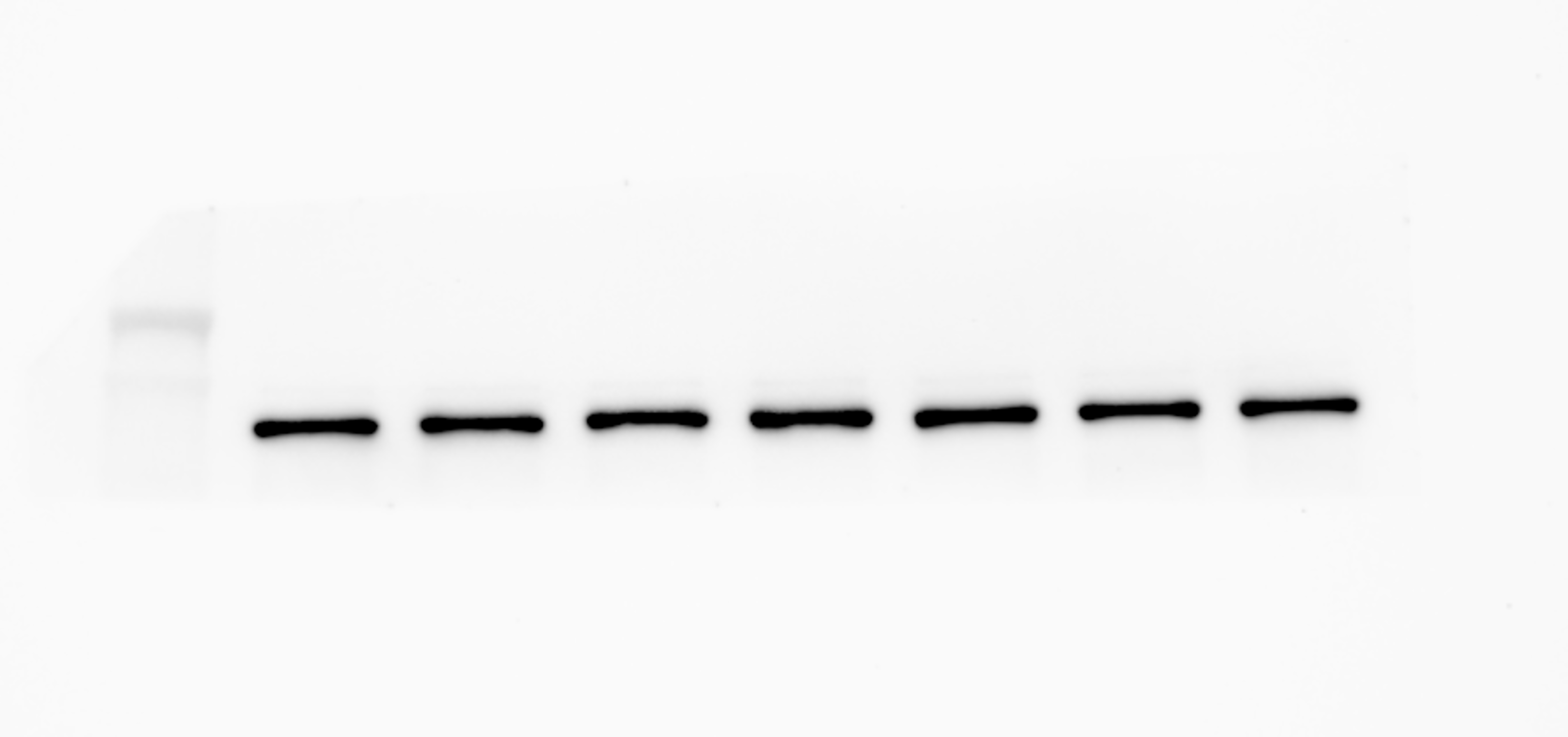

Supplement: Figure 6—figure supplement 1—source data 1. [file elife-93908-fig6-figsupp1-data1.zip › Figure 6S1A anti-Vinculin with 8M24-RSPO2RA treatment Raw Data.tif]

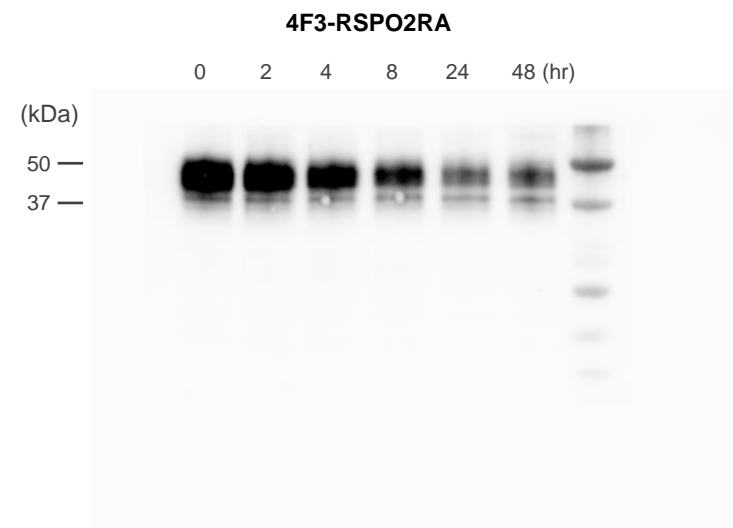

Supplement: Figure 6—figure supplement 1—source data 2. [file elife-93908-fig6-figsupp1-data2.zip › Figure 6S1B anti-ASGR1 with 4F3-RSPO2RA treatment Labelled Raw Data.pdf]

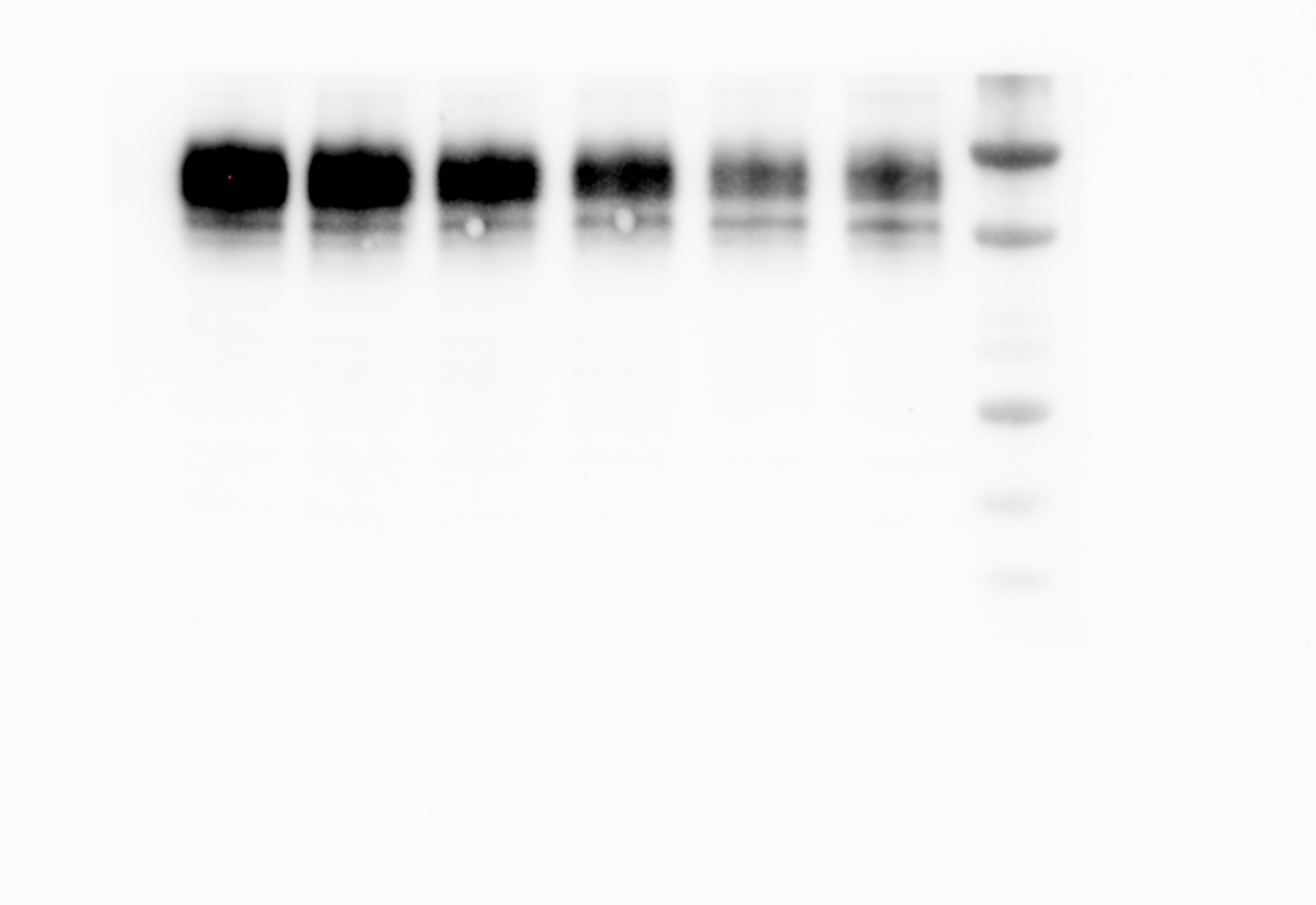

Supplement: Figure 6—figure supplement 1—source data 2. [file elife-93908-fig6-figsupp1-data2.zip › Figure 6S1B anti-ASGR1 with 4F3-RSPO2RA treatment Raw Data.tif]

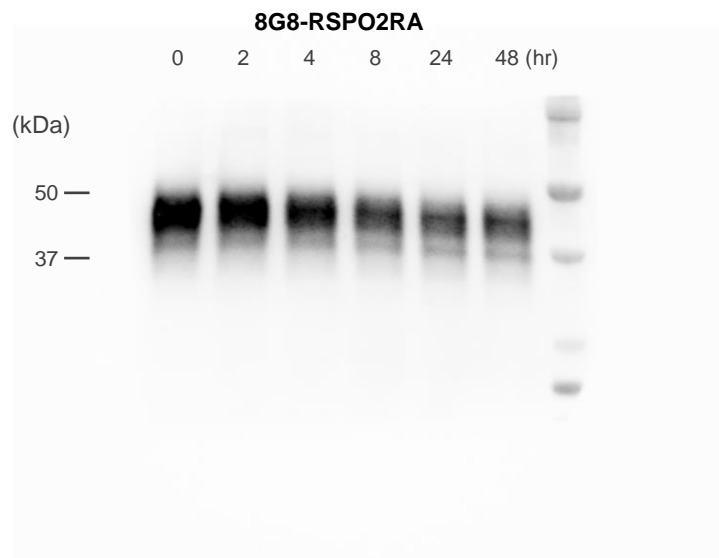

Supplement: Figure 6—figure supplement 1—source data 2. [file elife-93908-fig6-figsupp1-data2.zip › Figure 6S1B anti-ASGR1 with 8G8-RSPO2RA treatment Labelled Raw Data.pdf]

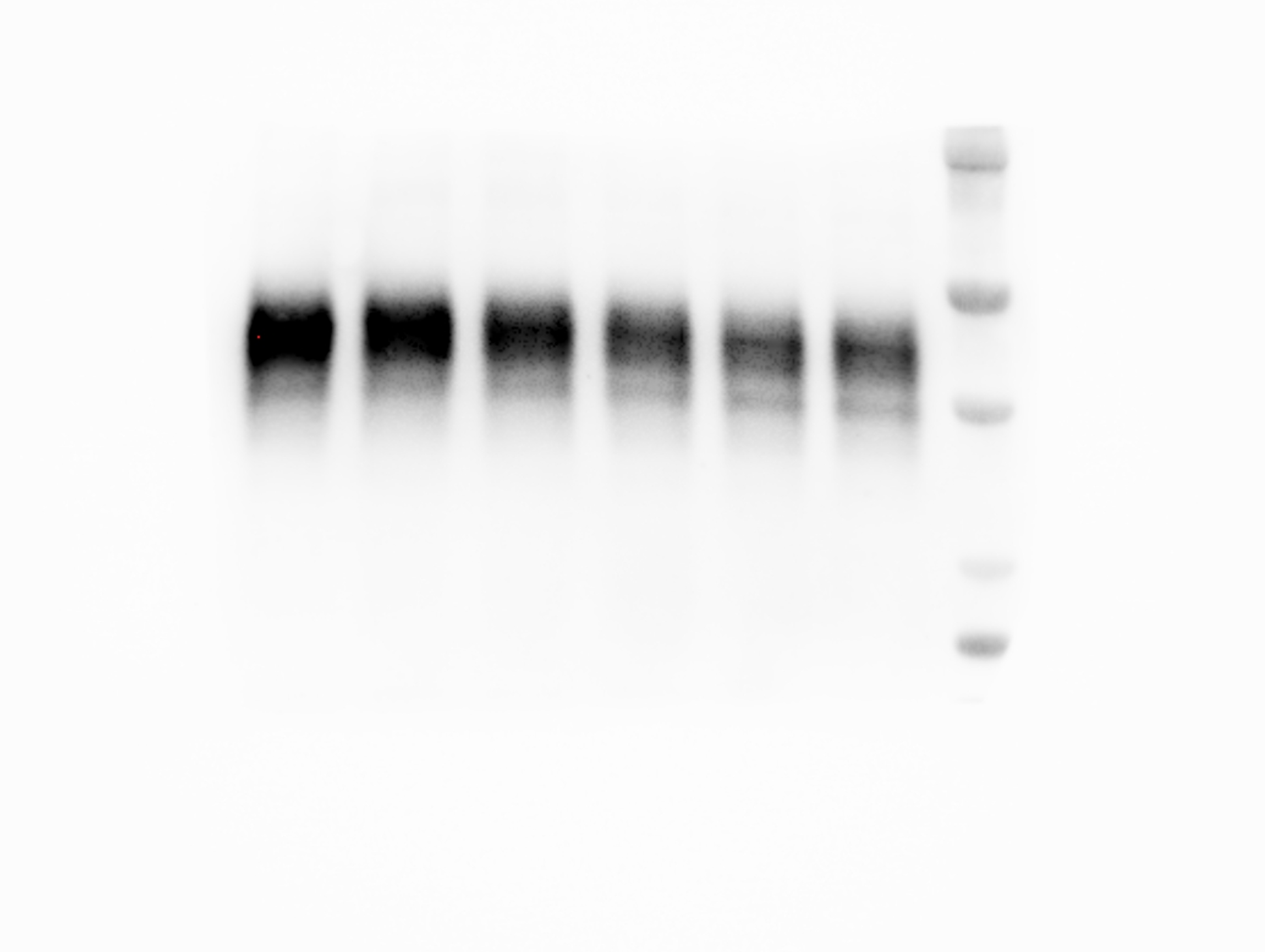

Supplement: Figure 6—figure supplement 1—source data 2. [file elife-93908-fig6-figsupp1-data2.zip › Figure 6S1B anti-ASGR1 with 8G8-RSPO2RA treatment Raw Data.tif]

# 8M24-RSPO2RA

0 2 4 8 24 48 (hr)

(kDa)

50 —

37 —

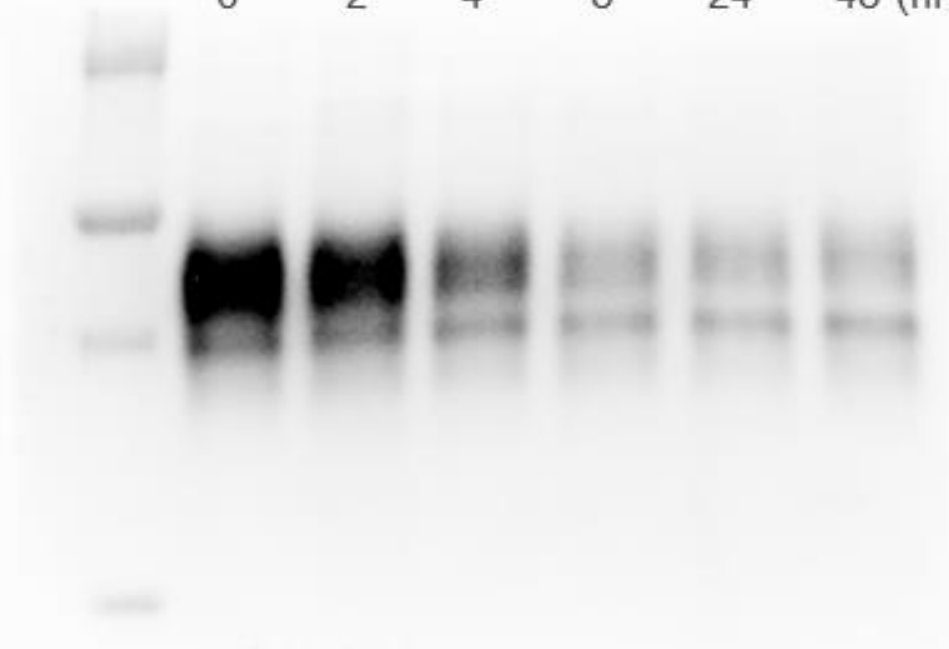

Supplement: Figure 6—figure supplement 1—source data 2. [file elife-93908-fig6-figsupp1-data2.zip › Figure 6S1B anti-ASGR1 with 8M24-RSPO2RA treatment Labelled Raw Data.pdf]

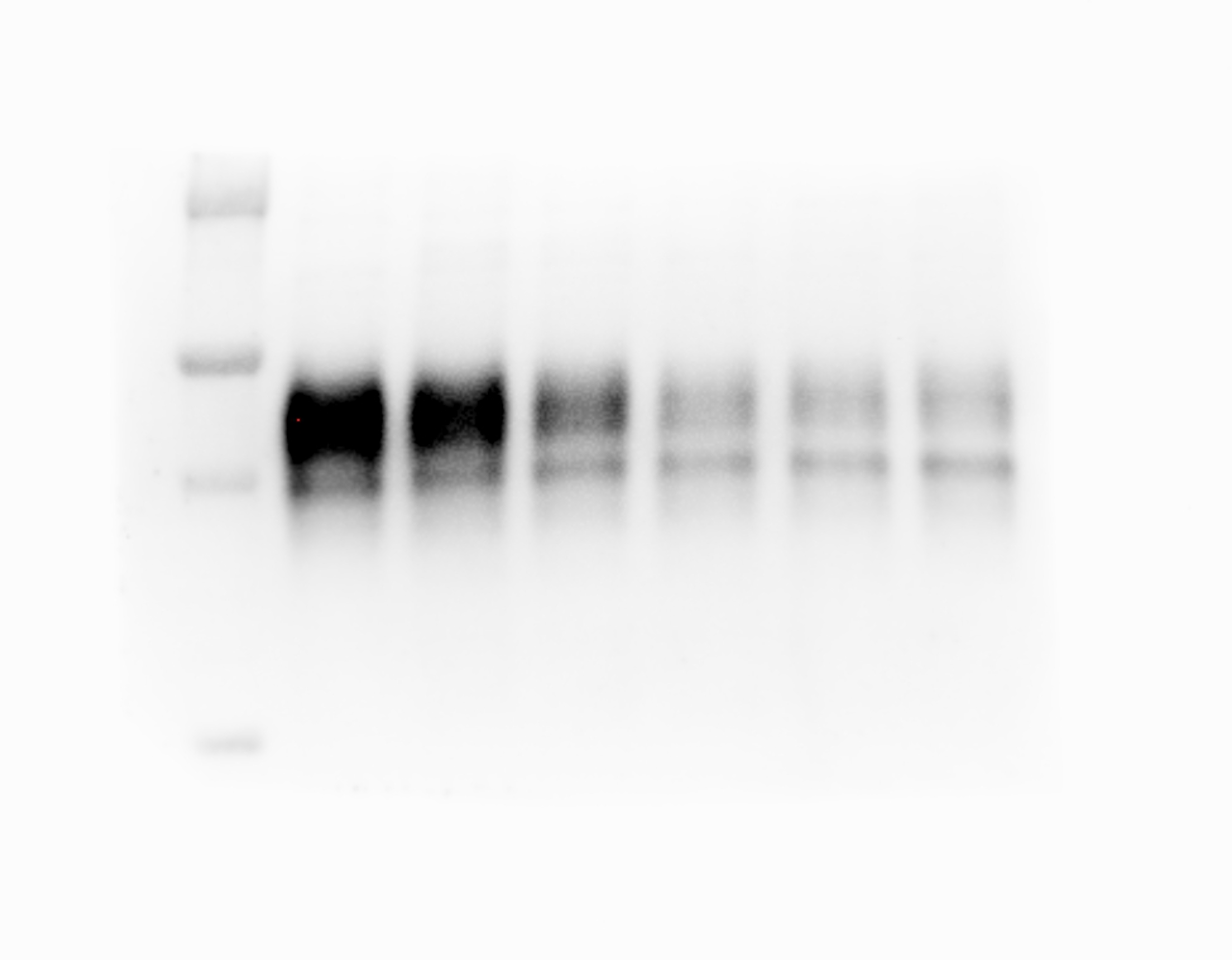

Supplement: Figure 6—figure supplement 1—source data 2. [file elife-93908-fig6-figsupp1-data2.zip › Figure 6S1B anti-ASGR1 with 8M24-RSPO2RA treatment Raw Data.tif]

# 4F3-RSPO2RA

0 2 4 8 24 48 (hr)

(kDa)

150 —

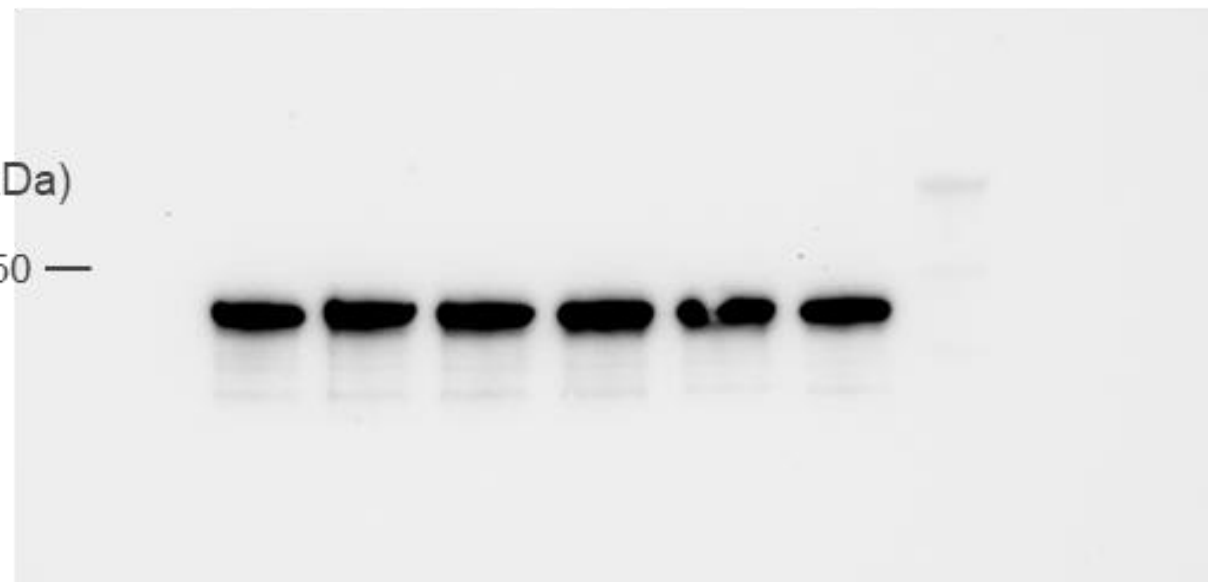

Supplement: Figure 6—figure supplement 1—source data 2. [file elife-93908-fig6-figsupp1-data2.zip › Figure 6S1B anti-Vinculin with 4F3-RSPO2RA treatment Labelled Raw Data.pdf]

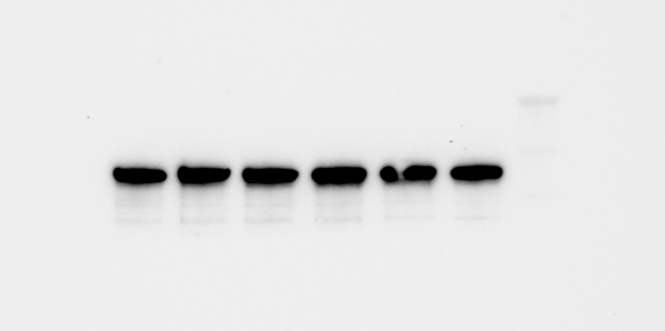

Supplement: Figure 6—figure supplement 1—source data 2. [file elife-93908-fig6-figsupp1-data2.zip › Figure 6S1B anti-vinculin with 4F3-RSPO2RA treatment Raw Data.tif]

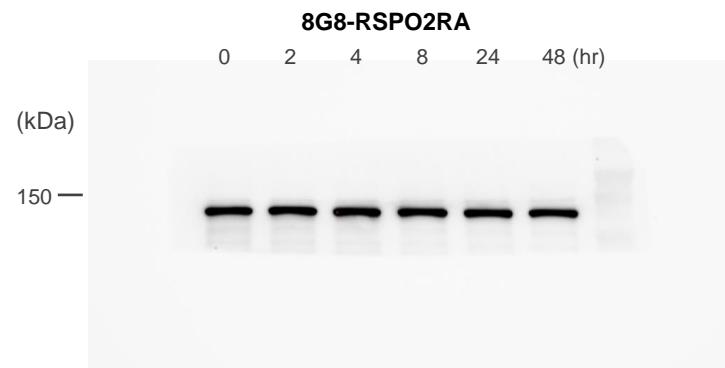

Supplement: Figure 6—figure supplement 1—source data 2. [file elife-93908-fig6-figsupp1-data2.zip › Figure 6S1B anti-Vinculin with 8G8-RSPO2RA treatment Labelled Raw Data.pdf]

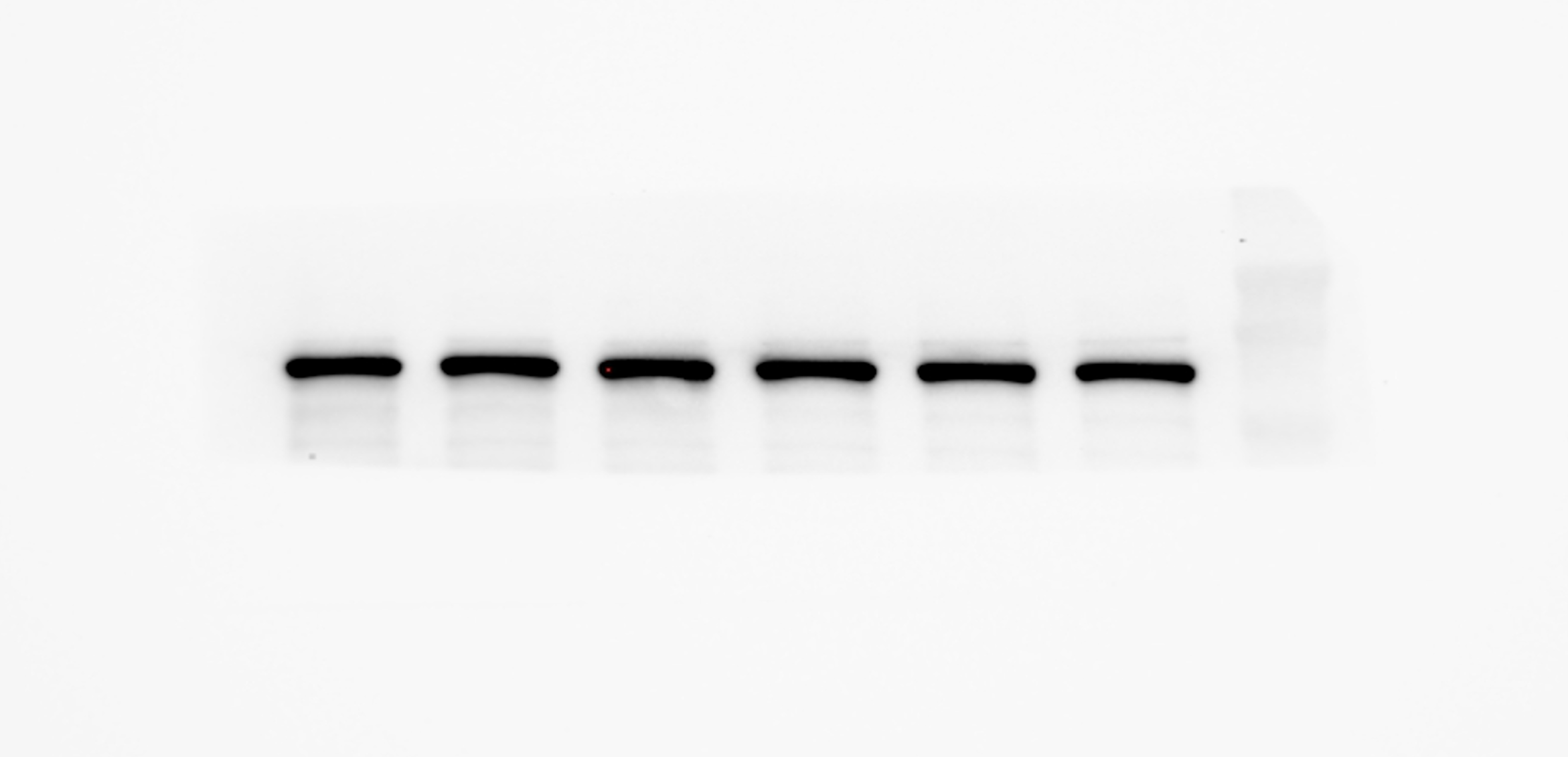

Supplement: Figure 6—figure supplement 1—source data 2. [file elife-93908-fig6-figsupp1-data2.zip › Figure 6S1B anti-vinculin with 8G8-RSPO2RA treatment Raw Data.tif]

### 8M24-RSPO2RA

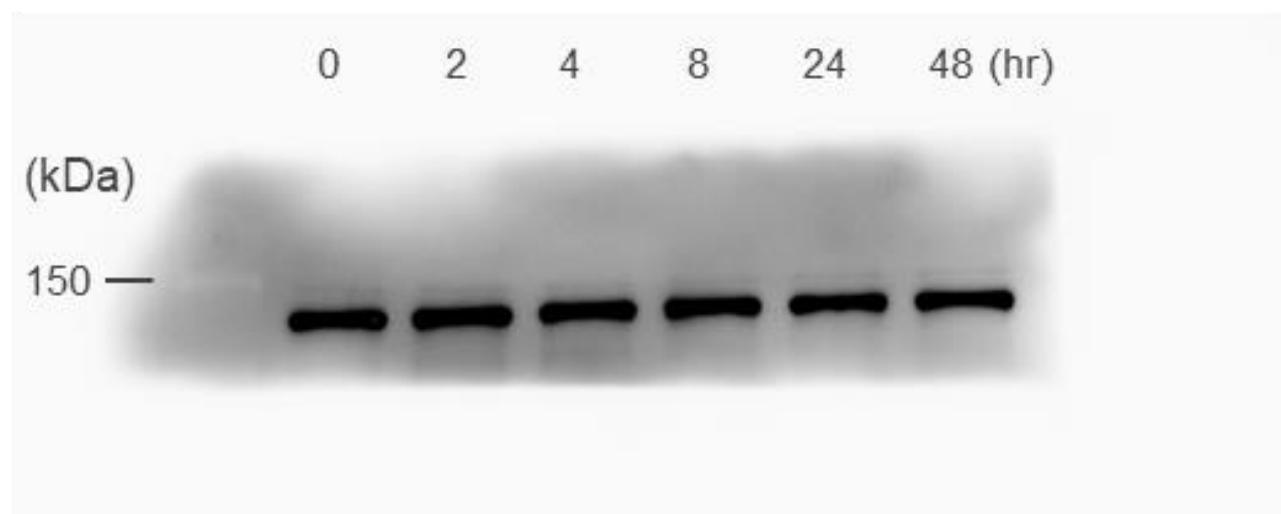

Supplement: Figure 6—figure supplement 1—source data 2. [file elife-93908-fig6-figsupp1-data2.zip › Figure 6S1B anti-Vinculin with 8M24-RSPO2RA treatment Labelled Raw Data.pdf]

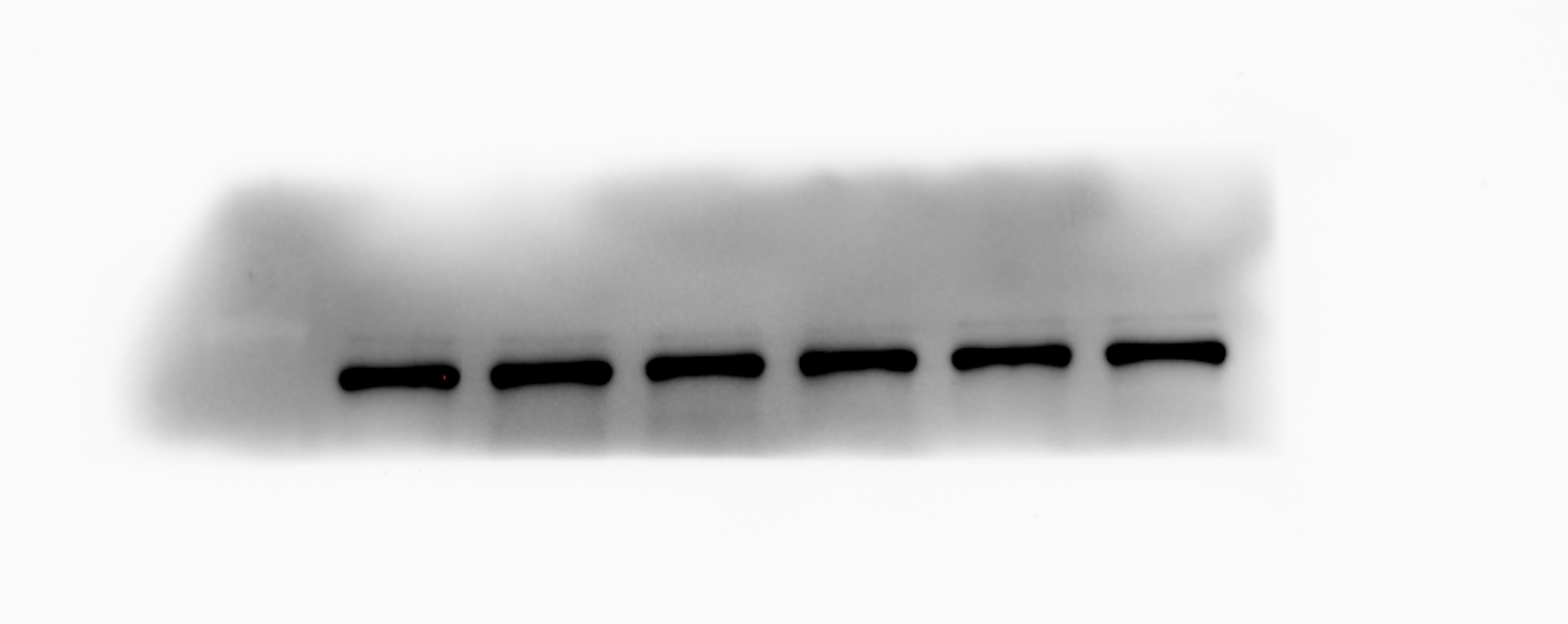

Supplement: Figure 6—figure supplement 1—source data 2. [file elife-93908-fig6-figsupp1-data2.zip › Figure 6S1B anti-vinculin with 8M24-RSPO2RA treatment Raw Data.tif]

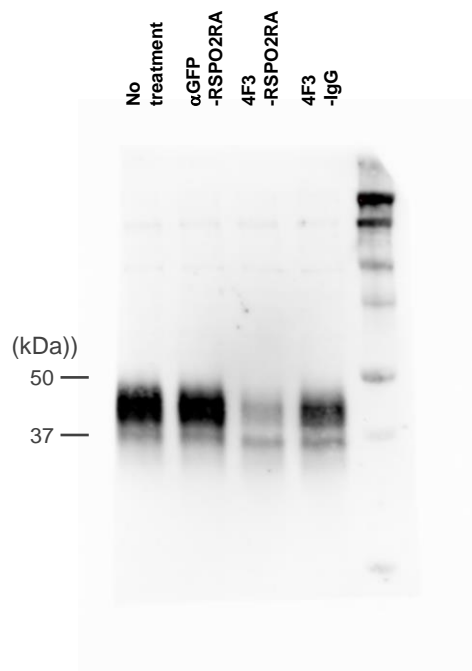

Supplement: Figure 6—figure supplement 1—source data 3. [file elife-93908-fig6-figsupp1-data3.zip › Figure 6S1C anti-ASGR1 with 4F3-RSPO2RA or IgG treatment Labelled Raw Data.pdf]

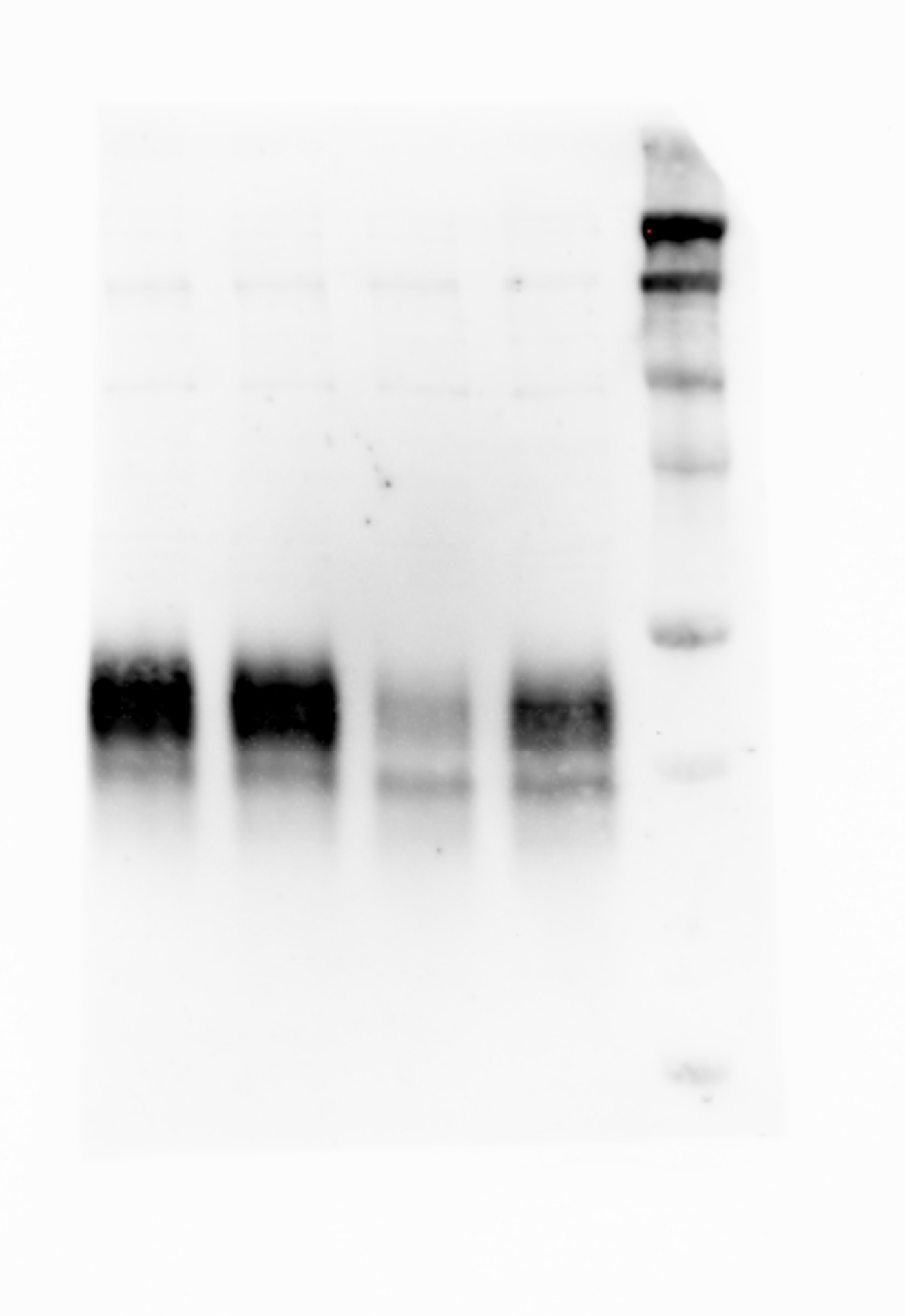

Supplement: Figure 6—figure supplement 1—source data 3. [file elife-93908-fig6-figsupp1-data3.zip › Figure 6S1C anti-ASGR1 with 4F3-RSPO2RA or IgG treatment Raw Data.tif]

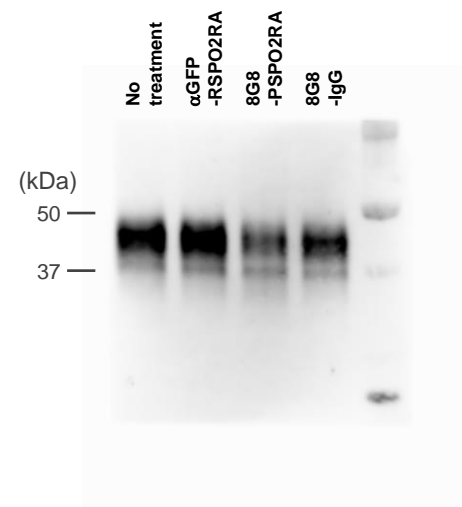

Supplement: Figure 6—figure supplement 1—source data 3. [file elife-93908-fig6-figsupp1-data3.zip › Figure 6S1C anti-ASGR1 with 8G8-RSPO2RA or IgG treatment Labelled Raw Data.pdf]

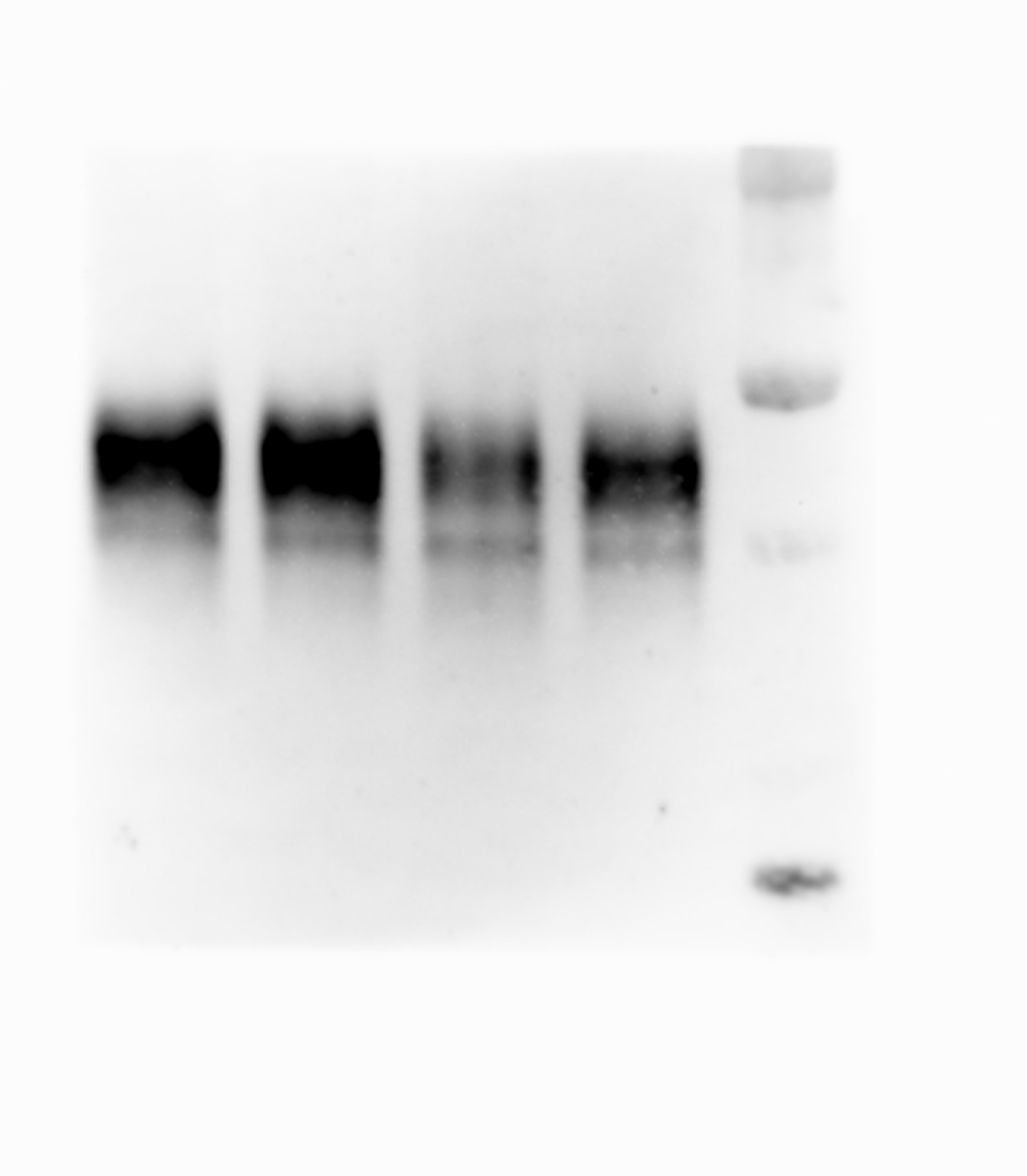

Supplement: Figure 6—figure supplement 1—source data 3. [file elife-93908-fig6-figsupp1-data3.zip › Figure 6S1C anti-ASGR1 with 8G8-RSPO2RA or IgG treatment Raw Data.tif]

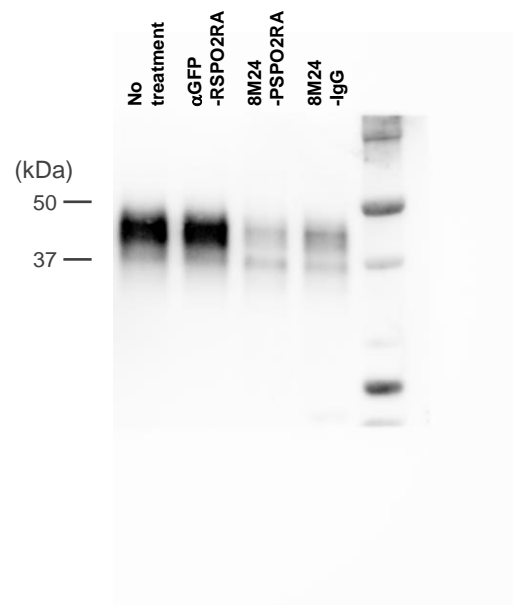

Supplement: Figure 6—figure supplement 1—source data 3. [file elife-93908-fig6-figsupp1-data3.zip › Figure 6S1C anti-ASGR1 with 8M24-RSPO2RA or IgG treatment Labelled Raw Data.pdf]

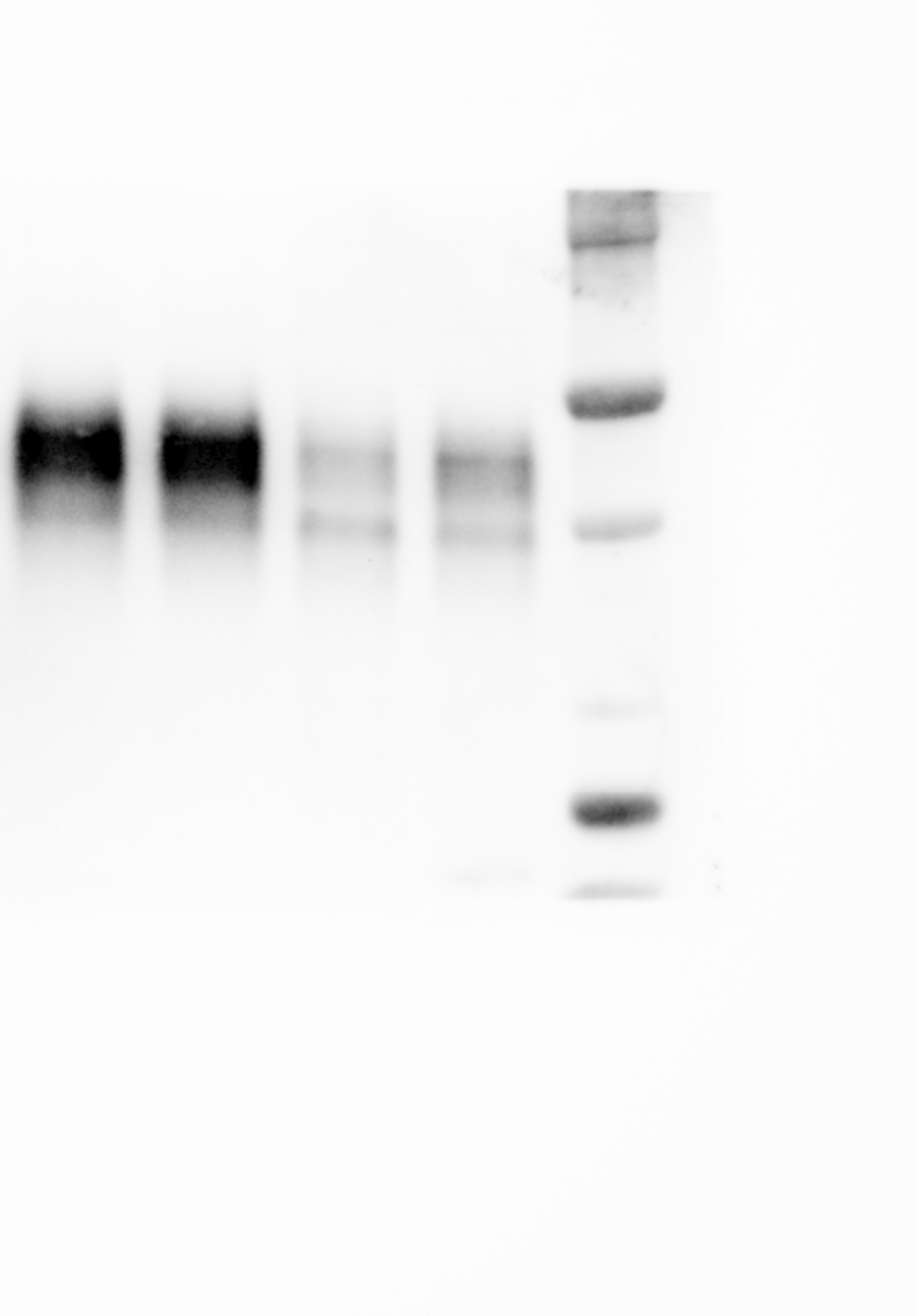

Supplement: Figure 6—figure supplement 1—source data 3. [file elife-93908-fig6-figsupp1-data3.zip › Figure 6S1C anti-ASGR1 with 8M24-RSPO2RA or IgG treatment Raw Data.tif]

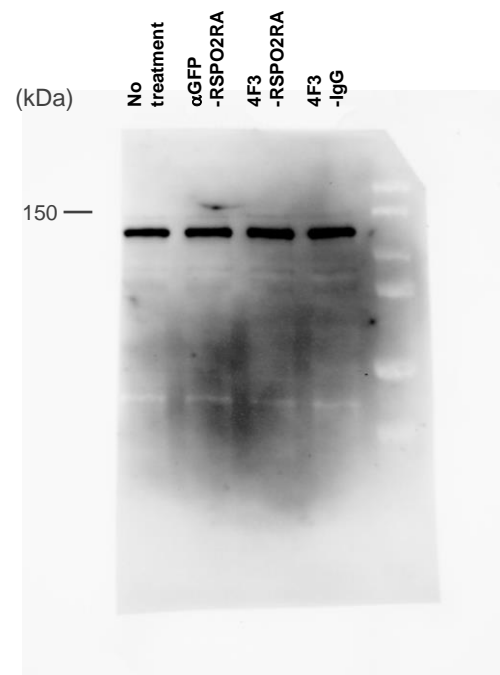

Supplement: Figure 6—figure supplement 1—source data 3. [file elife-93908-fig6-figsupp1-data3.zip › Figure 6S1C anti-Vinculin with 4F3-RSPO2RA or IgG treatment Labelled Raw Data.pdf]

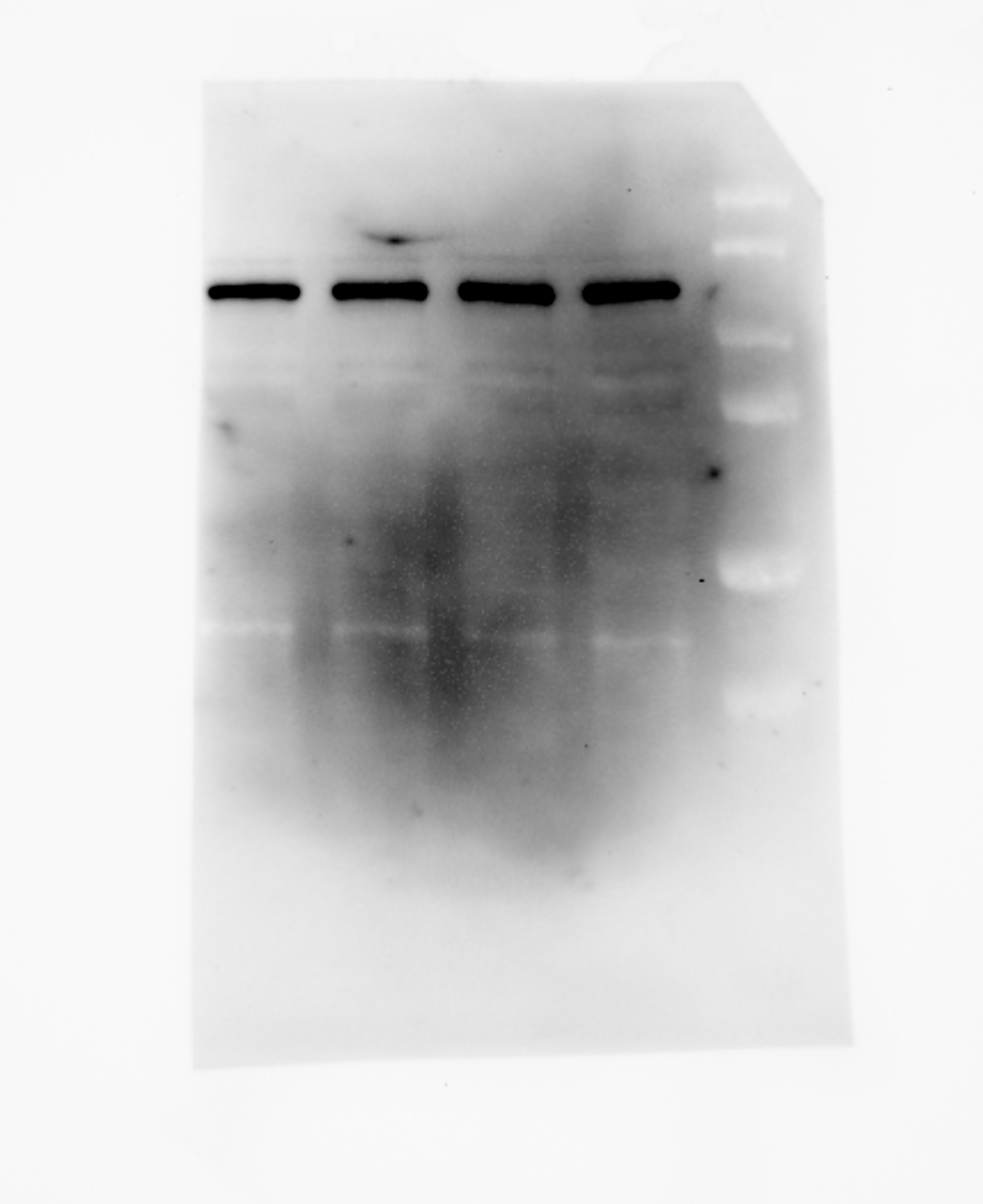

Supplement: Figure 6—figure supplement 1—source data 3. [file elife-93908-fig6-figsupp1-data3.zip › Figure 6S1C anti-Vinculin with 4F3-RSPO2RA or IgG treatment Raw Data.tif]

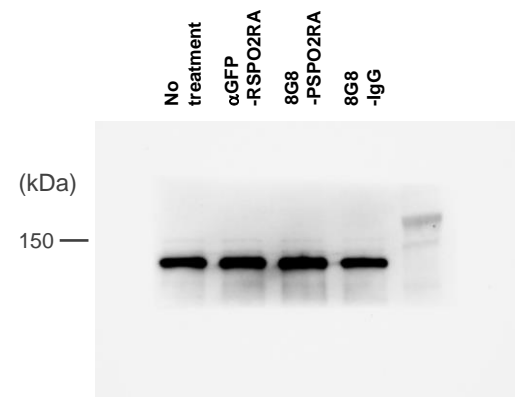

Supplement: Figure 6—figure supplement 1—source data 3. [file elife-93908-fig6-figsupp1-data3.zip › Figure 6S1C anti-Vinculin with 8G8-RSPO2RA or IgG treatment Labelled Raw Data.pdf]

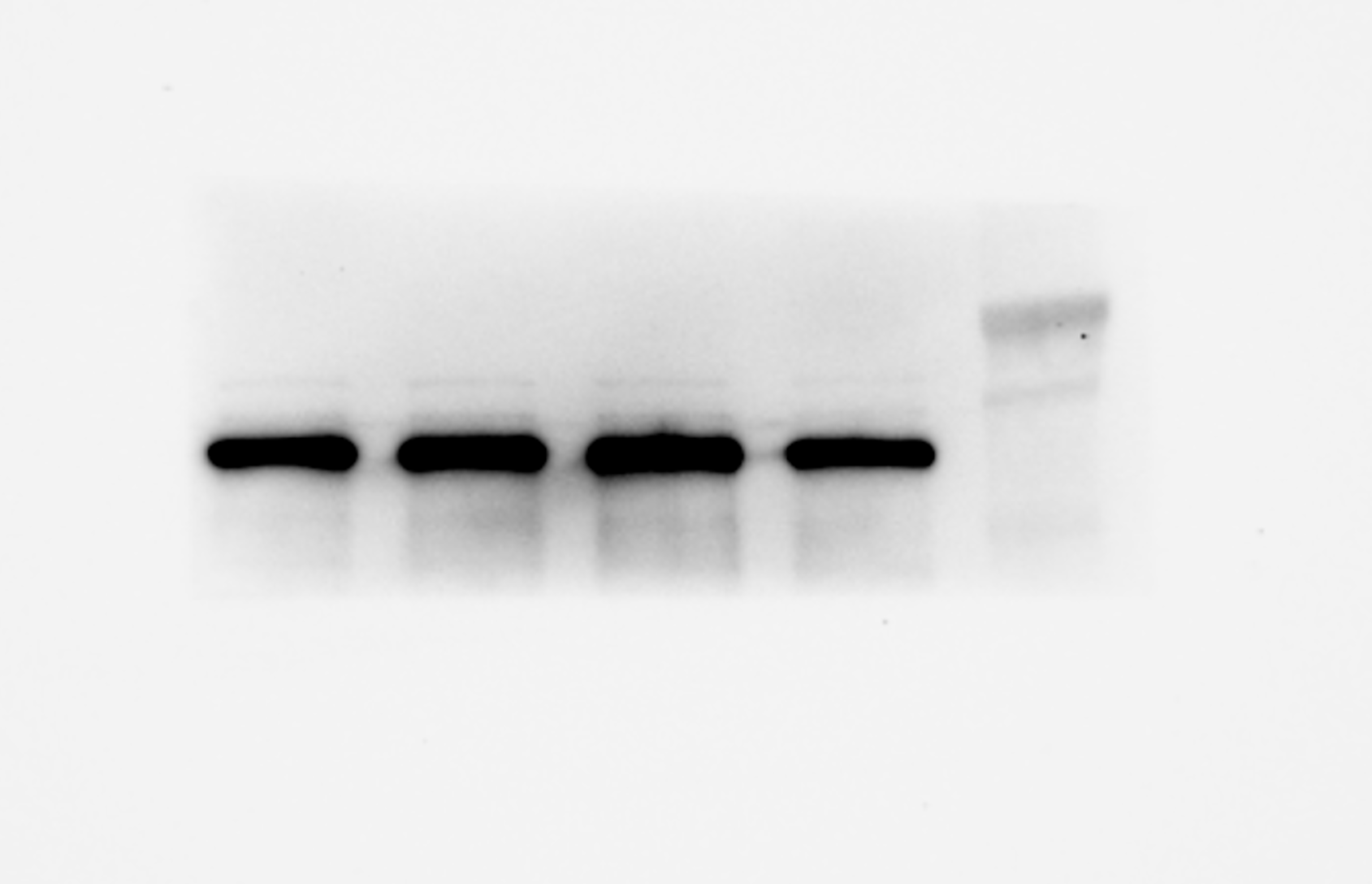

Supplement: Figure 6—figure supplement 1—source data 3. [file elife-93908-fig6-figsupp1-data3.zip › Figure 6S1C anti-Vinculin with 8G8-RSPO2RA or IgG treatment Raw Data.tif]

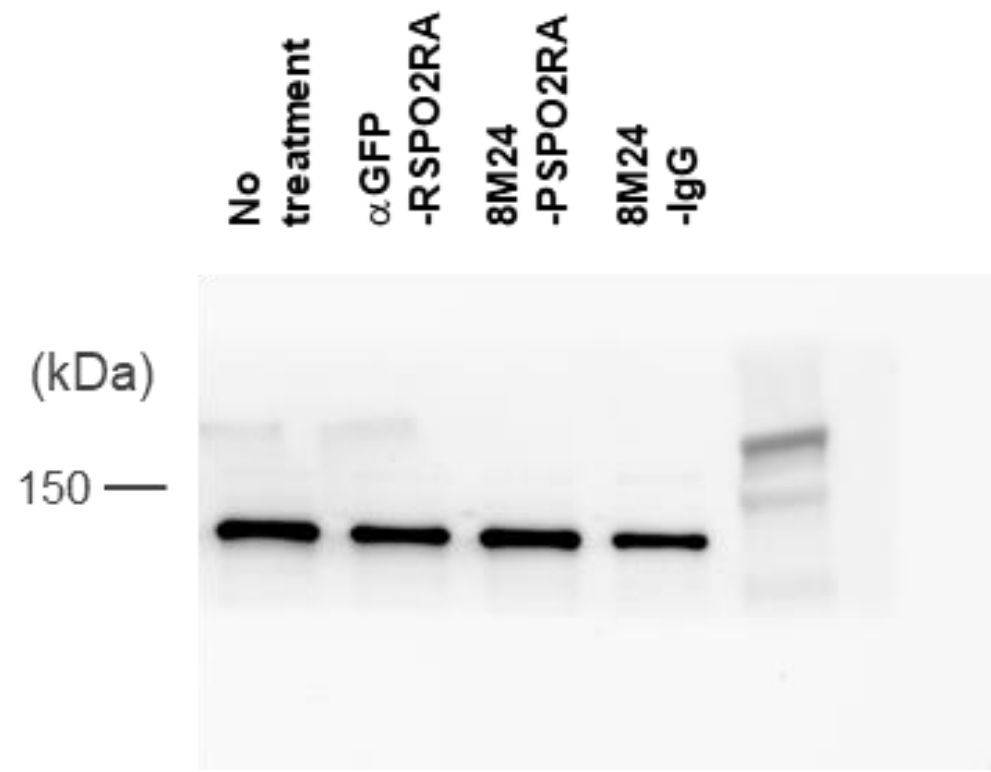

Supplement: Figure 6—figure supplement 1—source data 3. [file elife-93908-fig6-figsupp1-data3.zip › Figure 6S1C anti-Vinculin with 8M24-RSPO2RA or IgG treatment Labelled Raw Data.pdf]

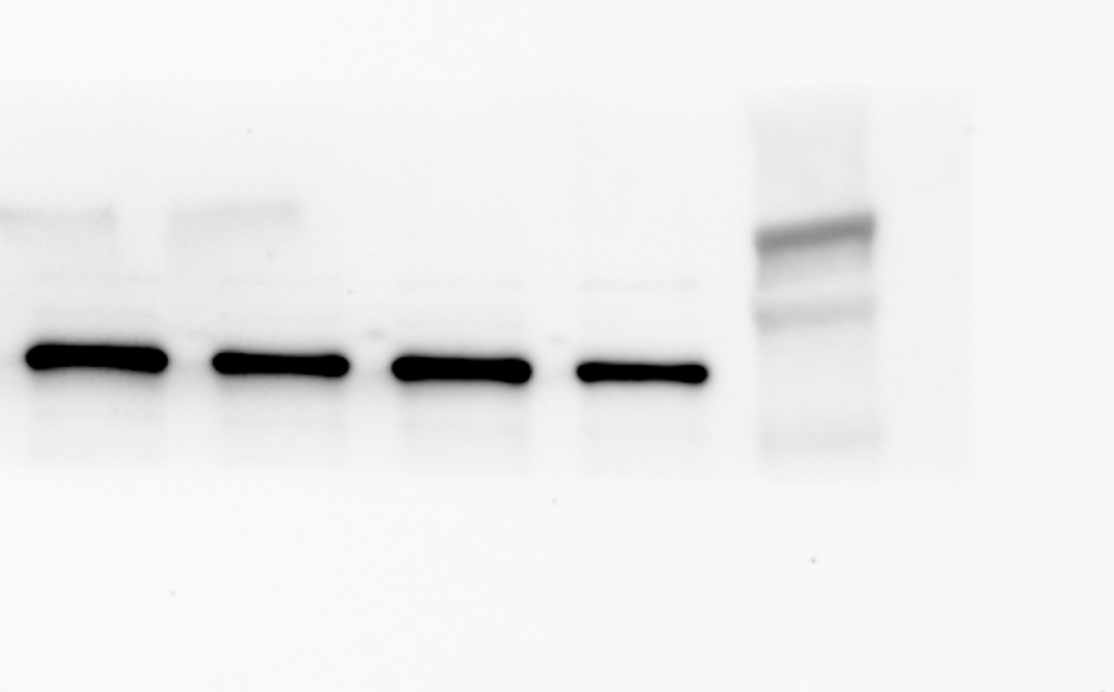

Supplement: Figure 6—figure supplement 1—source data 3. [file elife-93908-fig6-figsupp1-data3.zip › Figure 6S1C anti-Vinculin with 8M24-RSPO2RA or IgG treatment Raw Data.tif]

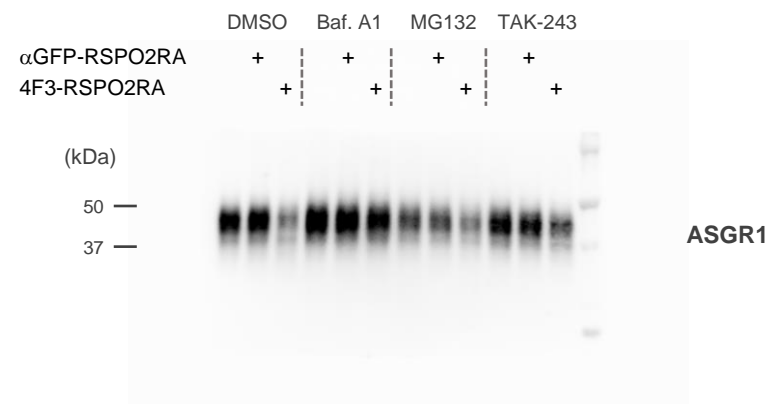

Supplement: Figure 6—figure supplement 1—source data 4. [file elife-93908-fig6-figsupp1-data4.zip › Figure 6S1D anti-ASGR1 with 4F3-RSPO2RA treatment Labelled Raw Data.pdf]

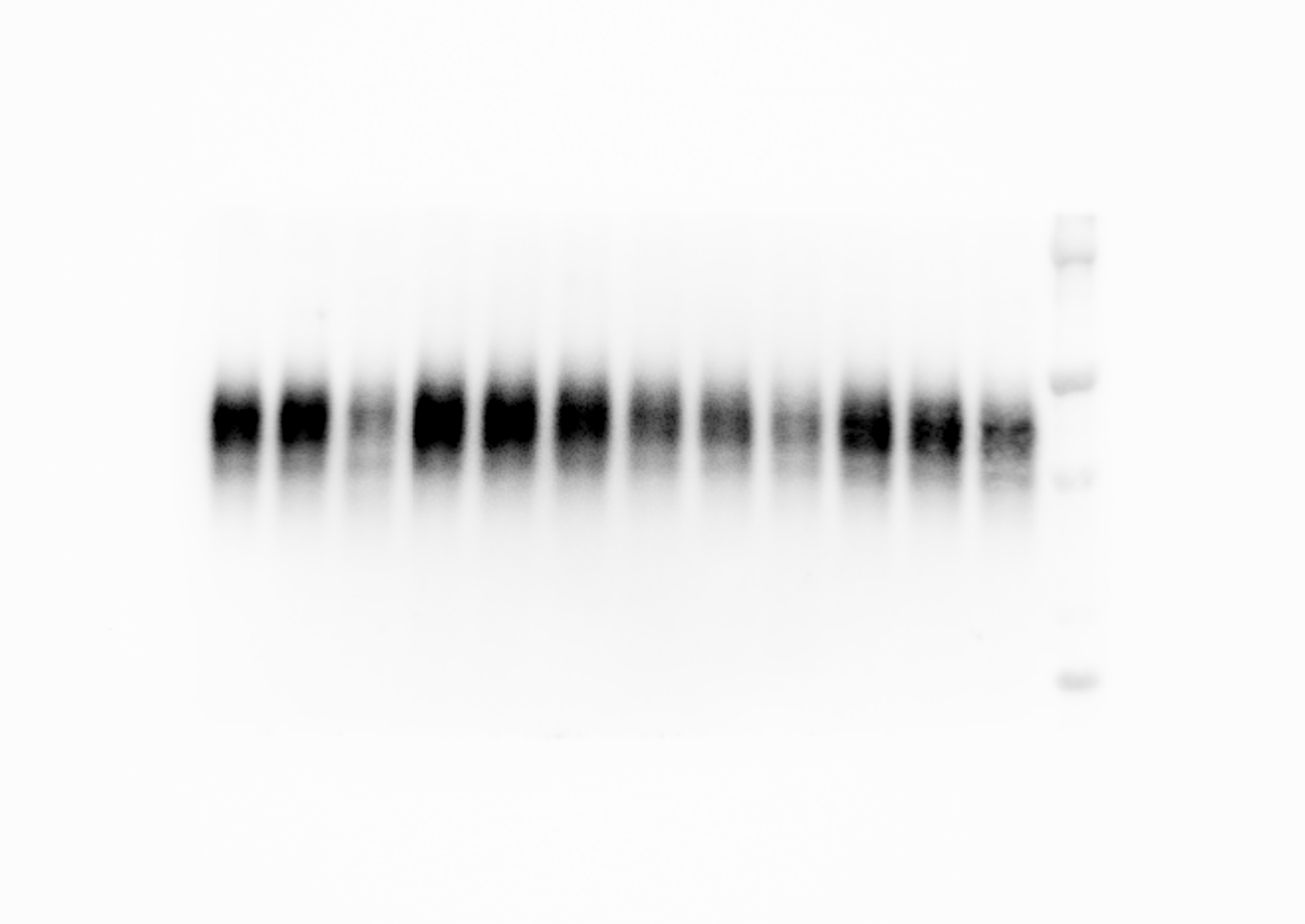

Supplement: Figure 6—figure supplement 1—source data 4. [file elife-93908-fig6-figsupp1-data4.zip › Figure 6S1D anti-ASGR1 with 4F3-RSPO2RA treatment Raw Data.tif]

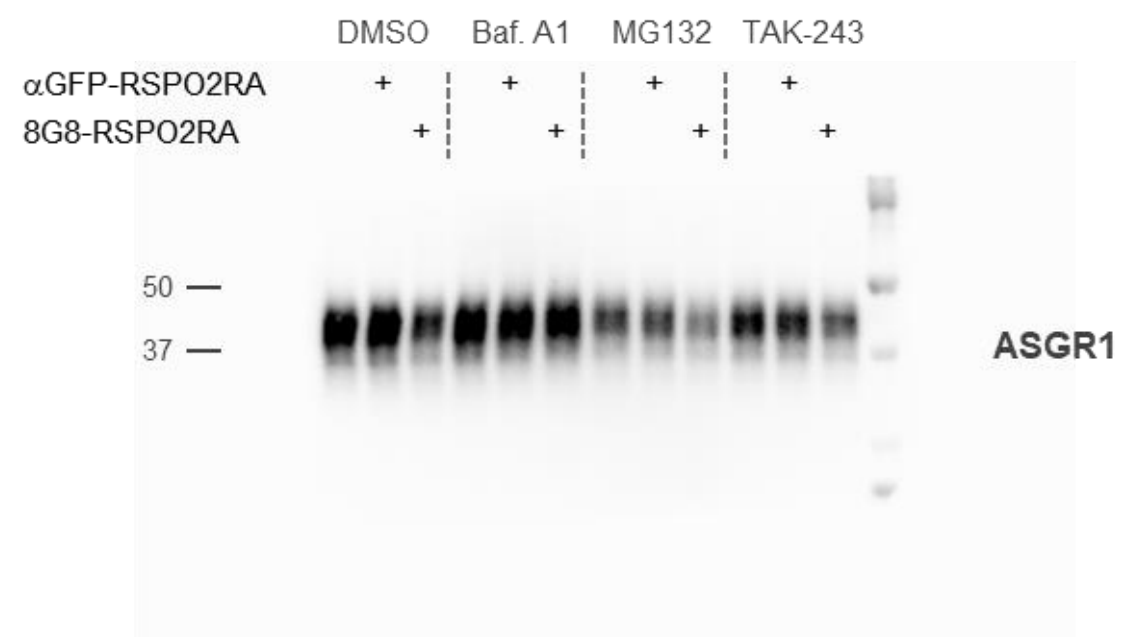

Supplement: Figure 6—figure supplement 1—source data 4. [file elife-93908-fig6-figsupp1-data4.zip › Figure 6S1D anti-ASGR1 with 8G8-RSPO2RA treatment Labelled Raw Data.pdf]

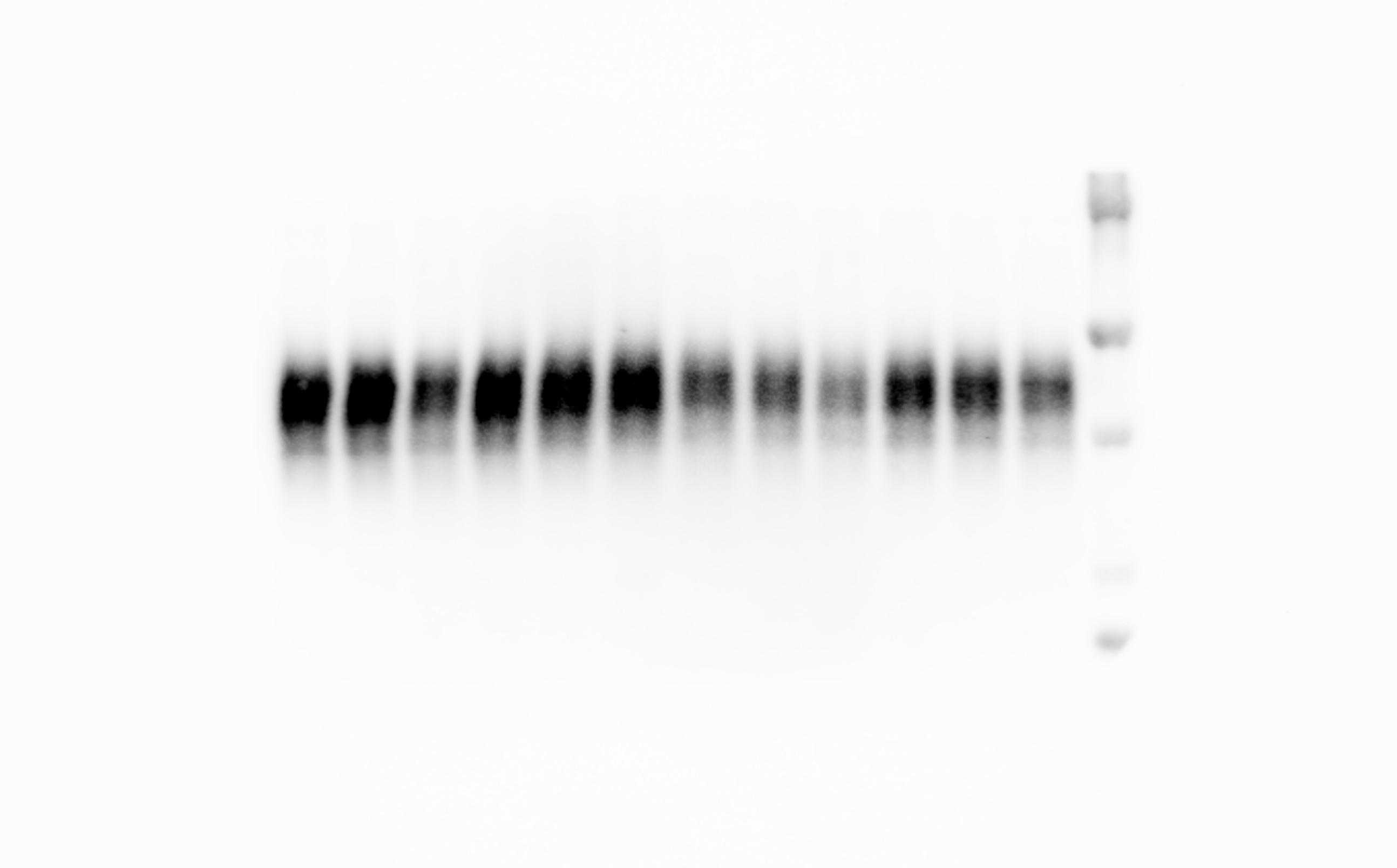

Supplement: Figure 6—figure supplement 1—source data 4. [file elife-93908-fig6-figsupp1-data4.zip › Figure 6S1D anti-ASGR1 with 8G8-RSPO2RA treatment Raw Data.tif]
