# Supplementary figures and images for: Targeted protein degradation systems to enhance Wnt signaling (part 2 of 2)
Source: eLife. 2024 Jun 7;13:RP93908. doi: 10.7554/eLife.93908 (PMC11161174; doi:10.7554/eLife.93908)

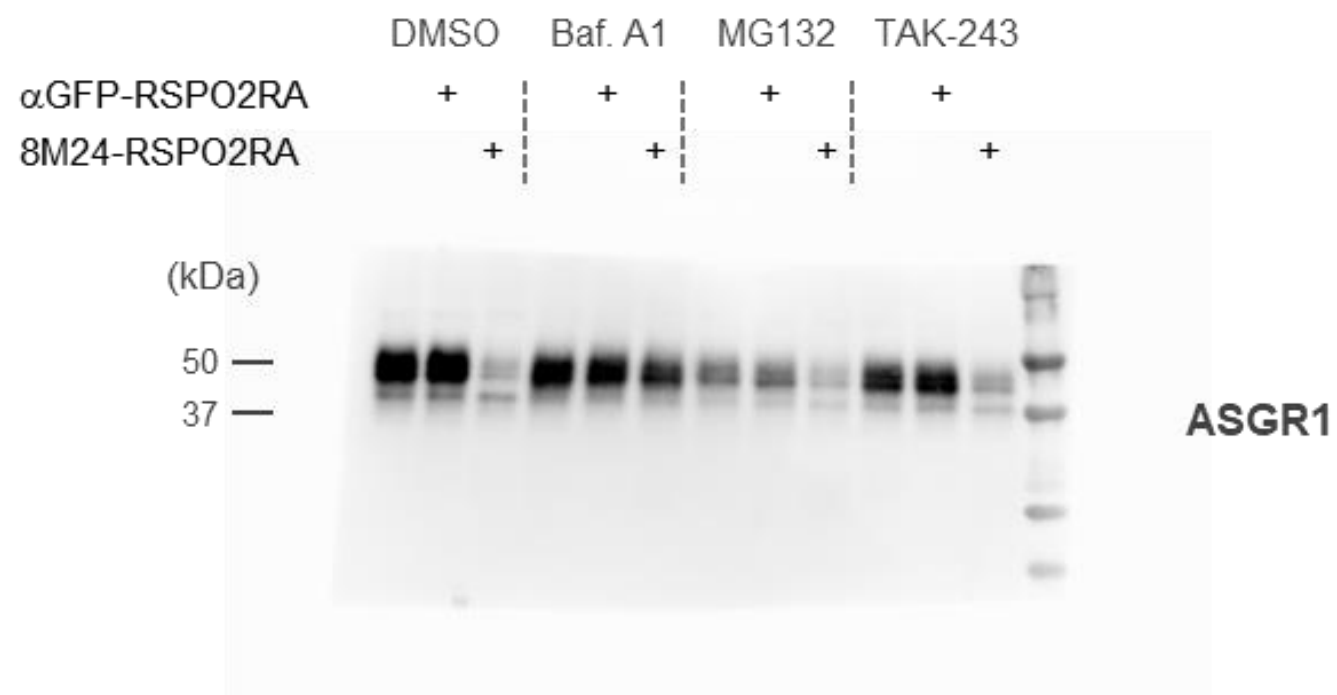

Supplement: Figure 6—figure supplement 1—source data 4. [file elife-93908-fig6-figsupp1-data4.zip › Figure 6S1D anti-ASGR1 with 8M24-RSPO2RA treatment Labelled Raw Data.pdf]

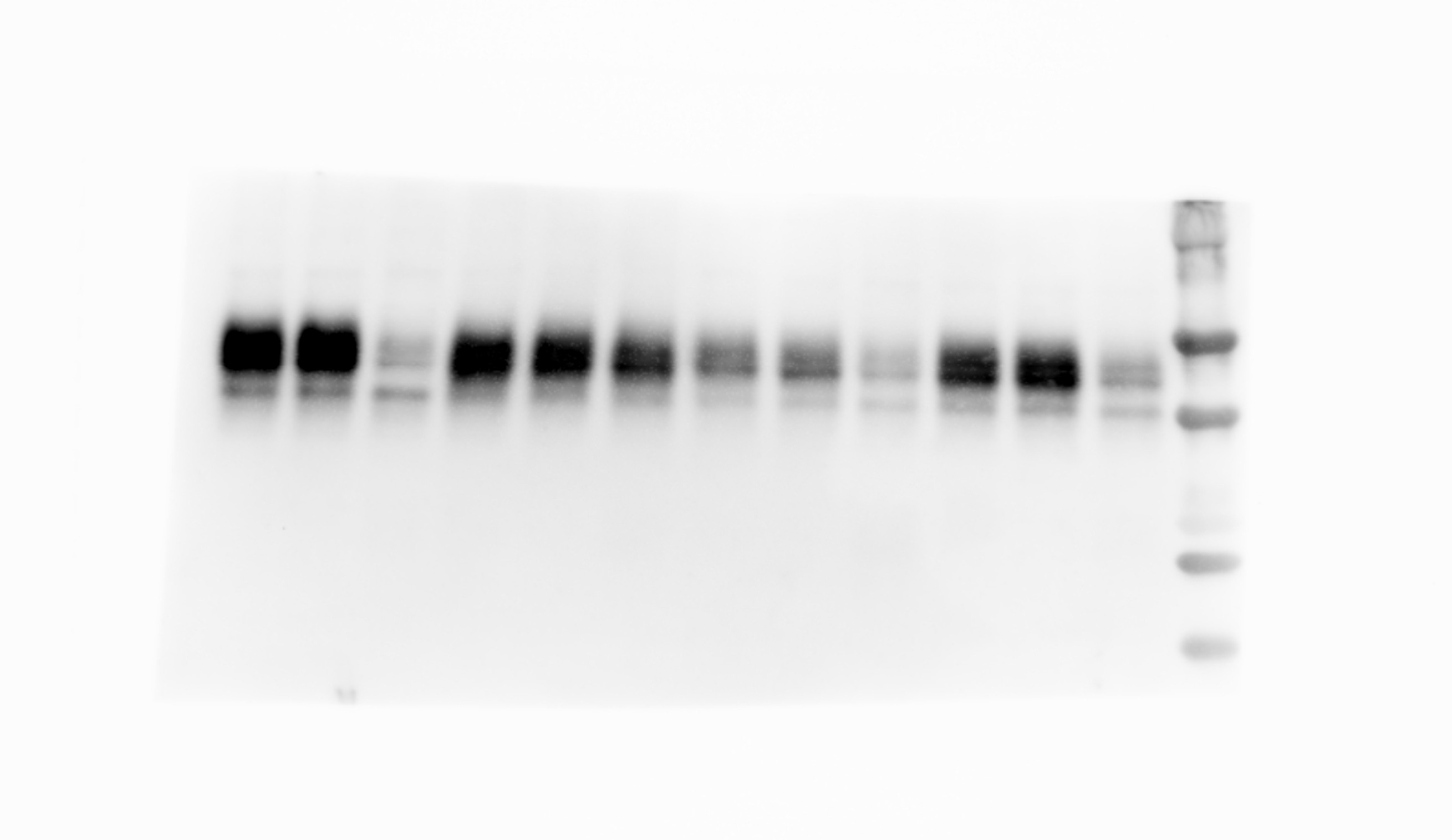

Supplement: Figure 6—figure supplement 1—source data 4. [file elife-93908-fig6-figsupp1-data4.zip › Figure 6S1D anti-ASGR1 with 8M24-RSPO2RA treatment Raw Data.tif]

|                      | DMSO | Baf. A1 | MG132 | TAK-243 |
|----------------------|------|---------|-------|---------|
| $\alpha$ GFP-RSPO2RA | +    | +       | +     | +       |
| 8M24-RSPO2RA         | +    | +       | +     | +       |

(kDa)

20 —  
15 —  
10 —

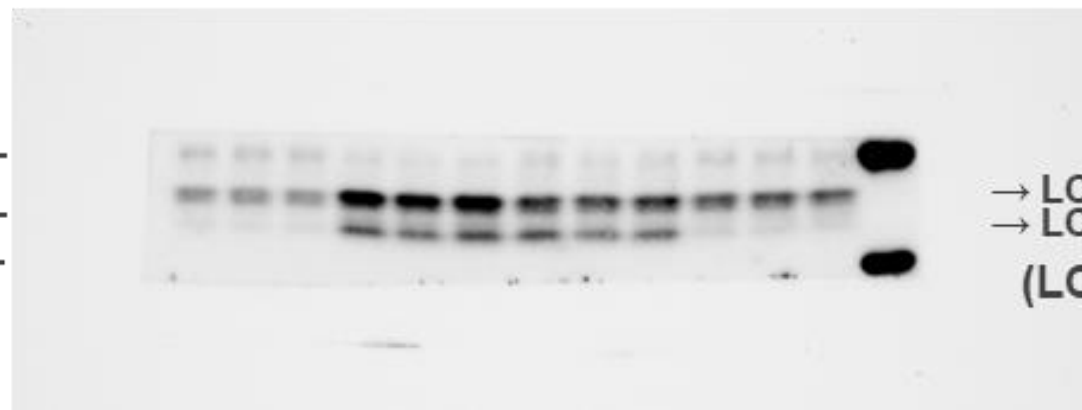

→ LC3B-I  
→ LC3B-II  
(LC3B)

Supplement: Figure 6—figure supplement 1—source data 4. [file elife-93908-fig6-figsupp1-data4.zip › Figure 6S1D anti-LC3B 8M24-RSPO2RA treatment Labelled Raw Data.pdf]

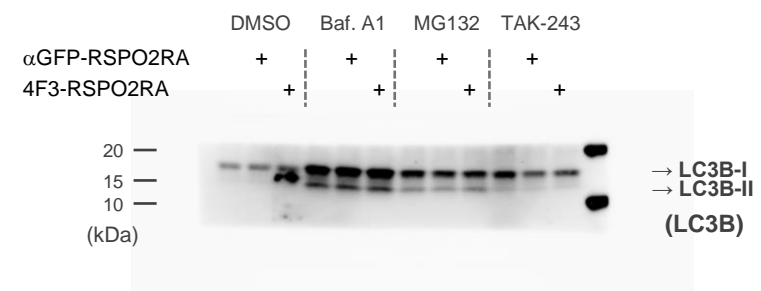

Supplement: Figure 6—figure supplement 1—source data 4. [file elife-93908-fig6-figsupp1-data4.zip › Figure 6S1D anti-LC3B with 4F3-RSPO2RA treatment Labelled Raw Data.pdf]

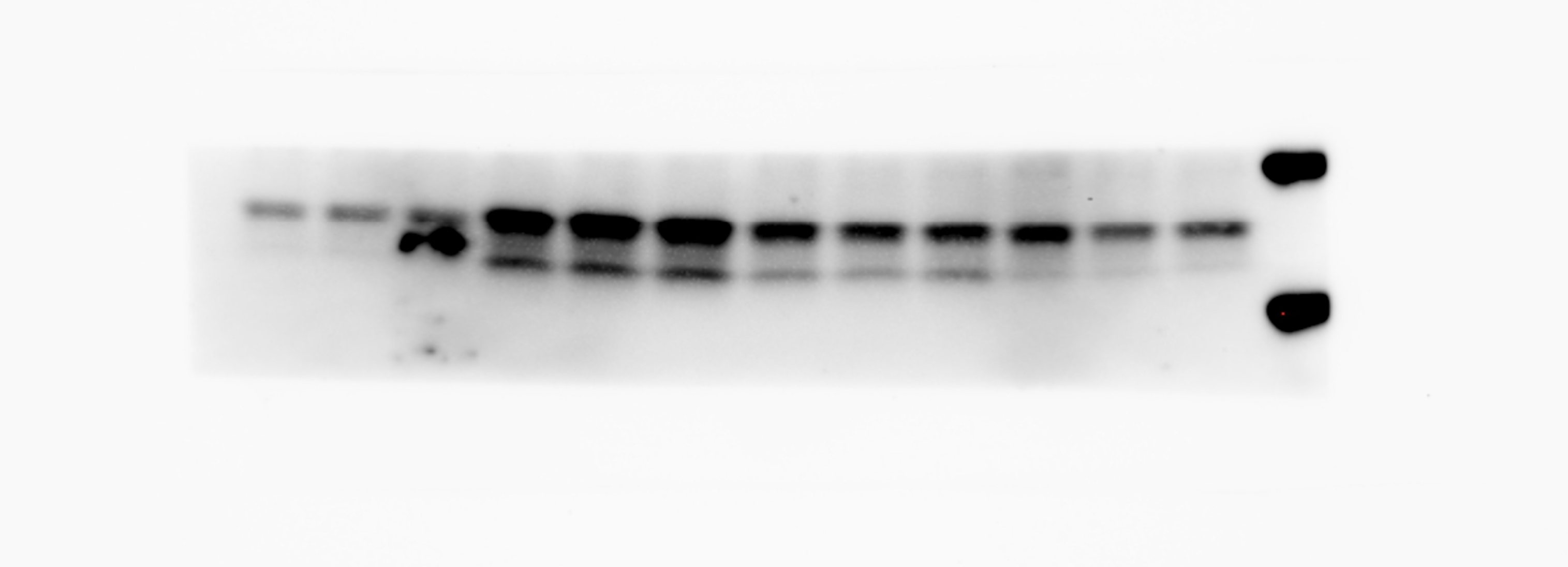

Supplement: Figure 6—figure supplement 1—source data 4. [file elife-93908-fig6-figsupp1-data4.zip › Figure 6S1D anti-LC3B with 4F3-RSPO2RA treatment Raw Data.tif]

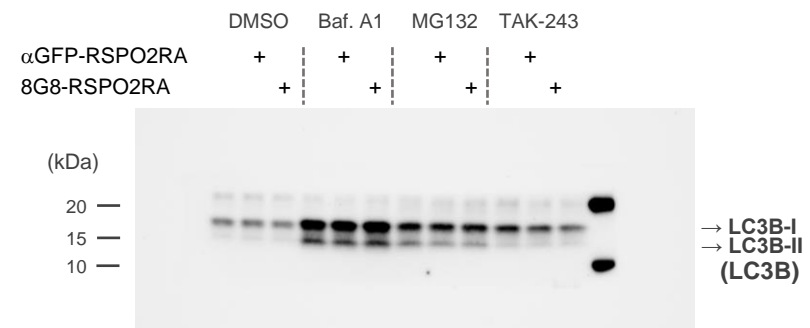

Supplement: Figure 6—figure supplement 1—source data 4. [file elife-93908-fig6-figsupp1-data4.zip › Figure 6S1D anti-LC3B with 8G8-RSPO2RA treatment Labelled Raw Data.pdf]

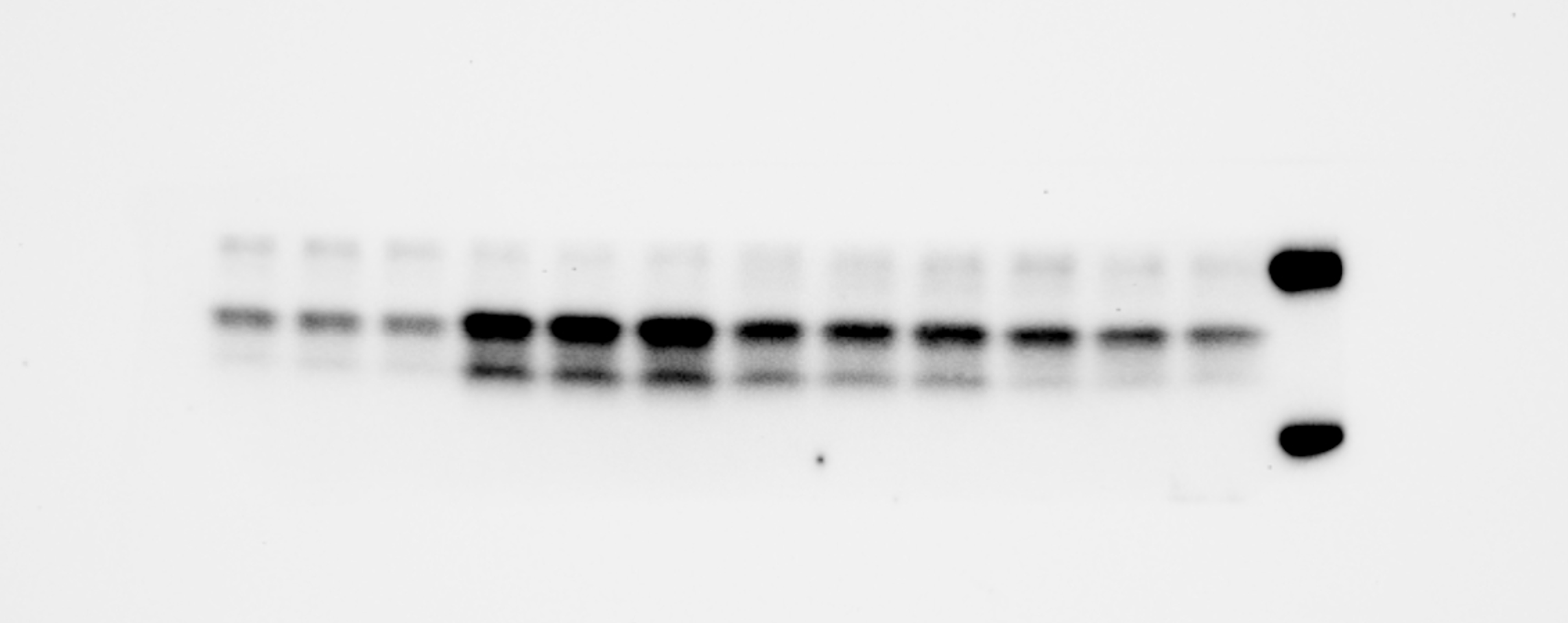

Supplement: Figure 6—figure supplement 1—source data 4. [file elife-93908-fig6-figsupp1-data4.zip › Figure 6S1D anti-LC3B with 8G8-RSPO2RA treatment Raw Data.tif]

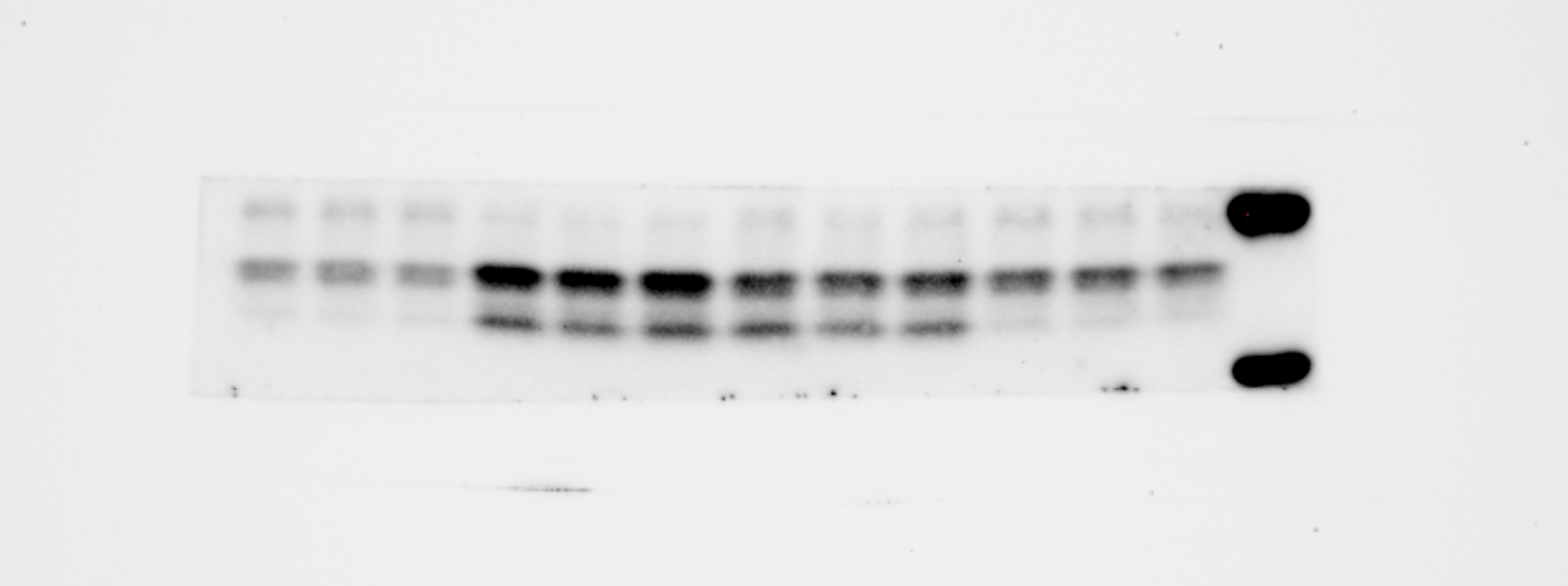

Supplement: Figure 6—figure supplement 1—source data 4. [file elife-93908-fig6-figsupp1-data4.zip › Figure 6S1D anti-LC3B with 8M24-RSPO2RA treatment Raw Data.tif]

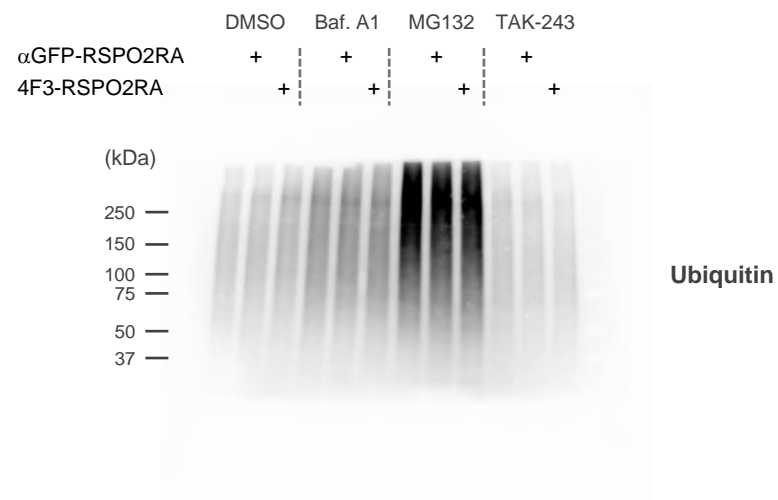

Supplement: Figure 6—figure supplement 1—source data 4. [file elife-93908-fig6-figsupp1-data4.zip › Figure 6S1D anti-Ubiquitin with 4F3-RSPO2RA treatment Labelled Raw Data.pdf]

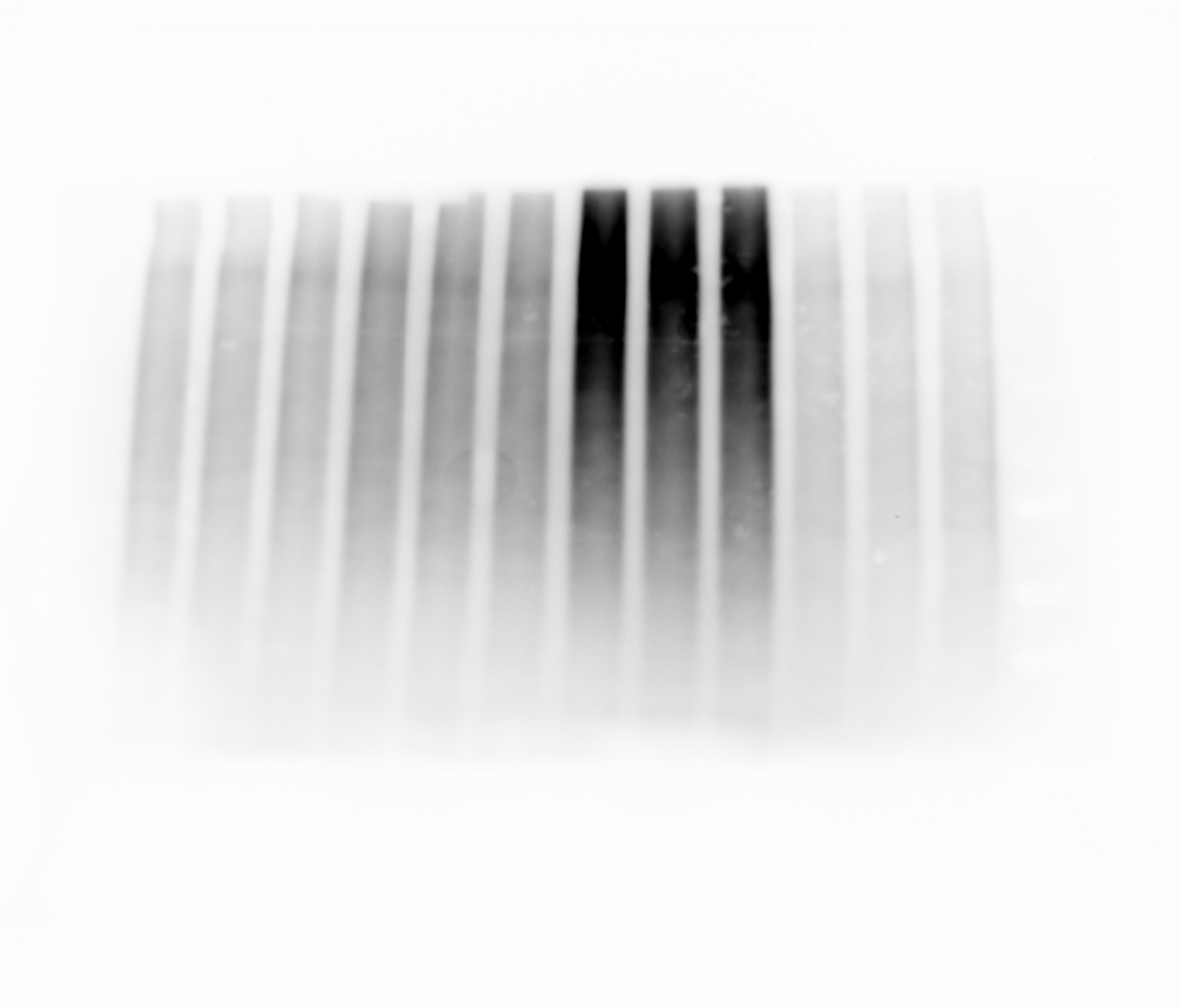

Supplement: Figure 6—figure supplement 1—source data 4. [file elife-93908-fig6-figsupp1-data4.zip › Figure 6S1D anti-Ubiquitin with 4F3-RSPO2RA treatment Raw Data.tif]

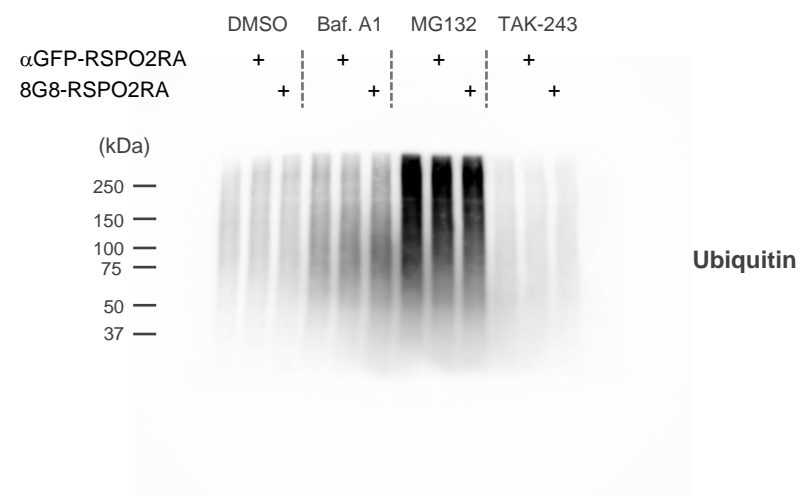

Supplement: Figure 6—figure supplement 1—source data 4. [file elife-93908-fig6-figsupp1-data4.zip › Figure 6S1D anti-Ubiquitin with 8G8-RSPO2RA treatment Labelled Raw Data.pdf]

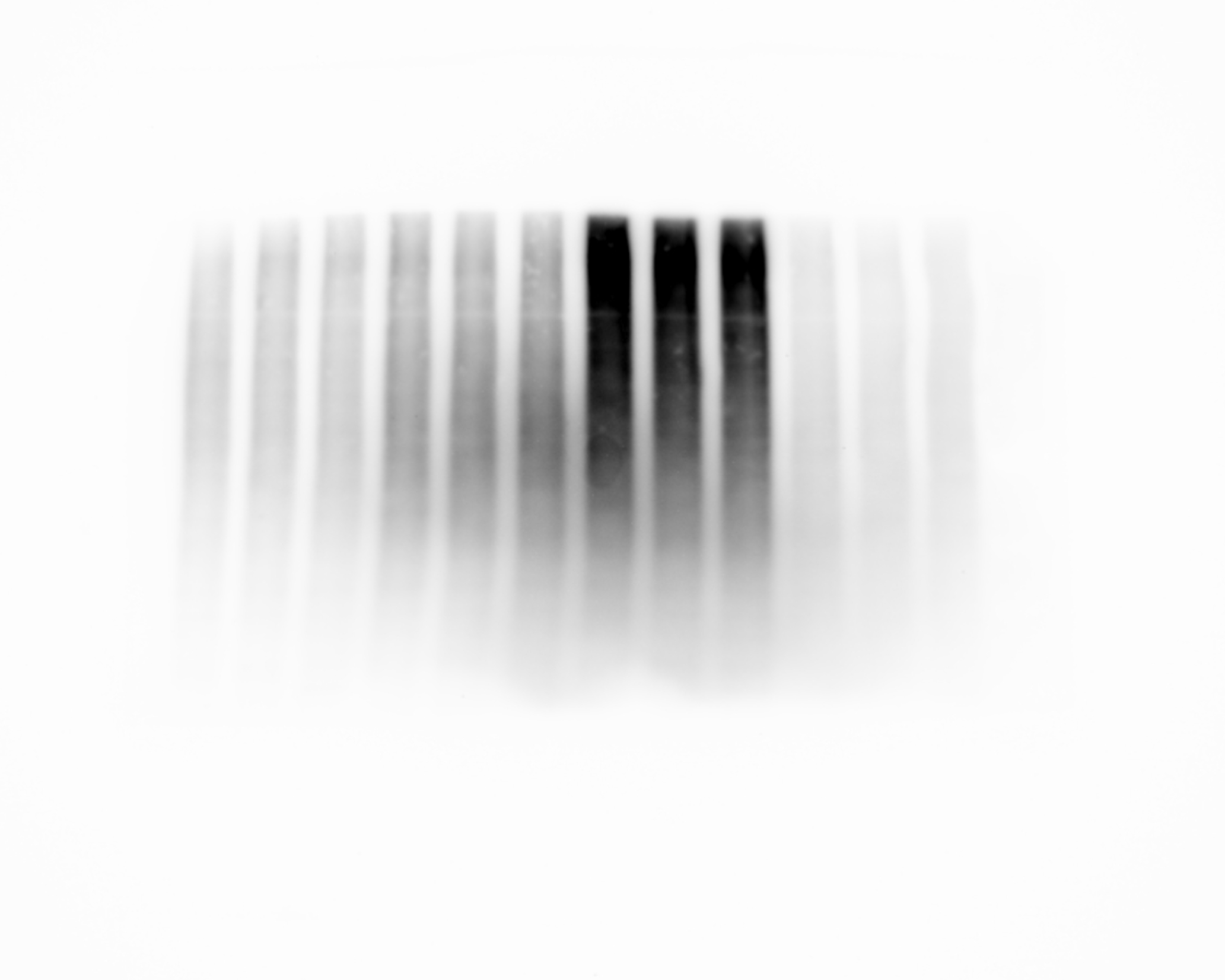

Supplement: Figure 6—figure supplement 1—source data 4. [file elife-93908-fig6-figsupp1-data4.zip › Figure 6S1D anti-Ubiquitin with 8G8-RSPO2RA treatment Raw Data.tif]

|                      | DMSO | Baf. A1 | MG132 | TAK-243 |
|----------------------|------|---------|-------|---------|
| $\alpha$ GFP-RSPO2RA | +    | +       | +     | +       |
| 8M24-RSPO2RA         | +    | +       | +     | +       |

(kDa)

250 —  
150 —  
100 —  
75 —  
50 —  
37 —

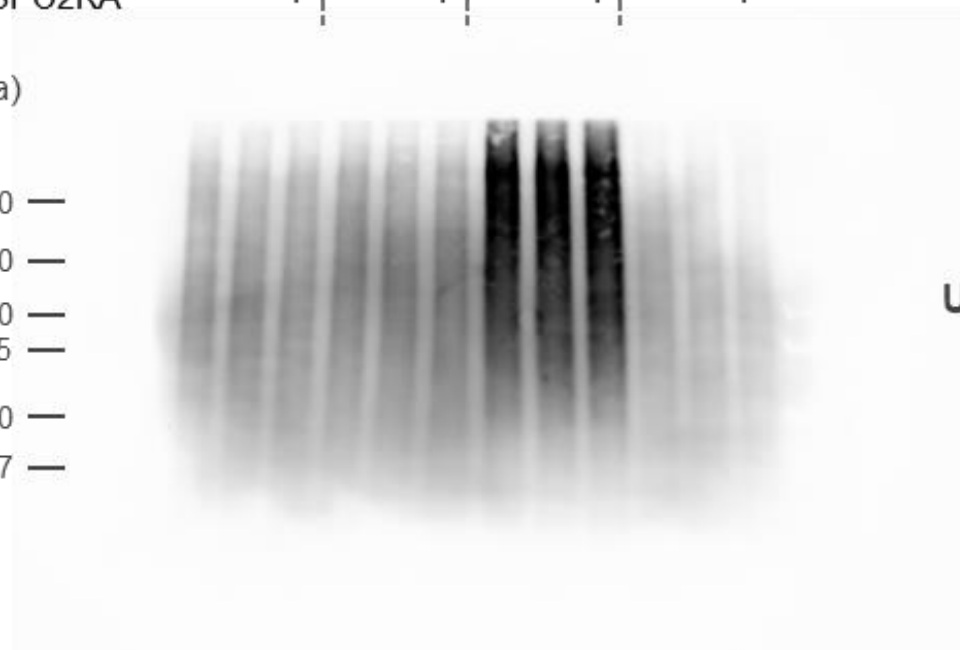

Ubiquitin

Supplement: Figure 6—figure supplement 1—source data 4. [file elife-93908-fig6-figsupp1-data4.zip › Figure 6S1D anti-Ubiquitin with 8M24-RSPO2RA treatment Labelled Raw Data.pdf]

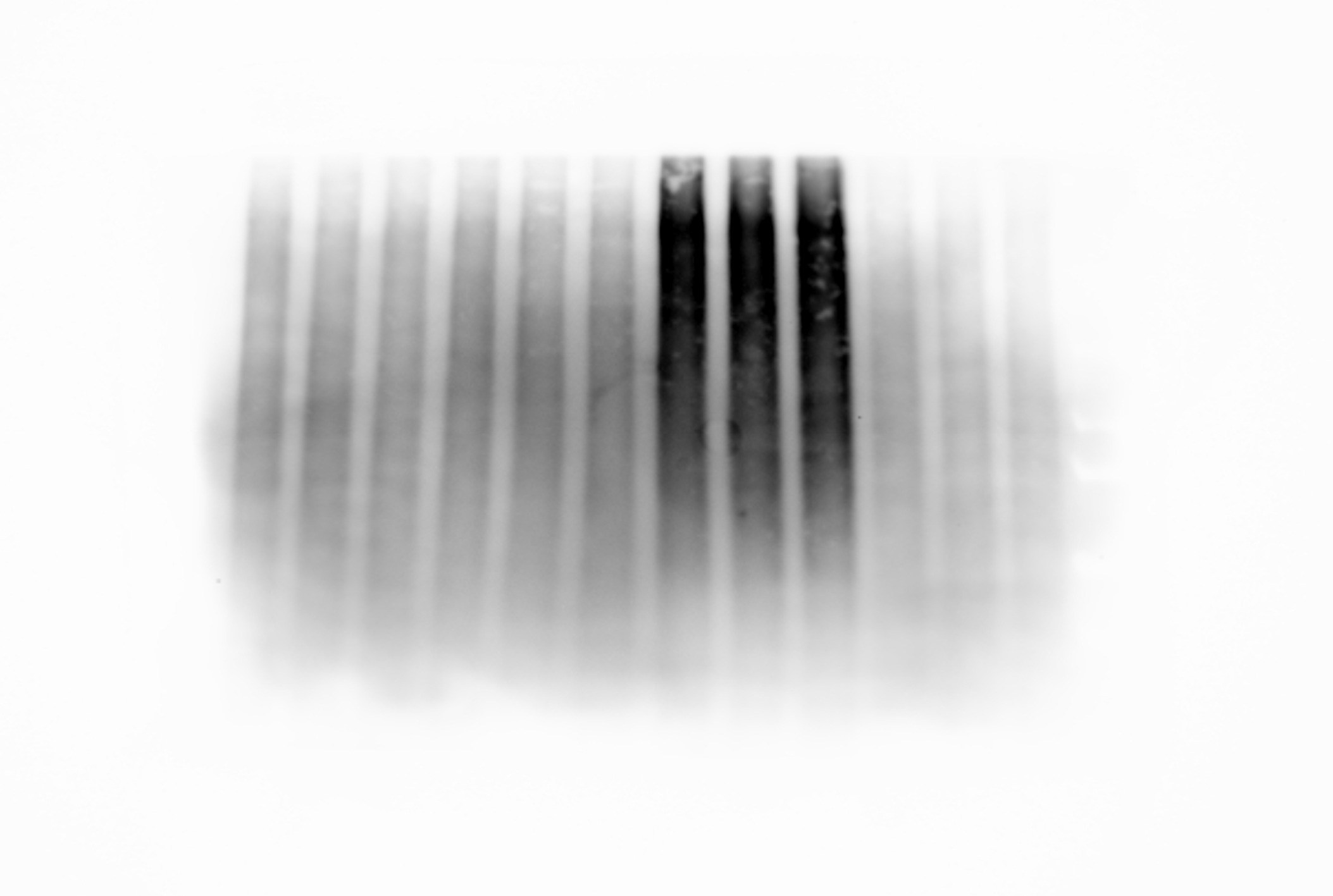

Supplement: Figure 6—figure supplement 1—source data 4. [file elife-93908-fig6-figsupp1-data4.zip › Figure 6S1D anti-Ubiquitin with 8M24-RSPO2RA treatment Raw Data.tif]

|                      | DMSO | Baf. A1 | MG132 | TAK-243 |
|----------------------|------|---------|-------|---------|
| $\alpha$ GFP-RSPO2RA | +    | +       | +     | +       |
| 4F3-RSPO2RA          | +    | +       | +     | +       |

(kDa)  
150 —

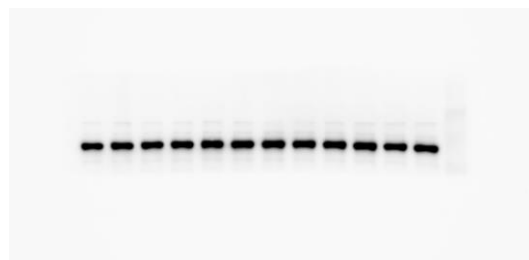

**Vinculin**

Supplement: Figure 6—figure supplement 1—source data 4. [file elife-93908-fig6-figsupp1-data4.zip › Figure 6S1D anti-Vinculin with 4F3-RSPO2RA treatment Labelled Raw Data.pdf]

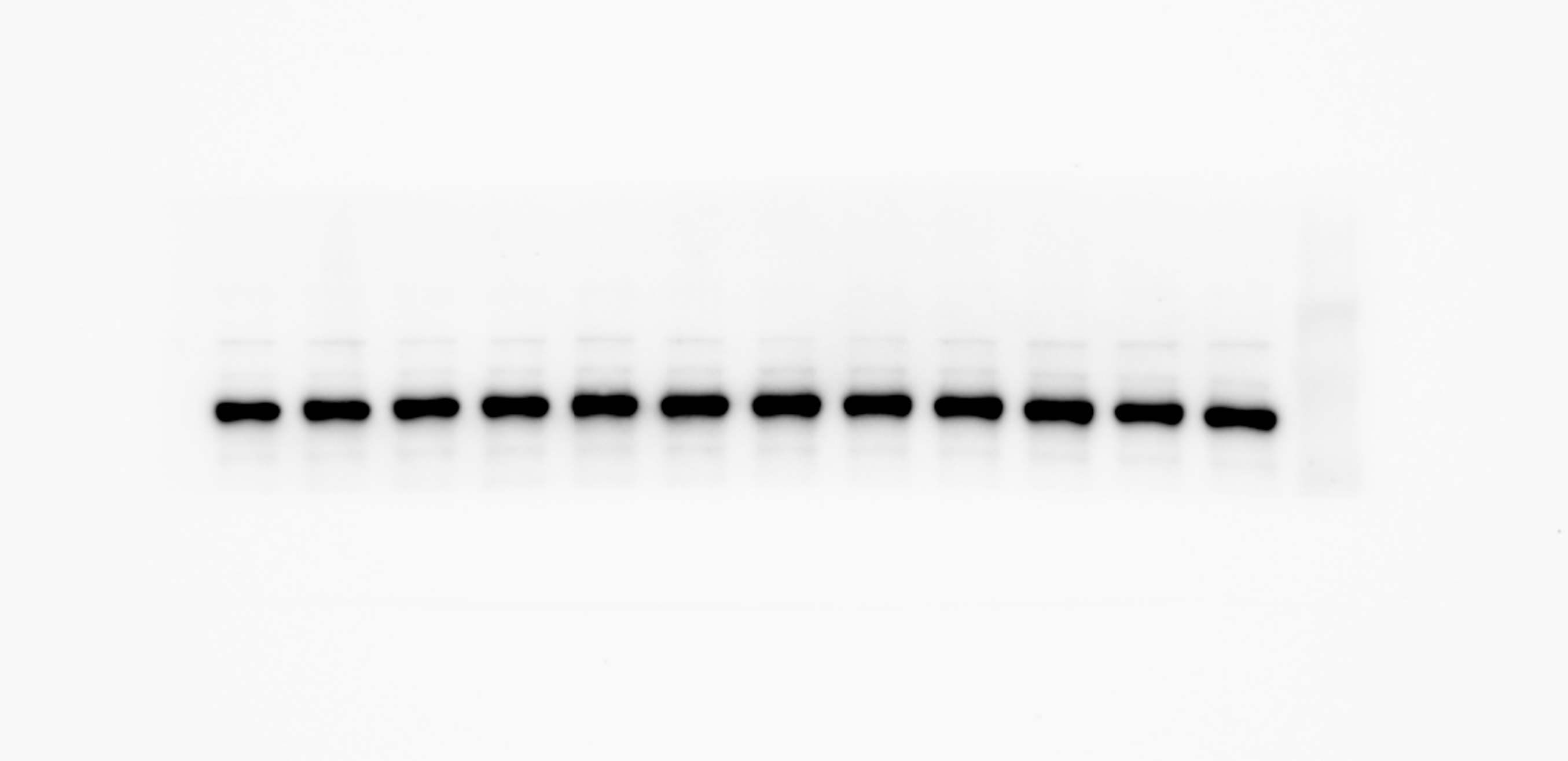

Supplement: Figure 6—figure supplement 1—source data 4. [file elife-93908-fig6-figsupp1-data4.zip › Figure 6S1D anti-Vinculin with 4F3-RSPO2RA treatment Raw Data.tif]

|                      | DMSO | Baf. A1 | MG132 | TAK-243 |
|----------------------|------|---------|-------|---------|
| $\alpha$ GFP-RSP02RA | +    | +       | +     | +       |
| 8G8-RSP02RA          | +    | +       | +     | +       |

(kDa)

150 —

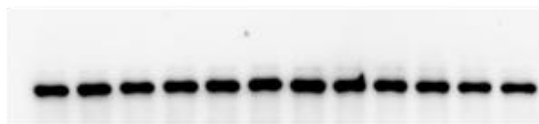

Vinculin

Supplement: Figure 6—figure supplement 1—source data 4. [file elife-93908-fig6-figsupp1-data4.zip › Figure 6S1D anti-Vinculin with 8G8-RSPO2RA treatment Labelled Raw Data.pdf]

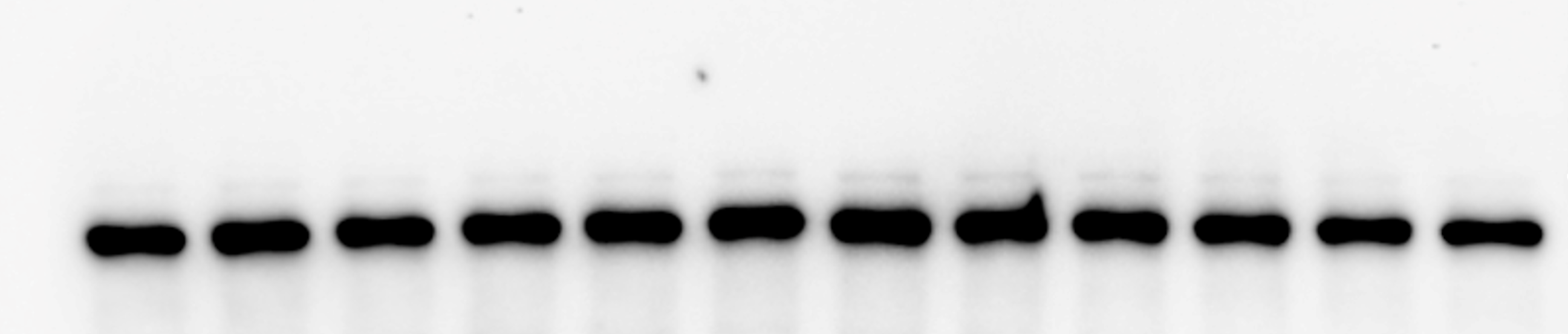

Supplement: Figure 6—figure supplement 1—source data 4. [file elife-93908-fig6-figsupp1-data4.zip › Figure 6S1D anti-Vinculin with 8G8-RSPO2RA treatment Raw Data.tif]

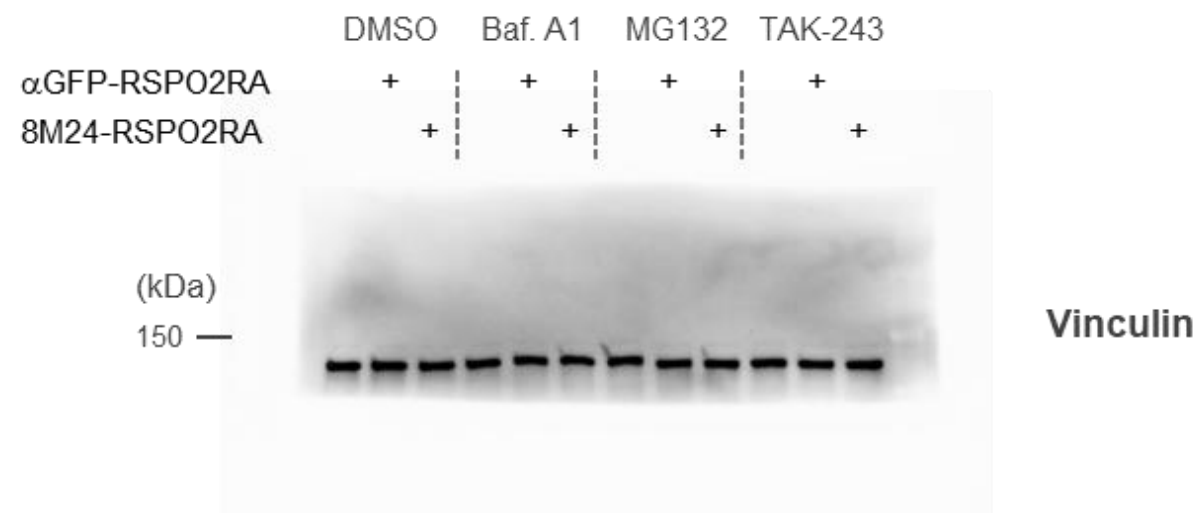

Supplement: Figure 6—figure supplement 1—source data 4. [file elife-93908-fig6-figsupp1-data4.zip › Figure 6S1D anti-Vinculin with 8M24-RSPO2RA treatment Labelled Raw Data.pdf]

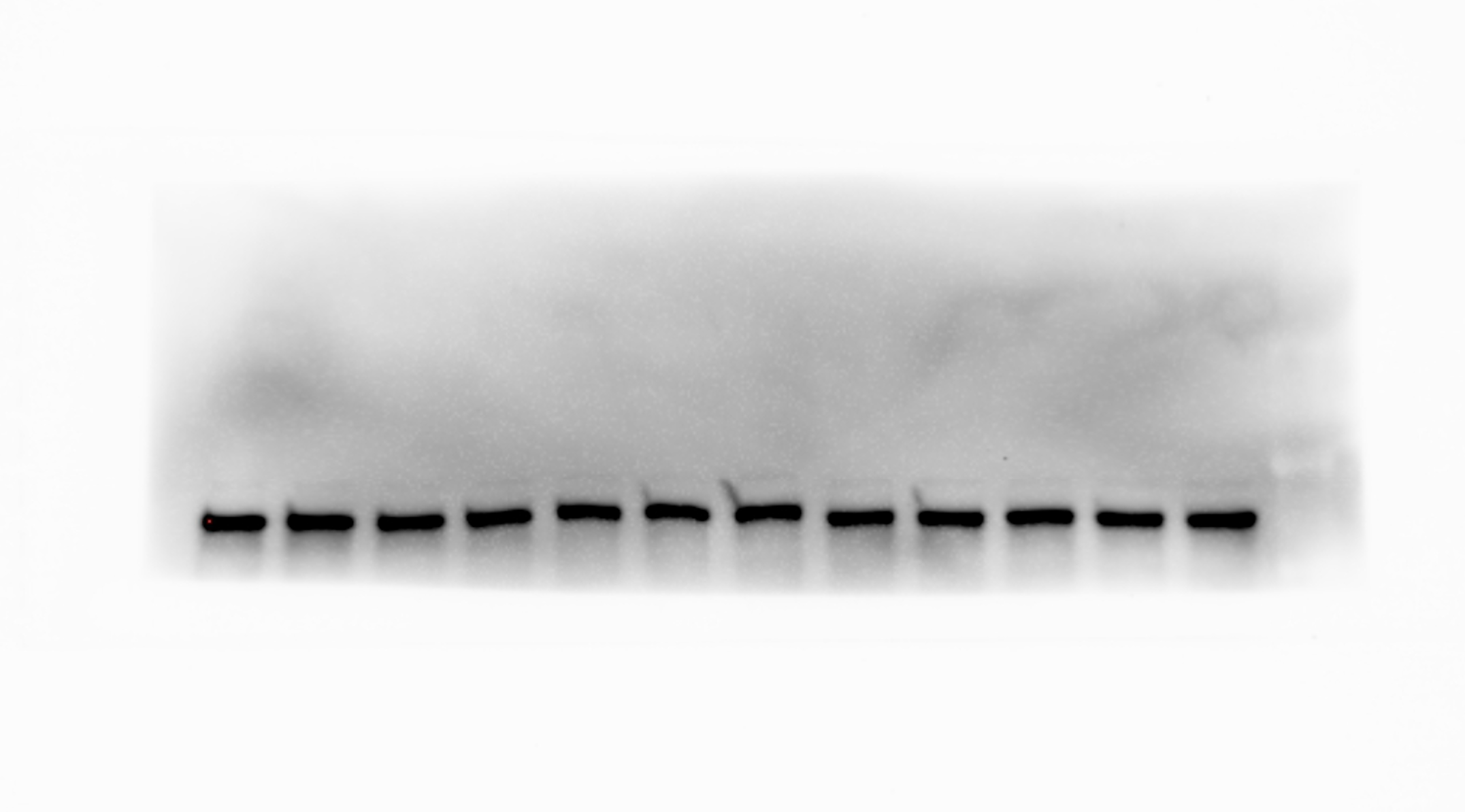

Supplement: Figure 6—figure supplement 1—source data 4. [file elife-93908-fig6-figsupp1-data4.zip › Figure 6S1D anti-Vinculin with 8M24-RSPO2RA treatment Raw Data.tif]

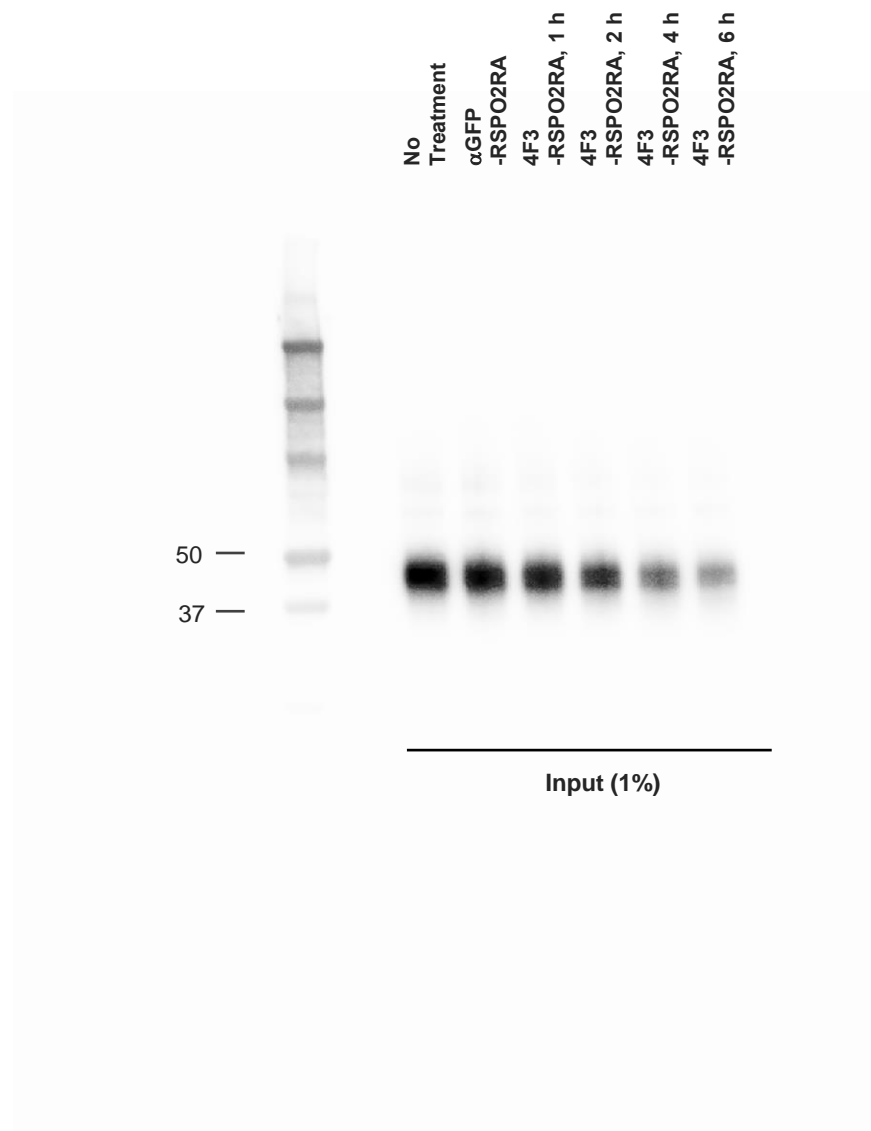

Supplement: Figure 7—source data 1. [file elife-93908-fig7-data1.zip › Figure 7A input, anti-ASGR1 Labelled Raw Data.pdf]

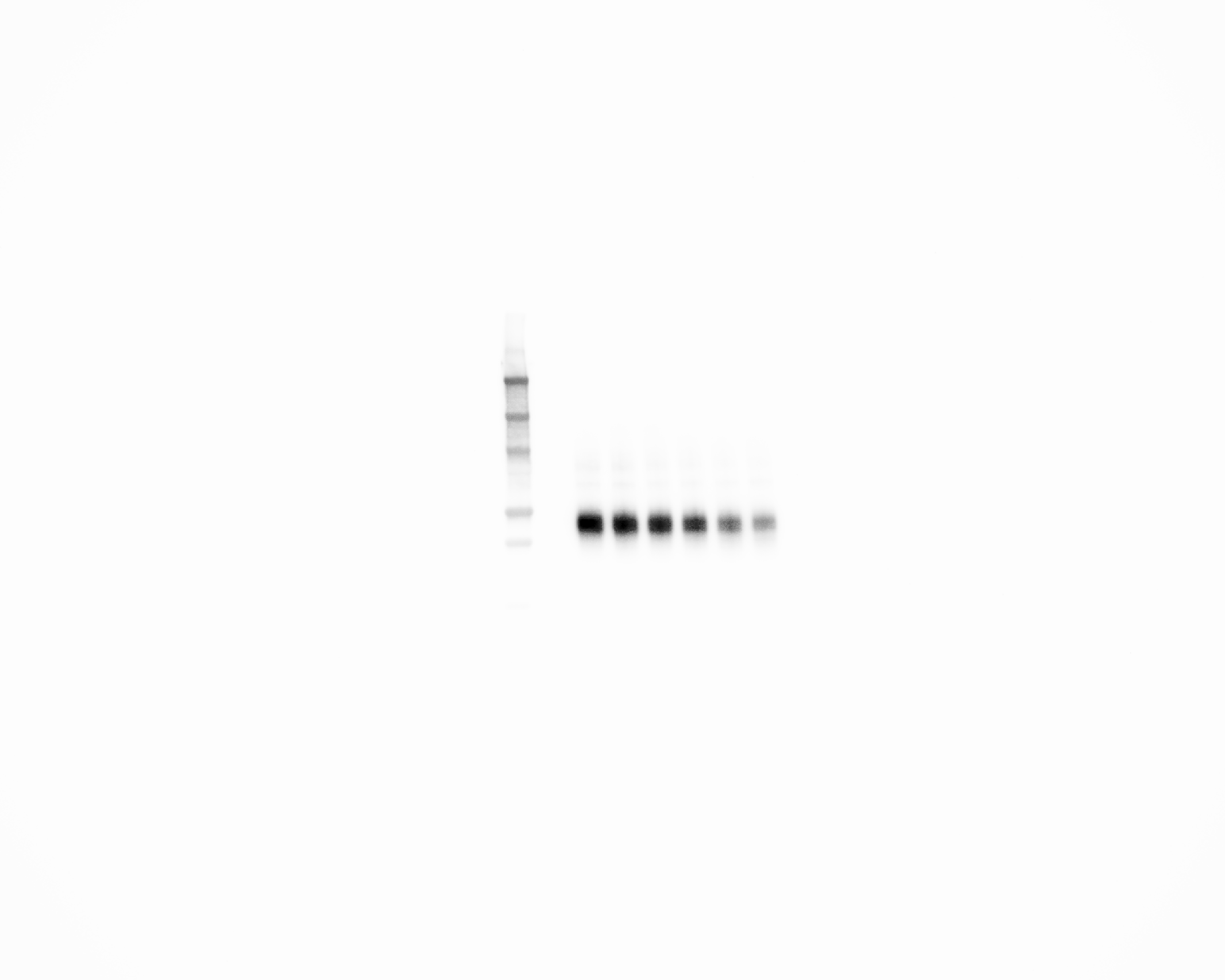

Supplement: Figure 7—source data 1. [file elife-93908-fig7-data1.zip › Figure 7A input, anti-ASGR1 Raw Data.tif]

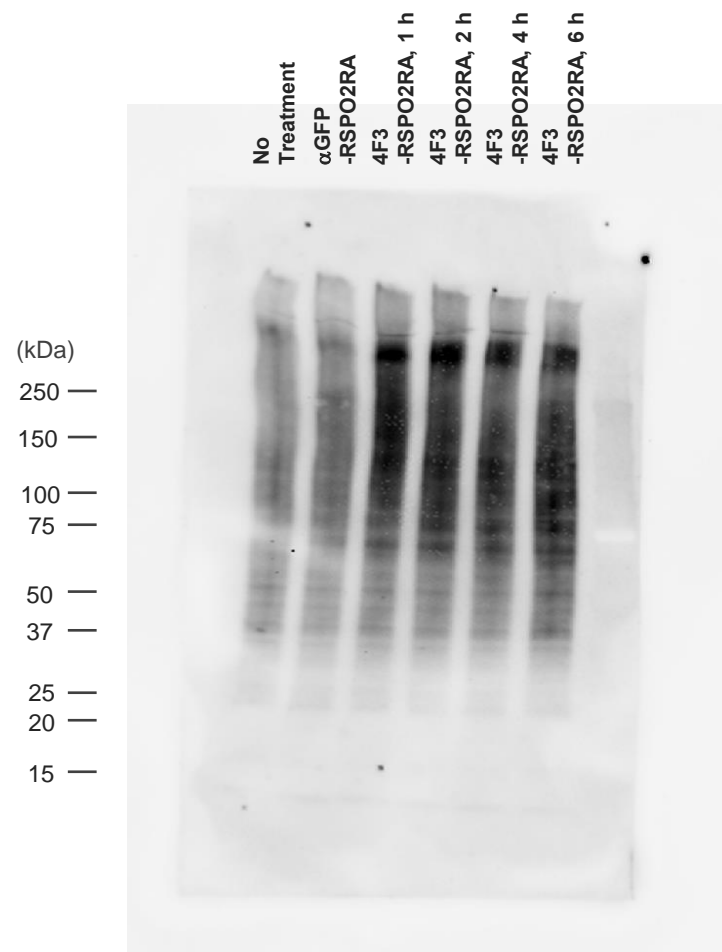

Supplement: Figure 7—source data 1. [file elife-93908-fig7-data1.zip › Figure 7A input, anti-Ubiquitin Laballed Raw Data.pdf]

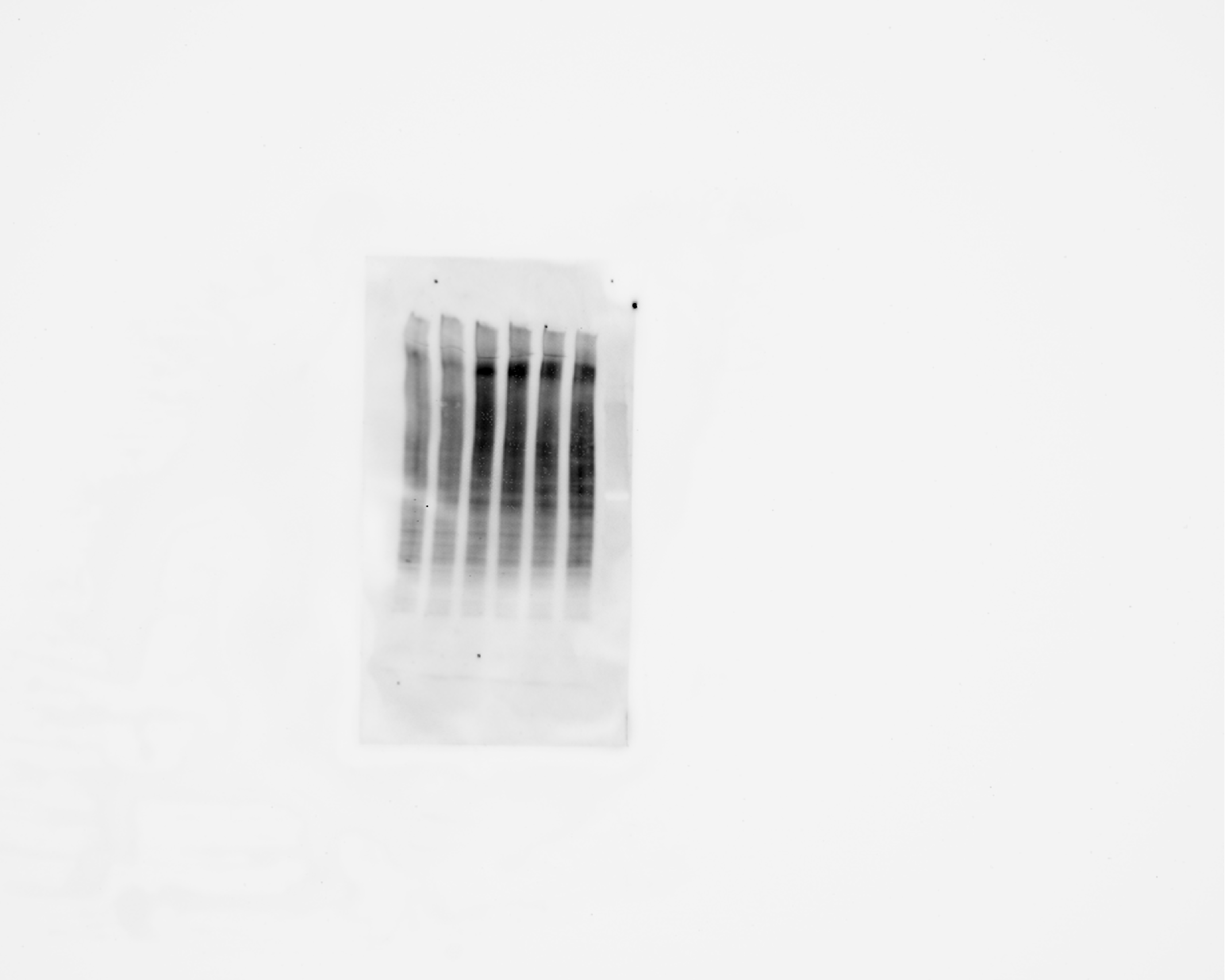

Supplement: Figure 7—source data 1. [file elife-93908-fig7-data1.zip › Figure 7A input, anti-Ubiquitin Raw Data.tif]

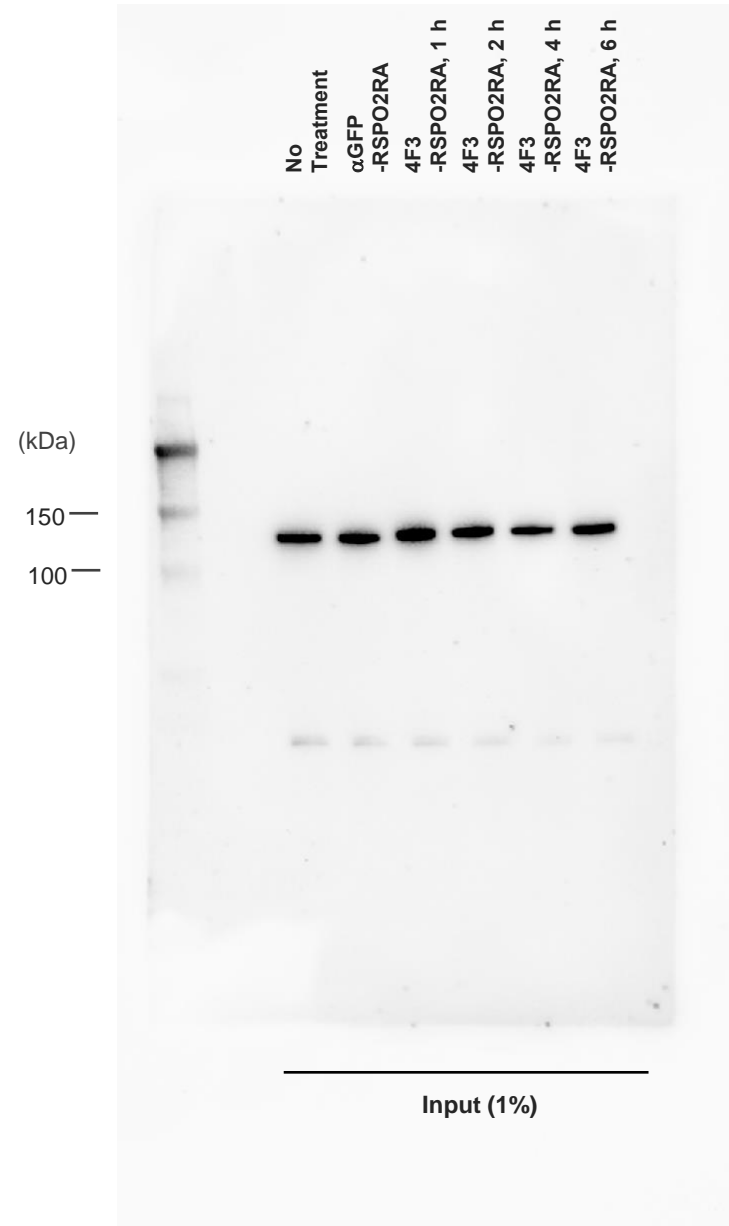

Supplement: Figure 7—source data 1. [file elife-93908-fig7-data1.zip › Figure 7A input, anti-Vinculin Labelled Raw Data.pdf]

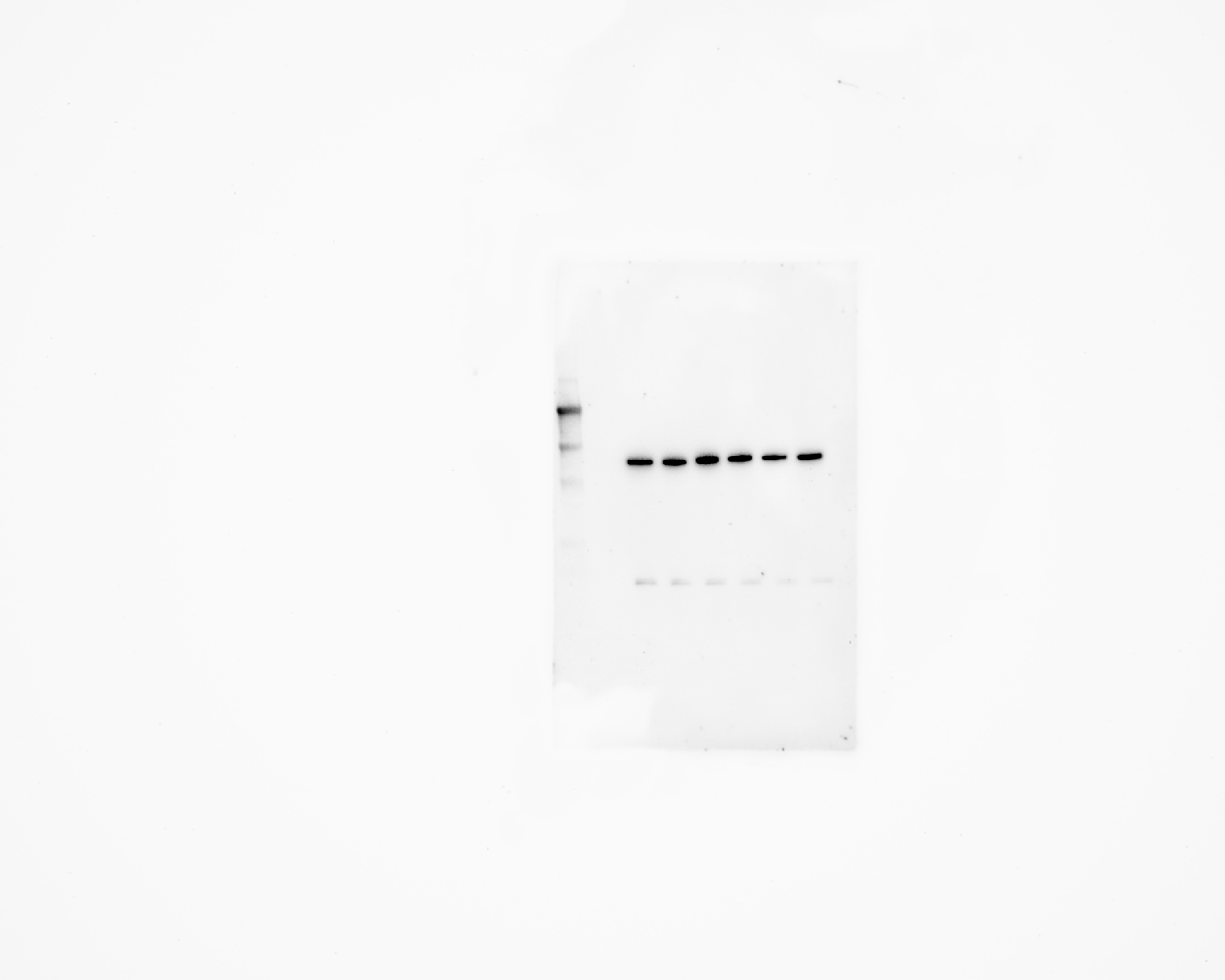

Supplement: Figure 7—source data 1. [file elife-93908-fig7-data1.zip › Figure 7A input, anti-Vinculin Raw Data.tif]

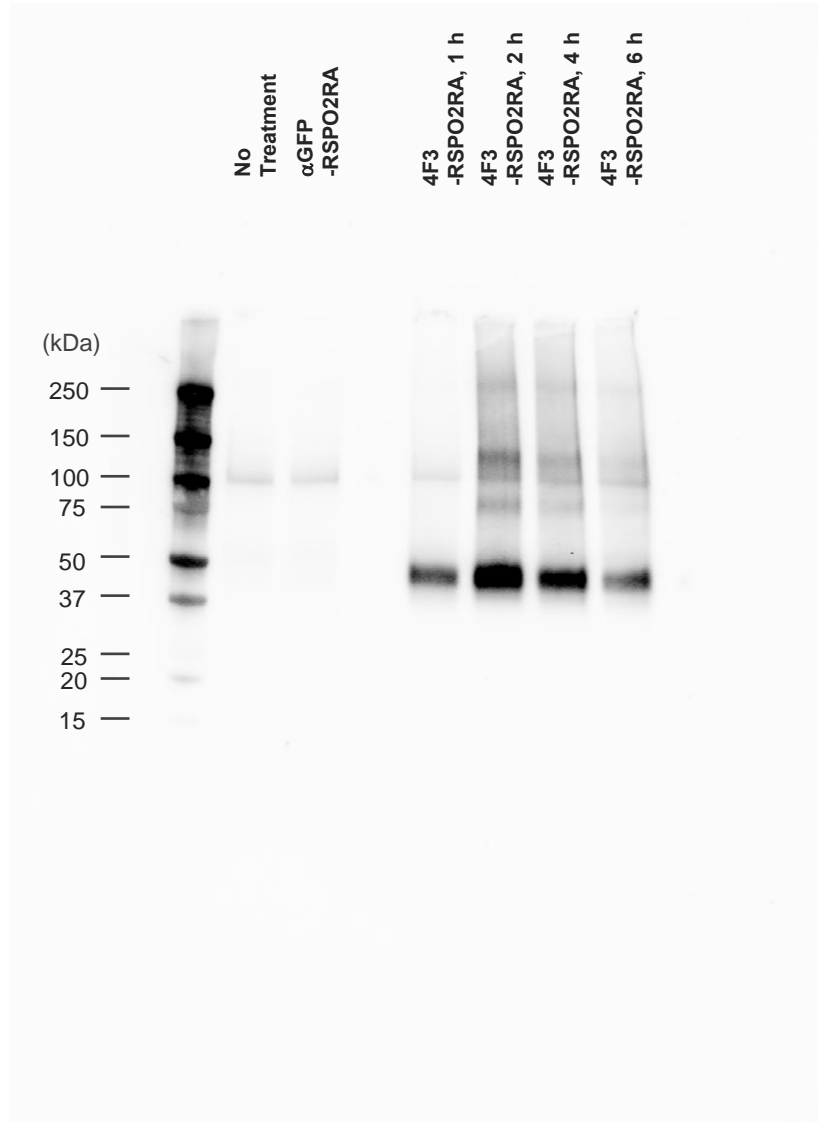

Supplement: Figure 7—source data 1. [file elife-93908-fig7-data1.zip › Figure 7A IP_Ubiquitin, IB_ASGR1 Labelled Raw Data.pdf]

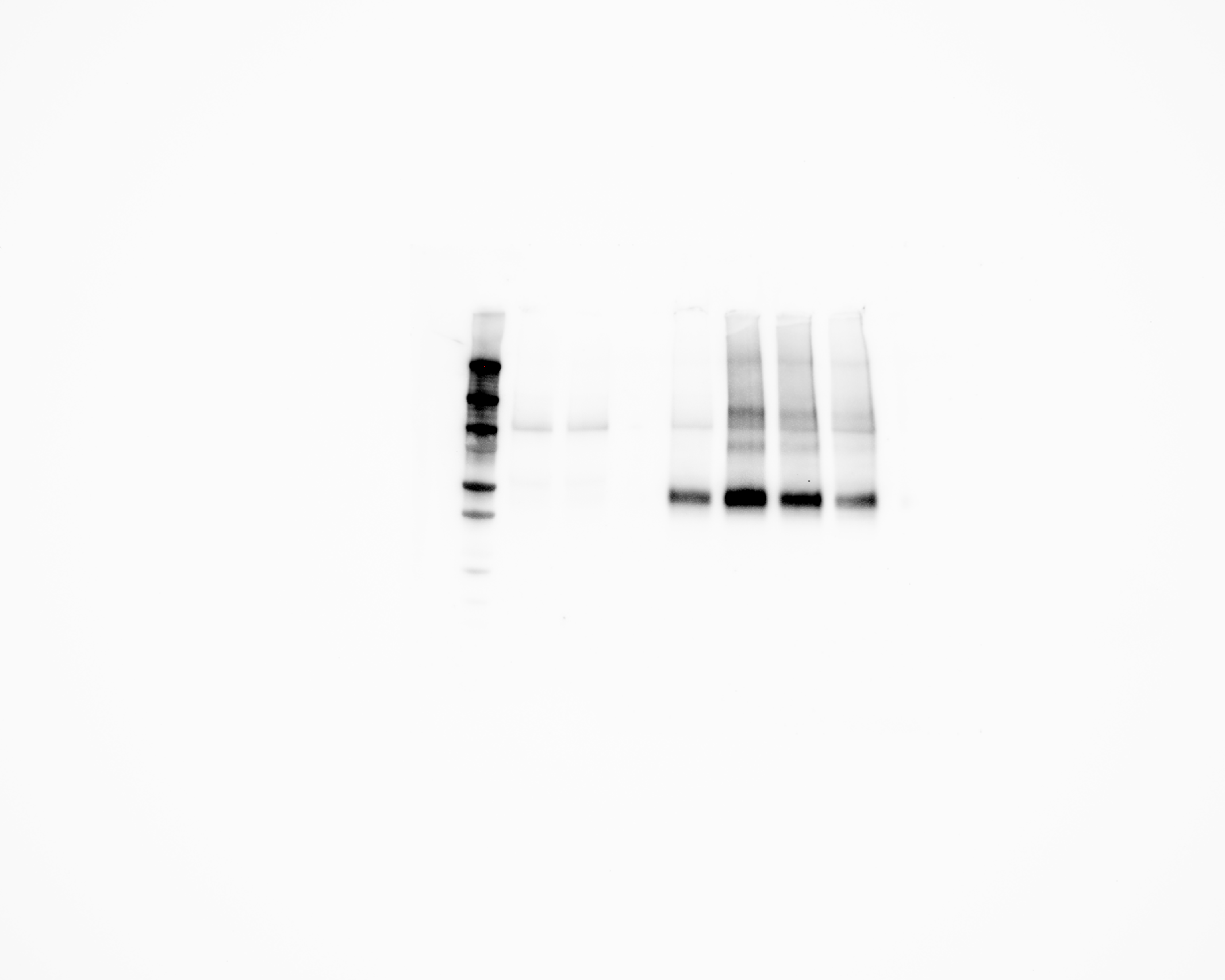

Supplement: Figure 7—source data 1. [file elife-93908-fig7-data1.zip › Figure 7A IP_Ubiquitin, IB_ASGR1 Raw Data.tif]

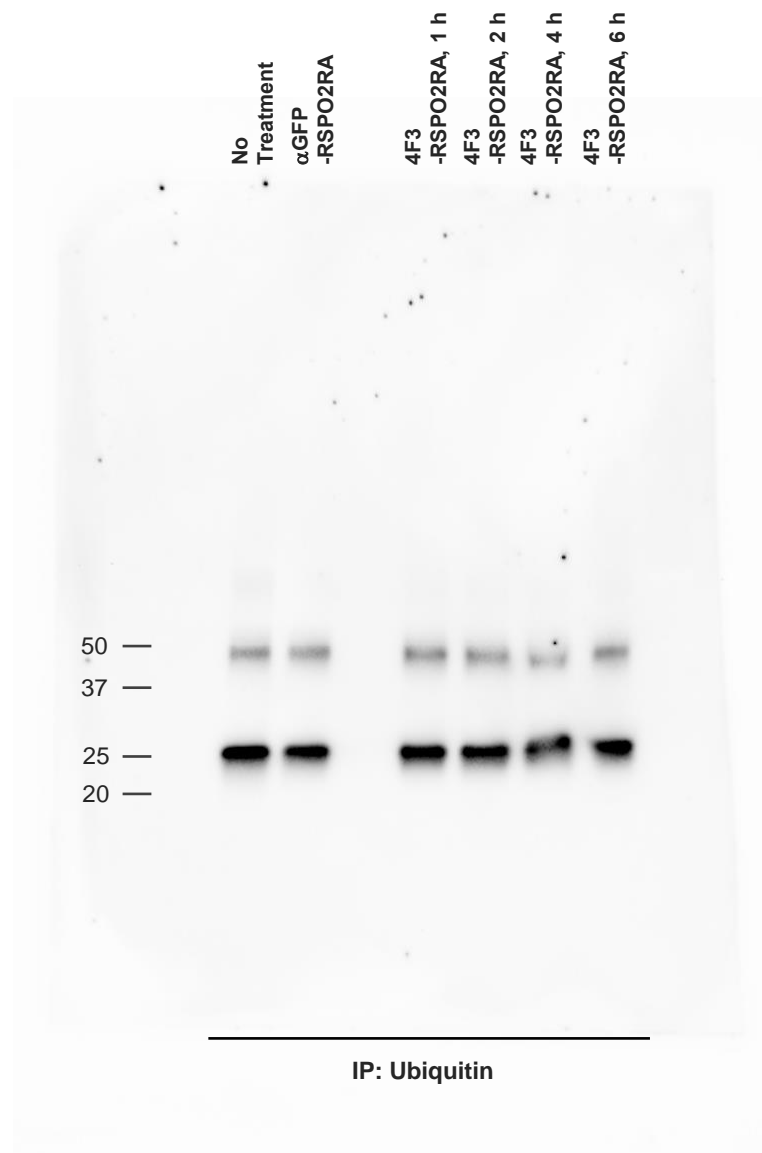

Supplement: Figure 7—source data 1. [file elife-93908-fig7-data1.zip › Figure 7A IP_Ubiquitin, IB_Mouse IgG (H&L) Labelled Raw Data.pdf]

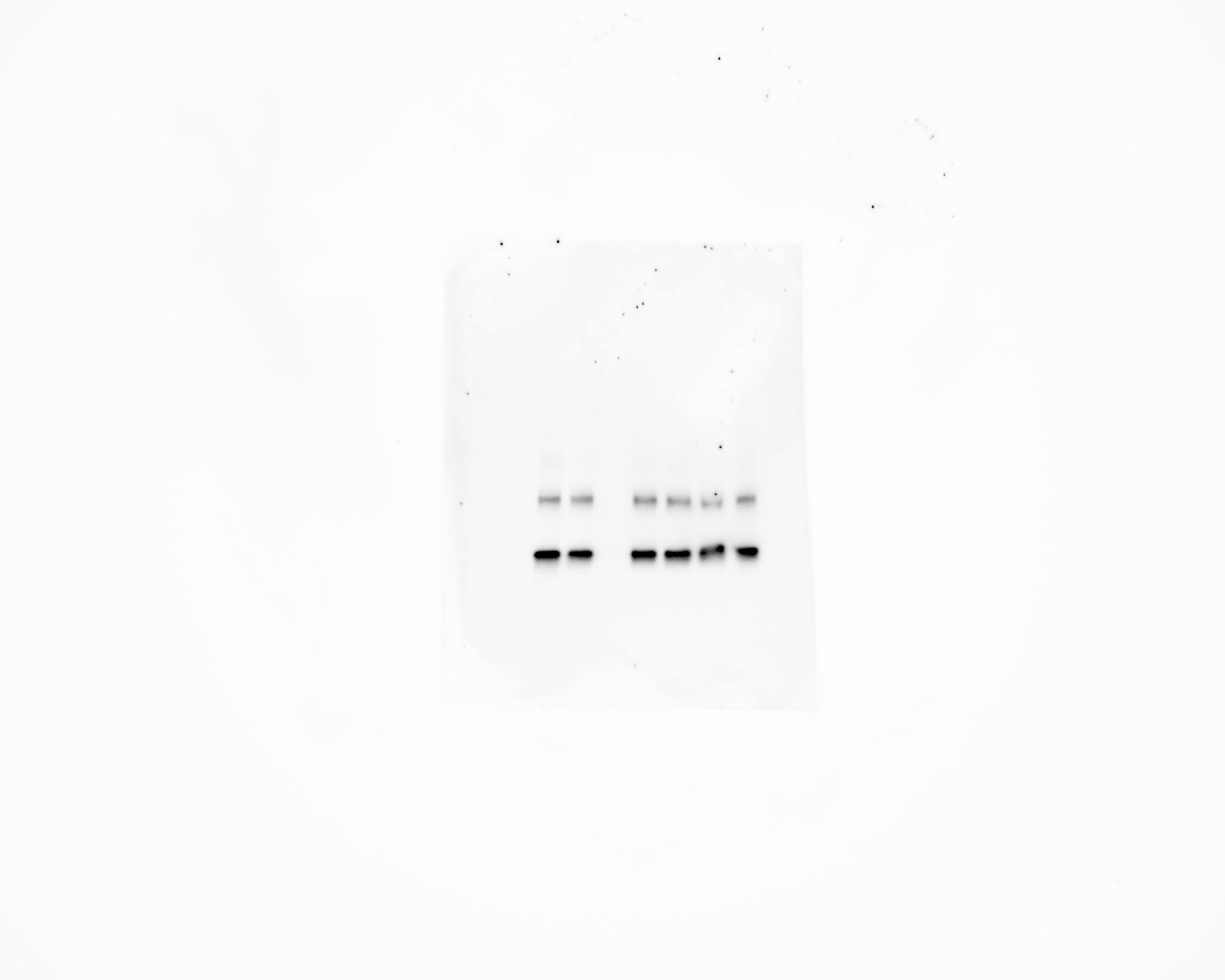

Supplement: Figure 7—source data 1. [file elife-93908-fig7-data1.zip › Figure 7A IP_Ubiquitin, IB_Mouse IgG (H&L) Raw Data.tif]

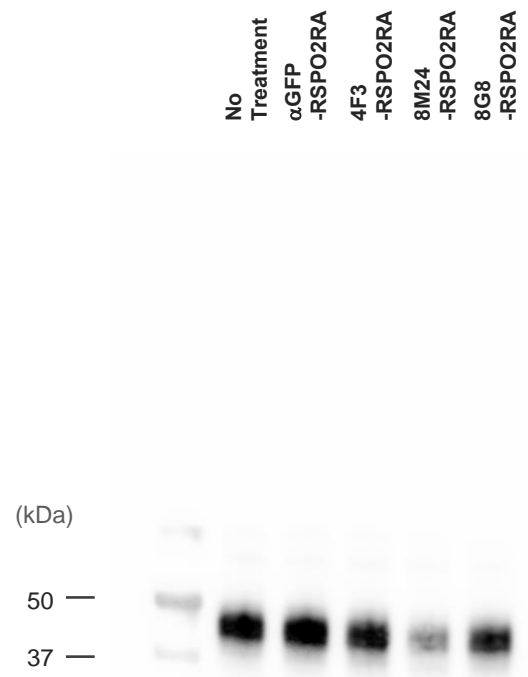

Supplement: Figure 7—source data 2. [file elife-93908-fig7-data2.zip › Figure 7B input, anti-ASGR1 Labelled Raw Data.pdf]

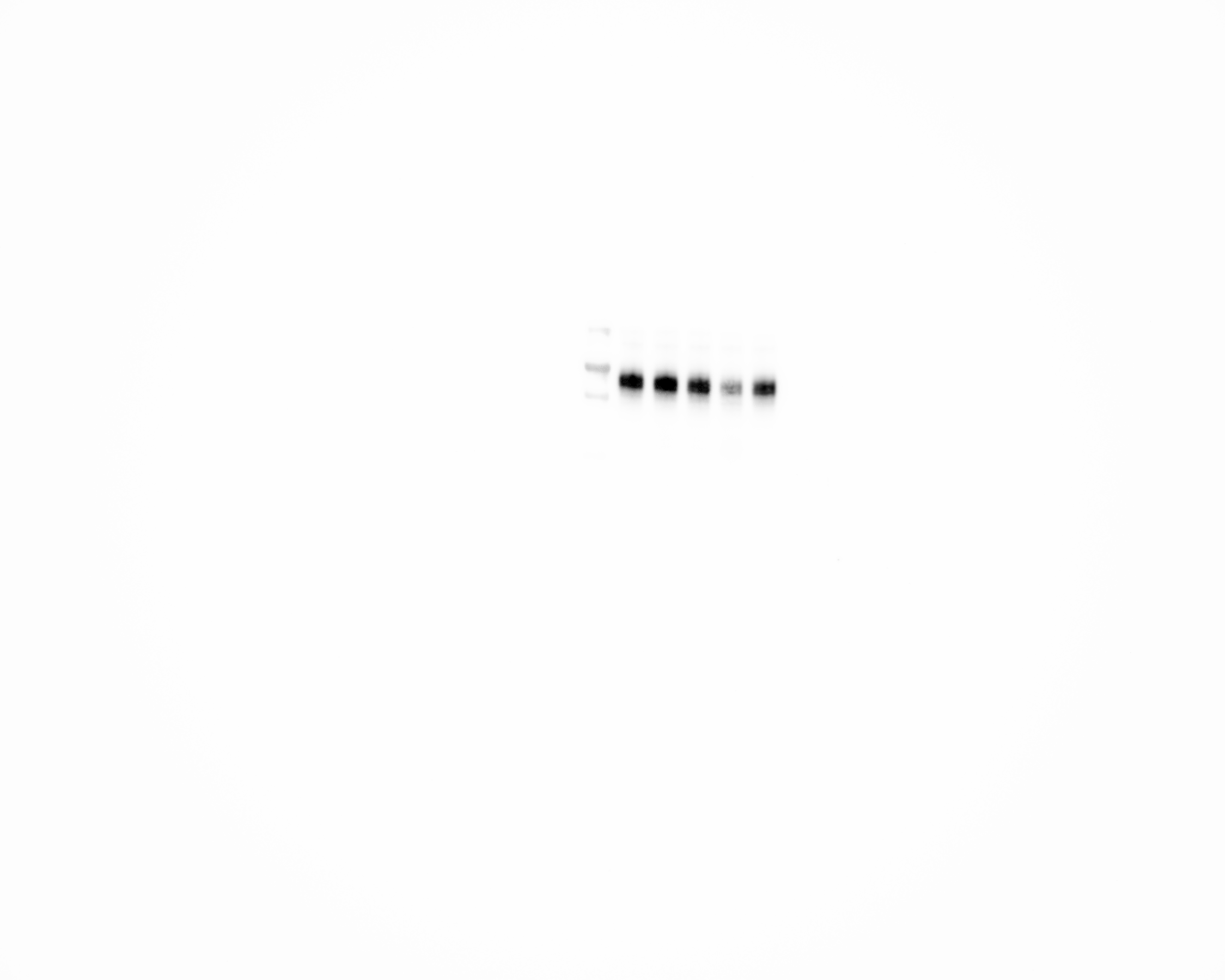

Supplement: Figure 7—source data 2. [file elife-93908-fig7-data2.zip › Figure 7B input, anti-ASGR1 Raw Data.tif]

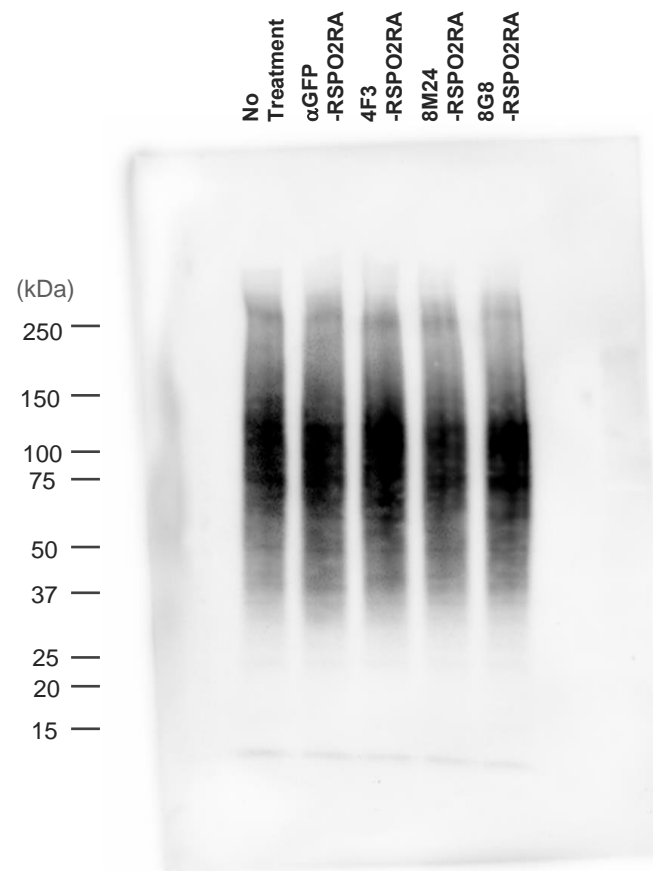

Supplement: Figure 7—source data 2. [file elife-93908-fig7-data2.zip › Figure 7B input, anti-Ubiquitin Laballed Raw Data.pdf]

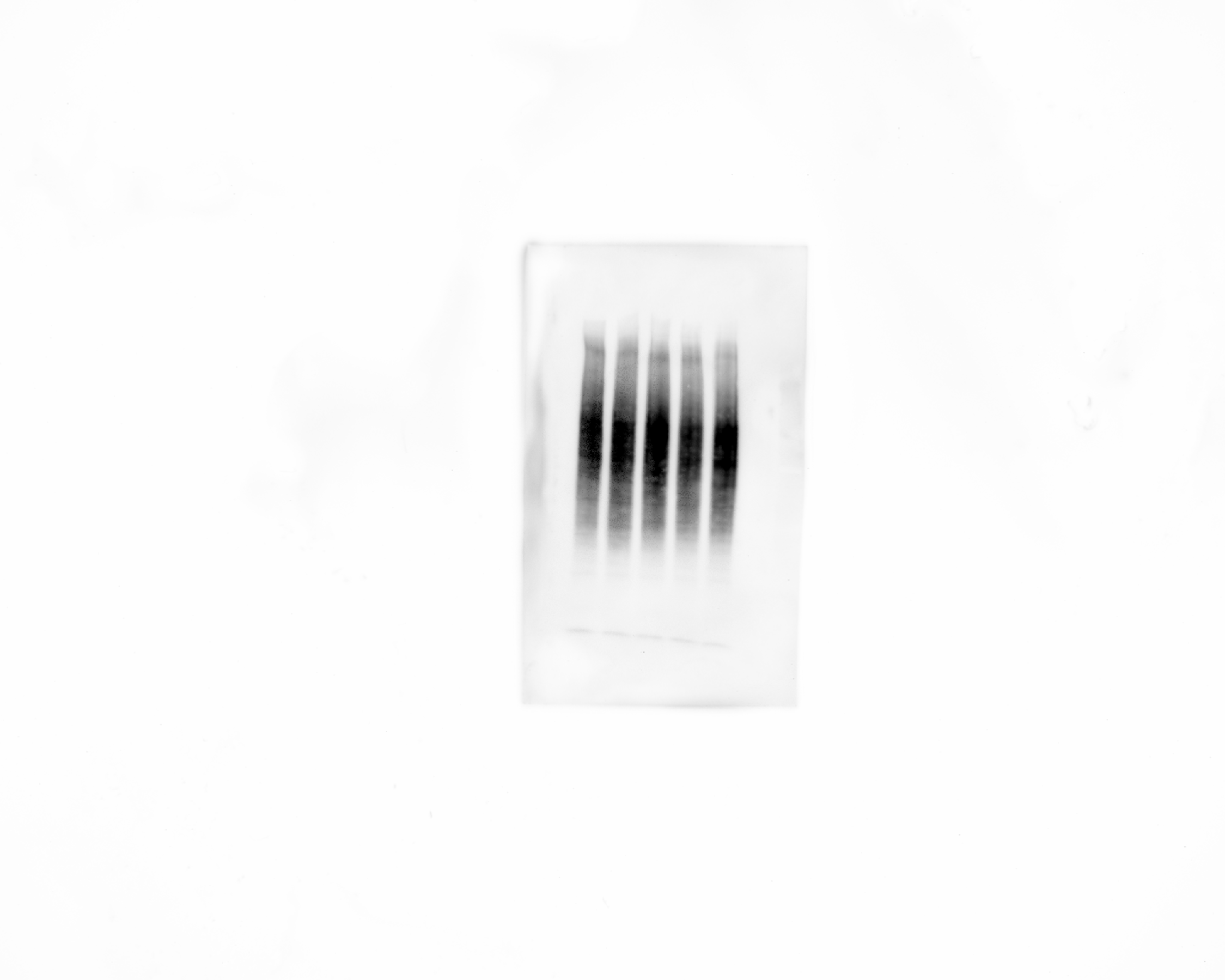

Supplement: Figure 7—source data 2. [file elife-93908-fig7-data2.zip › Figure 7B input, anti-Ubiquitin Raw Data.tif]

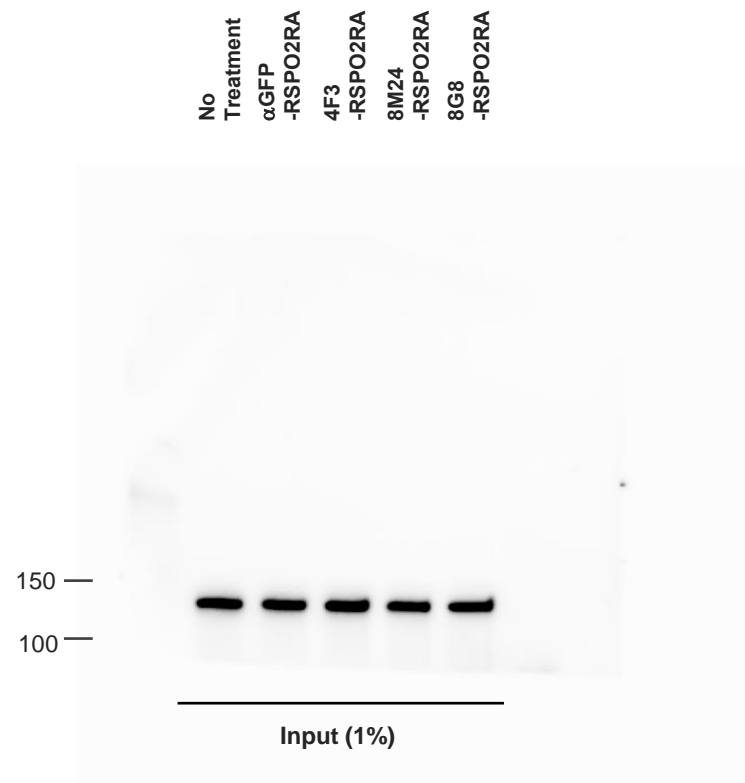

Supplement: Figure 7—source data 2. [file elife-93908-fig7-data2.zip › Figure 7B input, anti-Vinculin Labelled Raw Data.pdf]

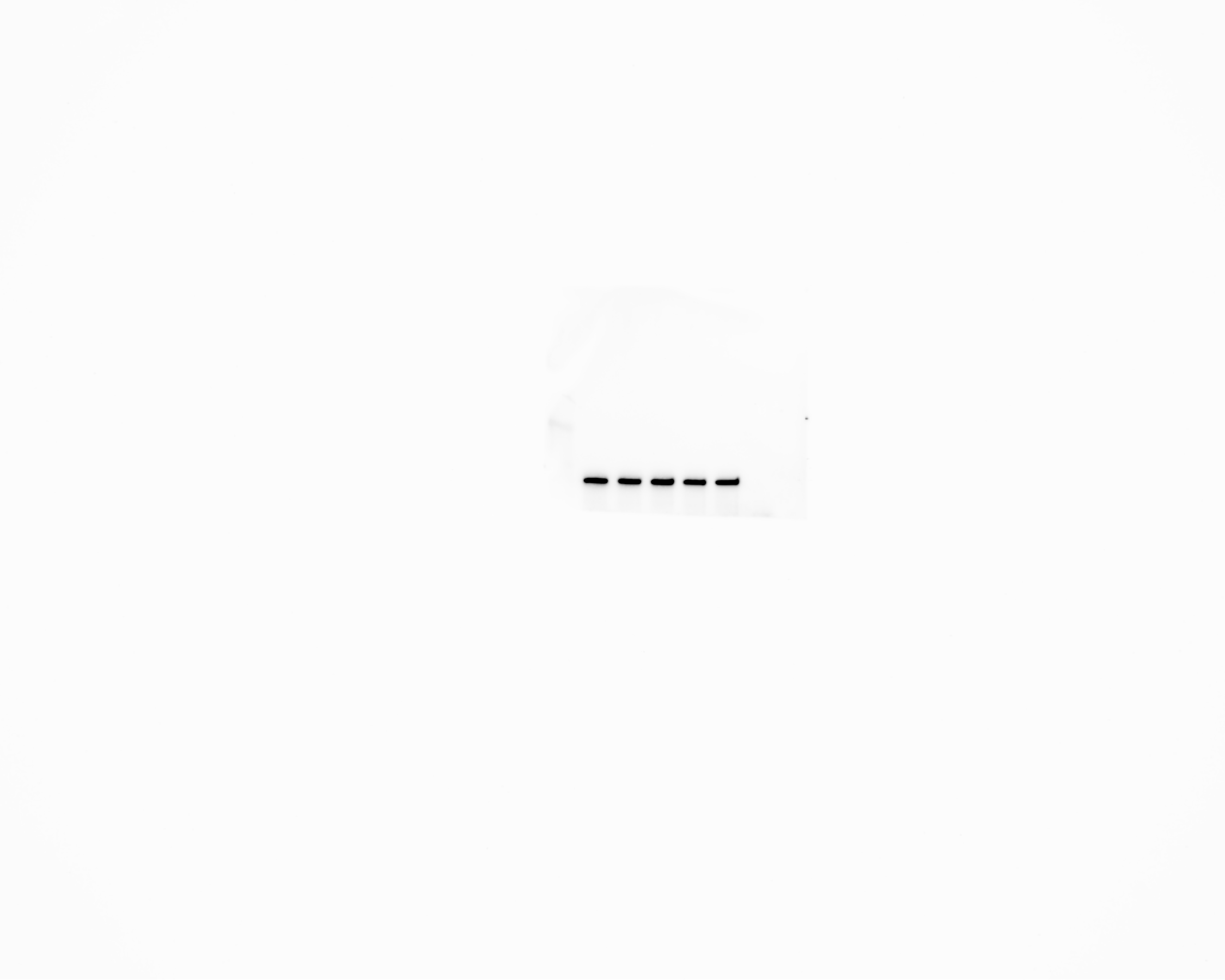

Supplement: Figure 7—source data 2. [file elife-93908-fig7-data2.zip › Figure 7B input, anti-Vinculin Raw Data.tif]

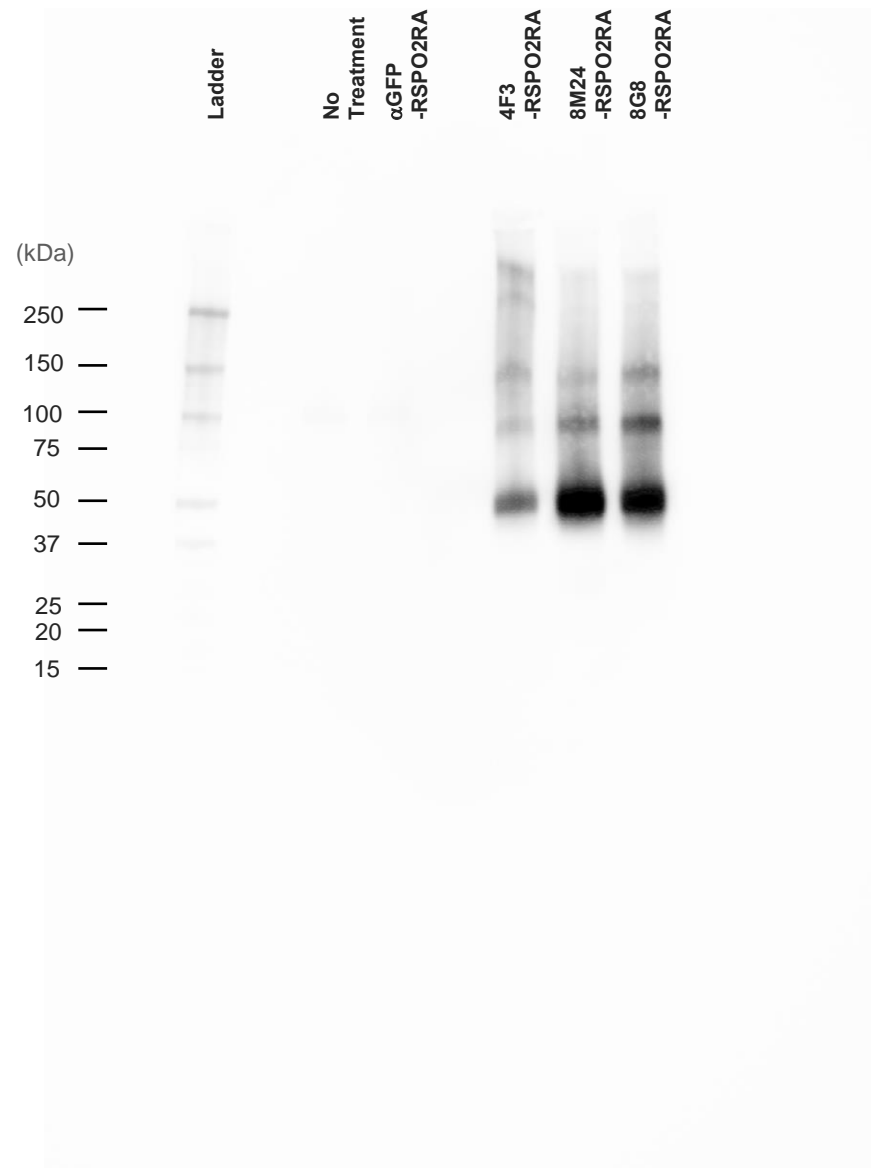

Supplement: Figure 7—source data 2. [file elife-93908-fig7-data2.zip › Figure 7B IP_Ubiquitin, IB_ASGR1 Labelled Raw Data.pdf]

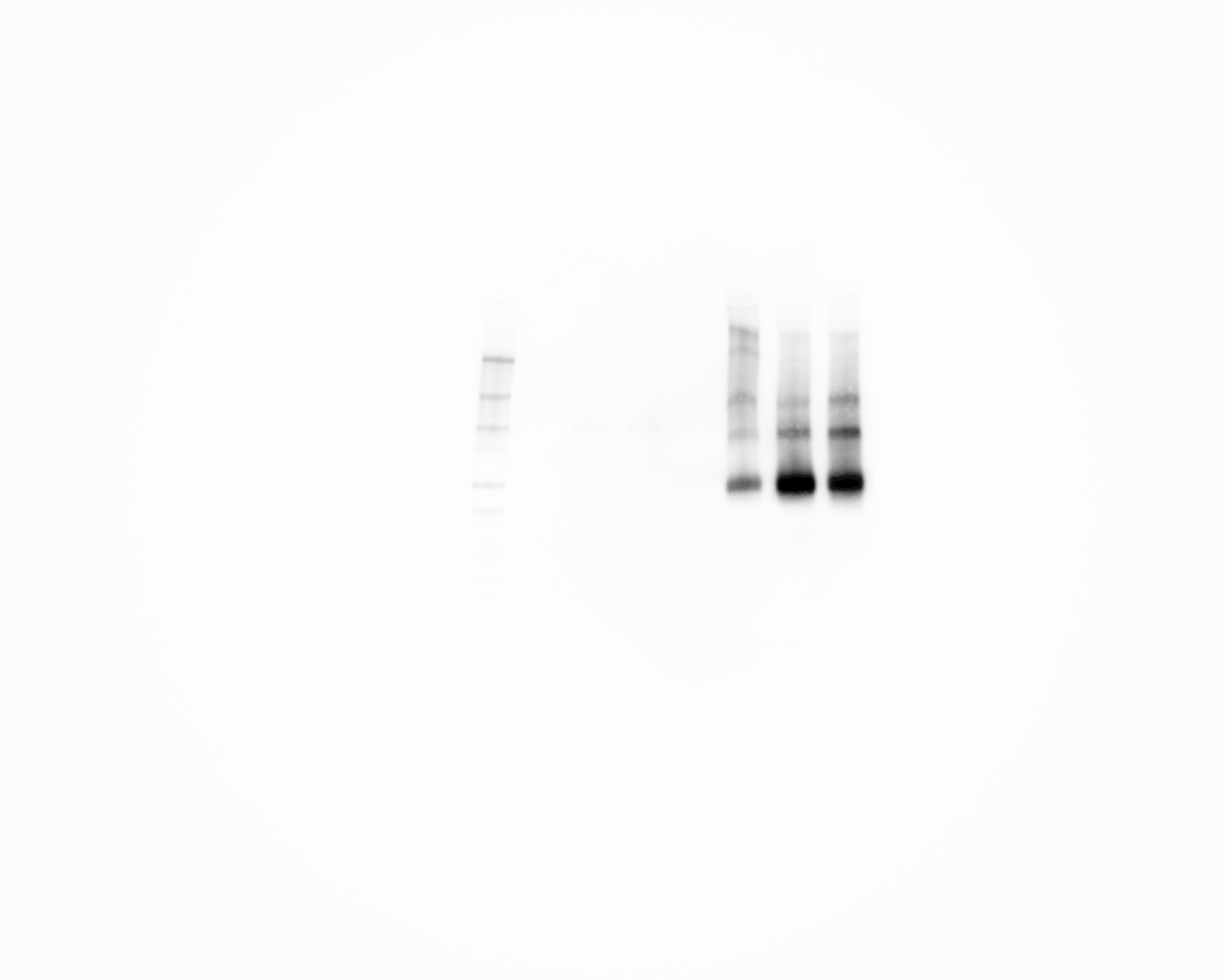

Supplement: Figure 7—source data 2. [file elife-93908-fig7-data2.zip › Figure 7B IP_Ubiquitin, IB_ASGR1 Raw Data.tif]

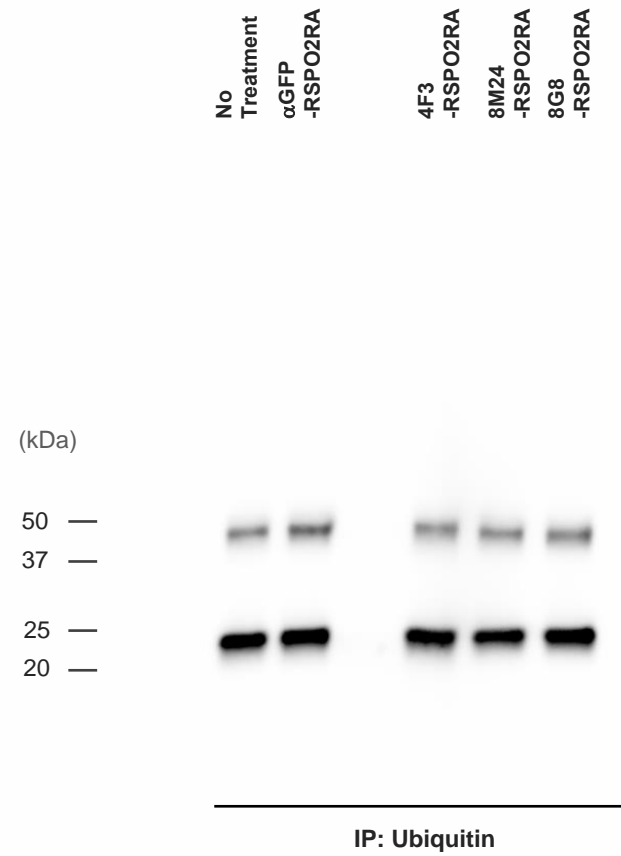

Supplement: Figure 7—source data 2. [file elife-93908-fig7-data2.zip › Figure 7B IP_Ubiquitin, IB_Mouse IgG (H&L) Labelled Raw Data.pdf]

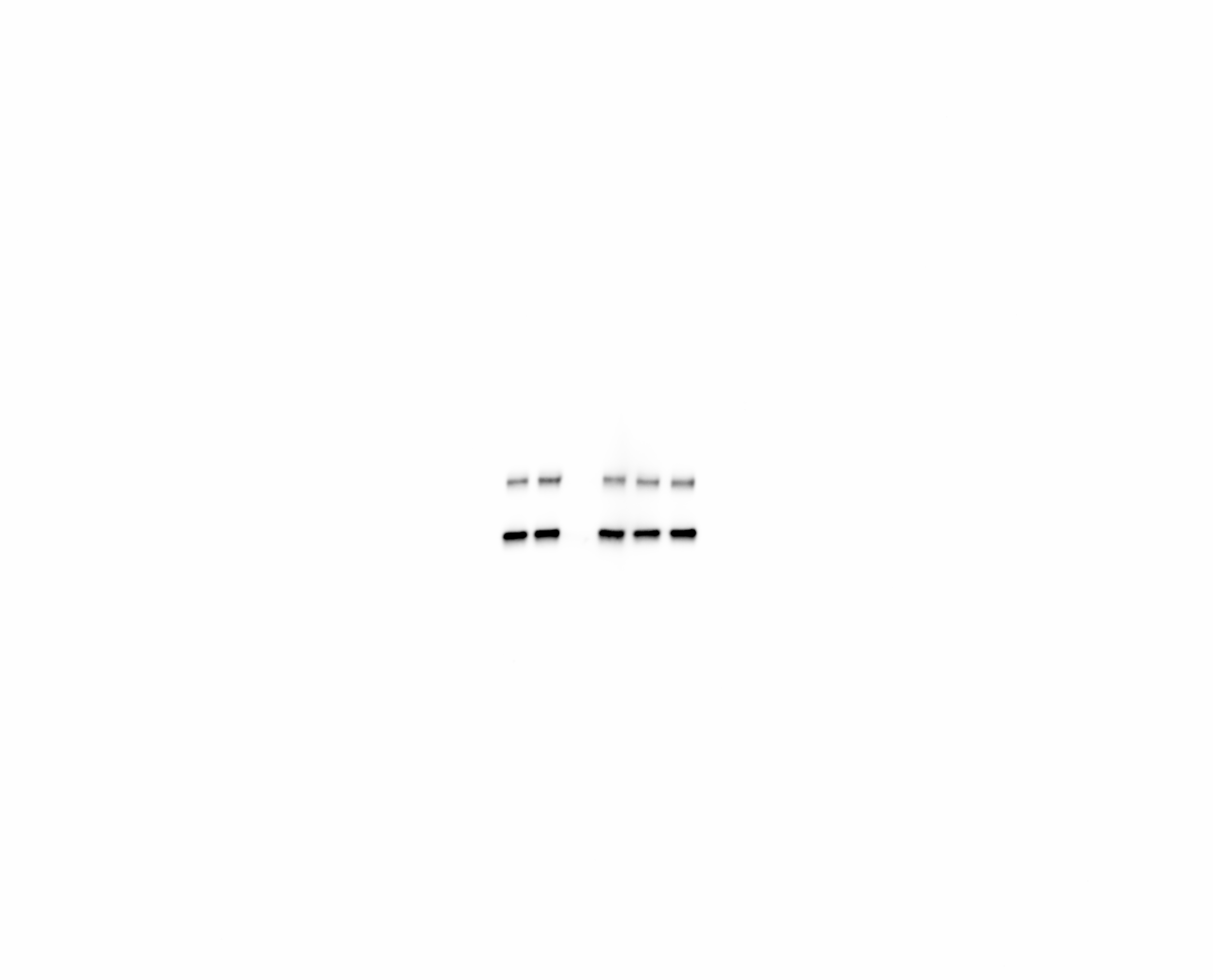

Supplement: Figure 7—source data 2. [file elife-93908-fig7-data2.zip › Figure 7B IP_Ubiquitin, IB_Mouse IgG (H&L) Raw Data.tif]

mut ASGR1

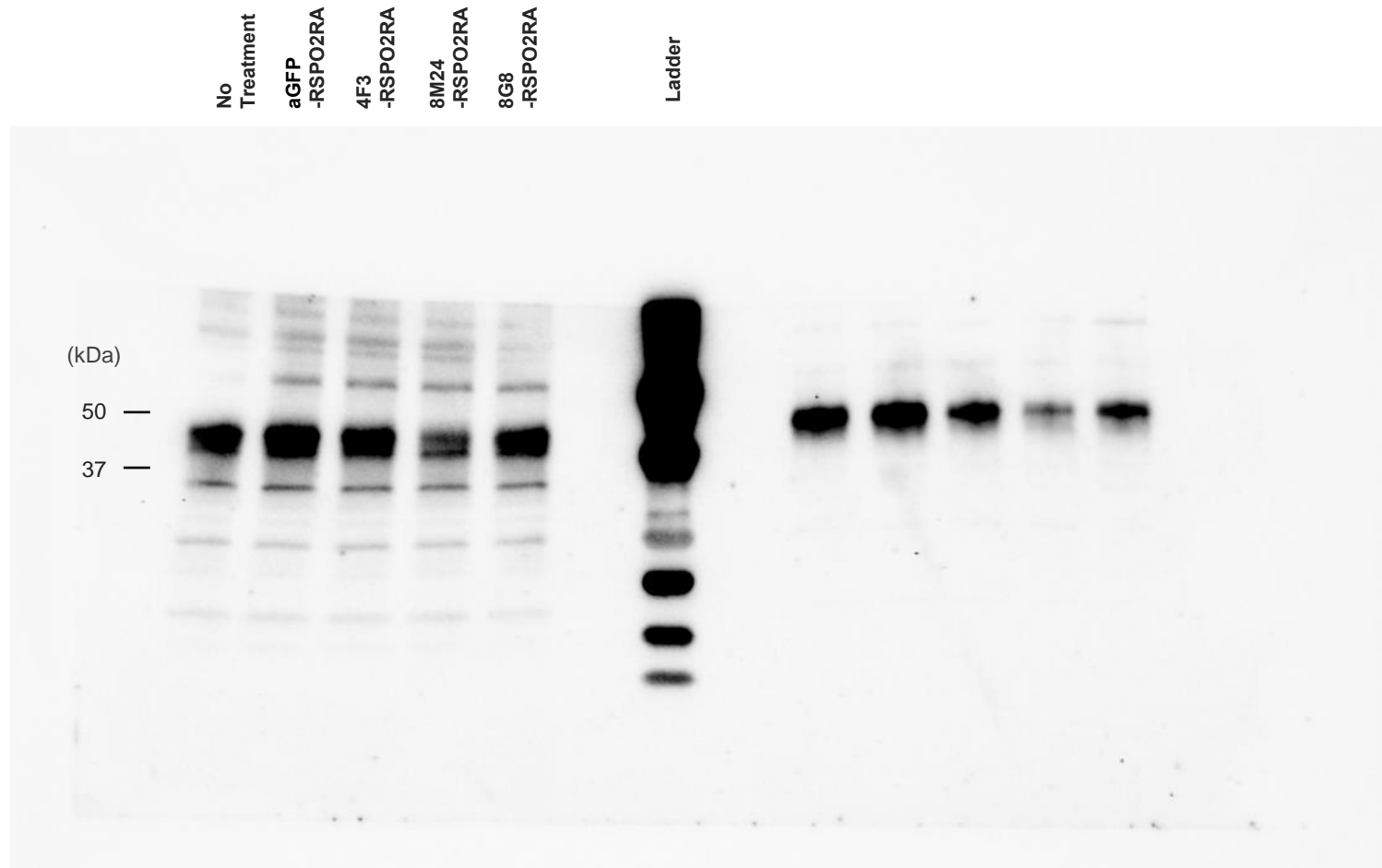

Supplement: Figure 7—source data 3. [file elife-93908-fig7-data3.zip › Figure 7C anti-ASGR1 with mut ASGR1 transfection_Labelled Raw Data.pdf]

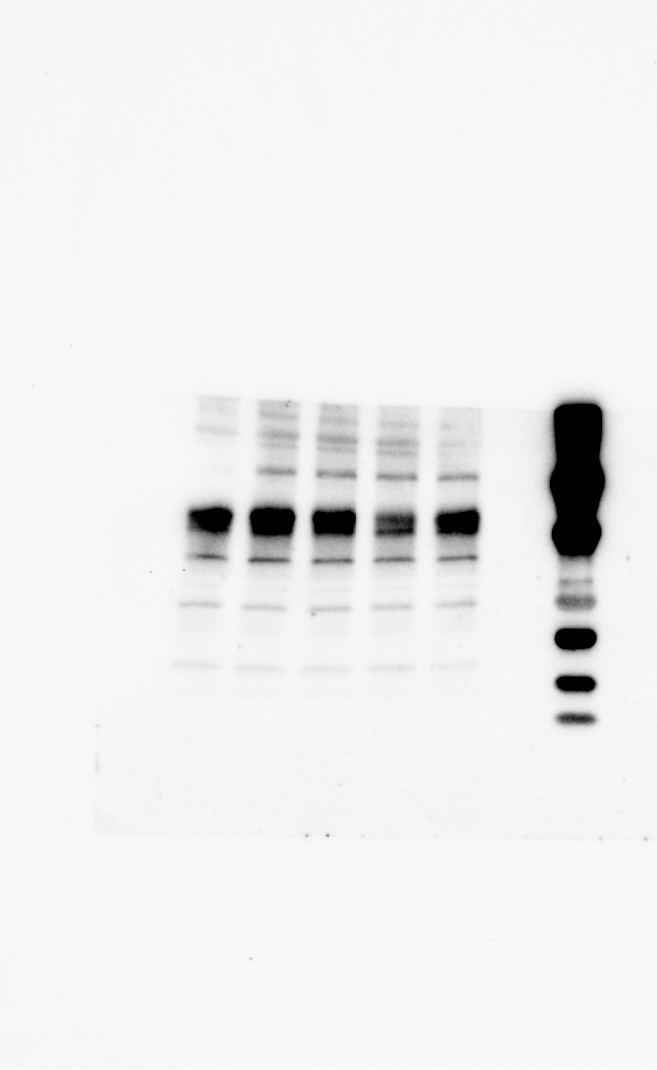

Supplement: Figure 7—source data 3. [file elife-93908-fig7-data3.zip › Figure 7C anti-ASGR1 with mut ASGR1 transfection_Raw Data.tif]

WT ASGR1

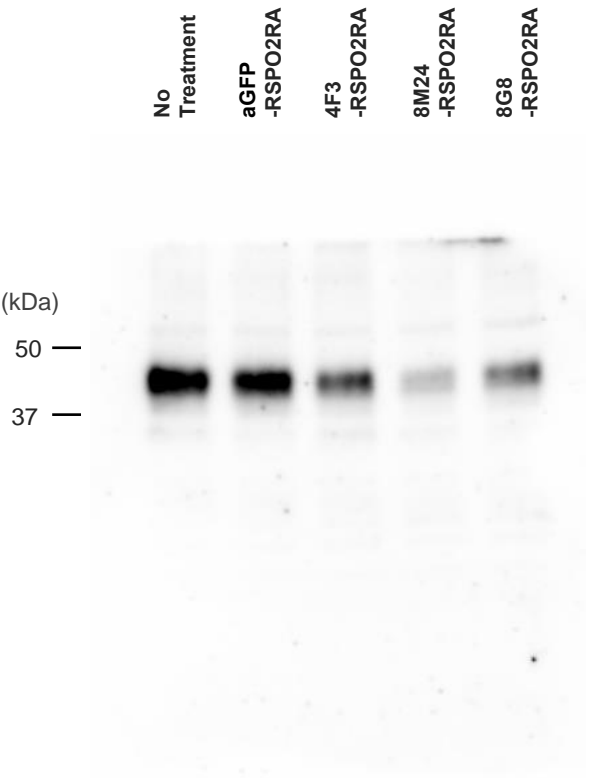

Supplement: Figure 7—source data 3. [file elife-93908-fig7-data3.zip › Figure 7C anti-ASGR1 with WT ASGR1 transfection_Labelled Raw Data.pdf]

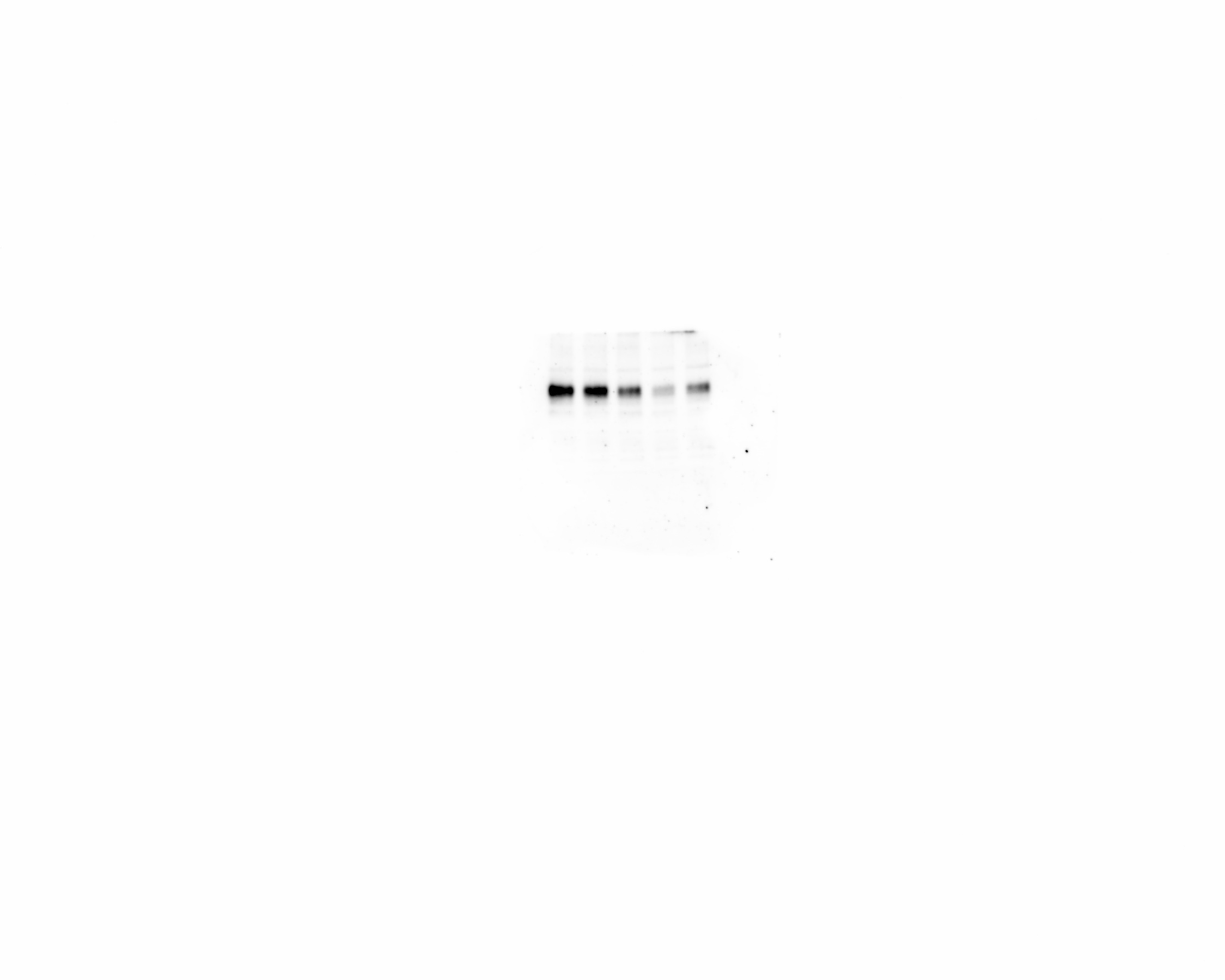

Supplement: Figure 7—source data 3. [file elife-93908-fig7-data3.zip › Figure 7C anti-ASGR1 with WT ASGR1 transfection_Raw Data.tif]

mut ASGR1

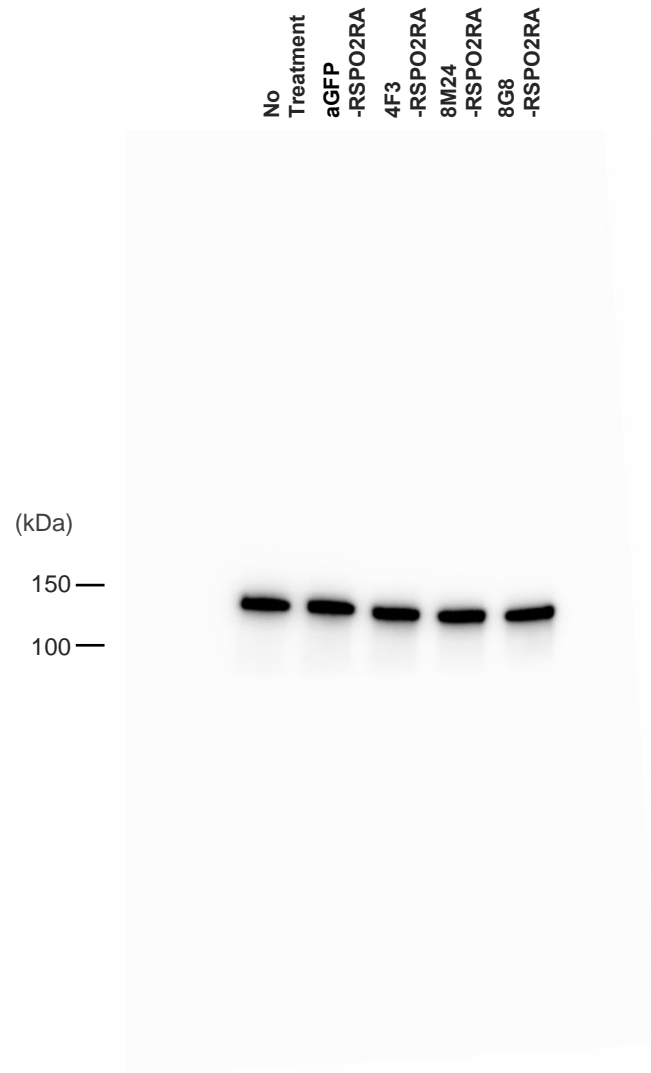

Supplement: Figure 7—source data 3. [file elife-93908-fig7-data3.zip › Figure 7C anti-Vinculin with mut ASGR1 transfection_Labelled Raw Data.pdf]

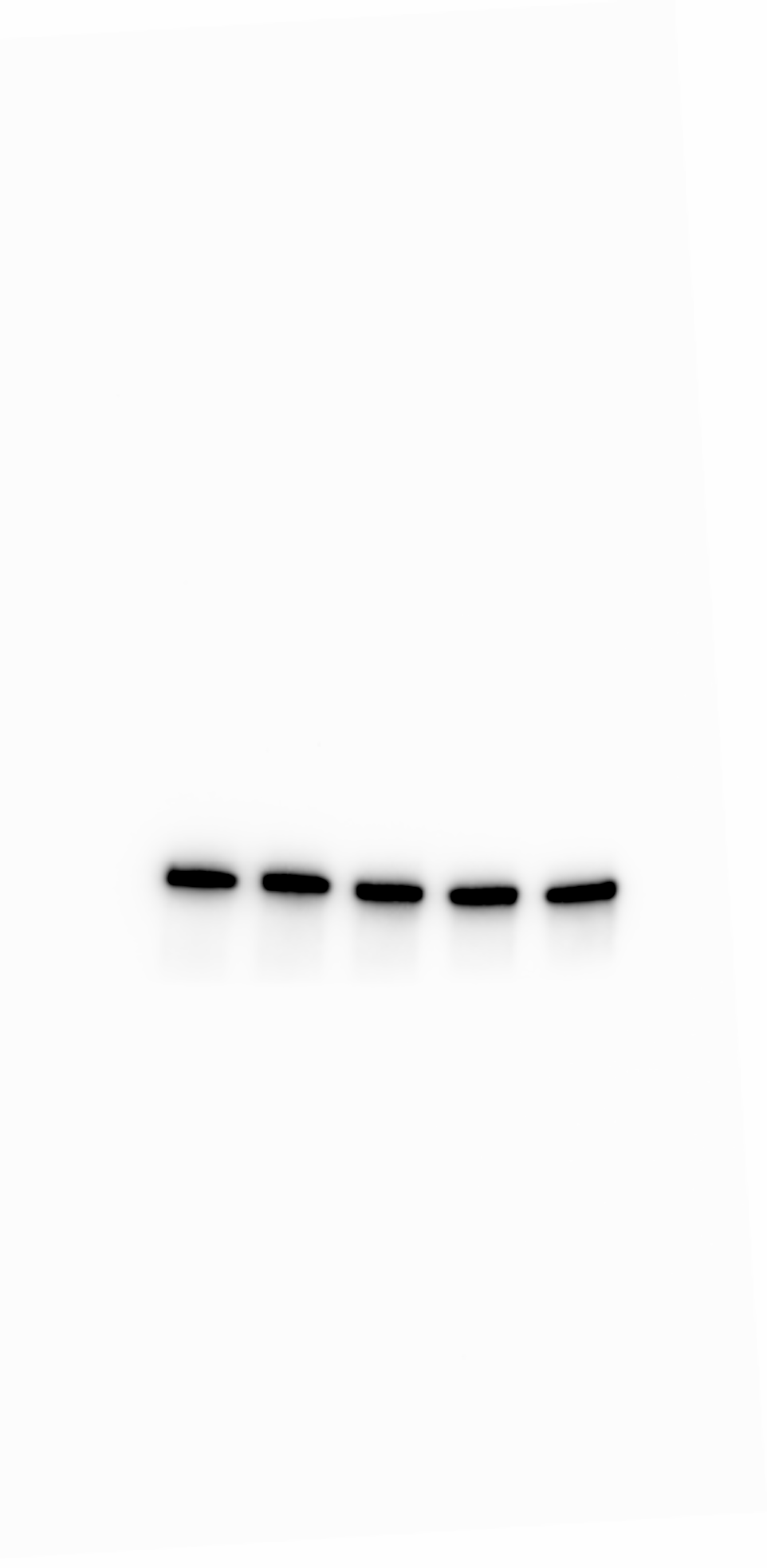

Supplement: Figure 7—source data 3. [file elife-93908-fig7-data3.zip › Figure 7C anti-Vinculin with mut ASGR1 transfection_Raw Data.tif]

# WT ASGR1

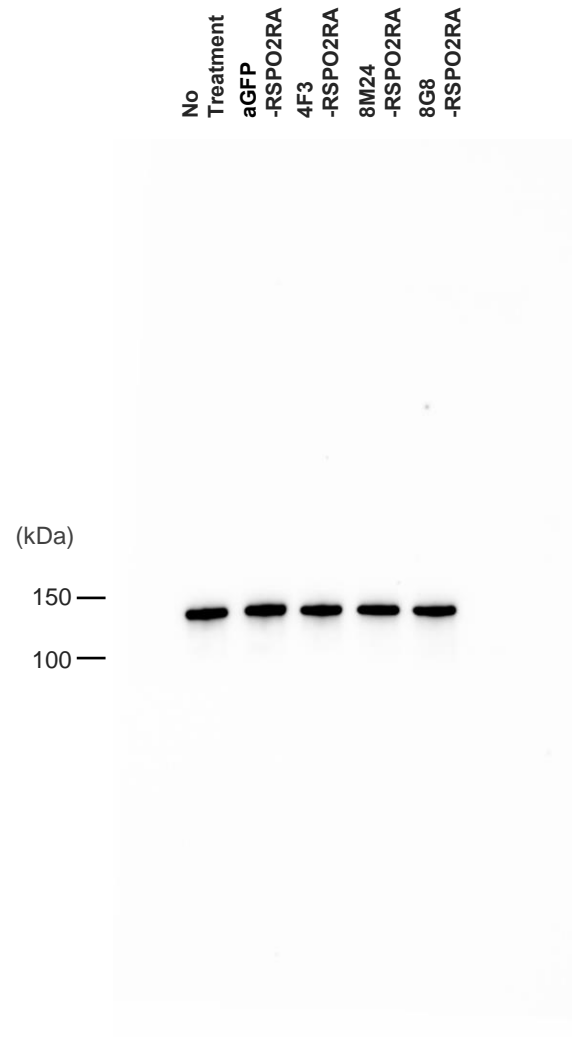

Supplement: Figure 7—source data 3. [file elife-93908-fig7-data3.zip › Figure 7C anti-Vinculin with WT ASGR1 transfection_Labelled Raw Data.pdf]

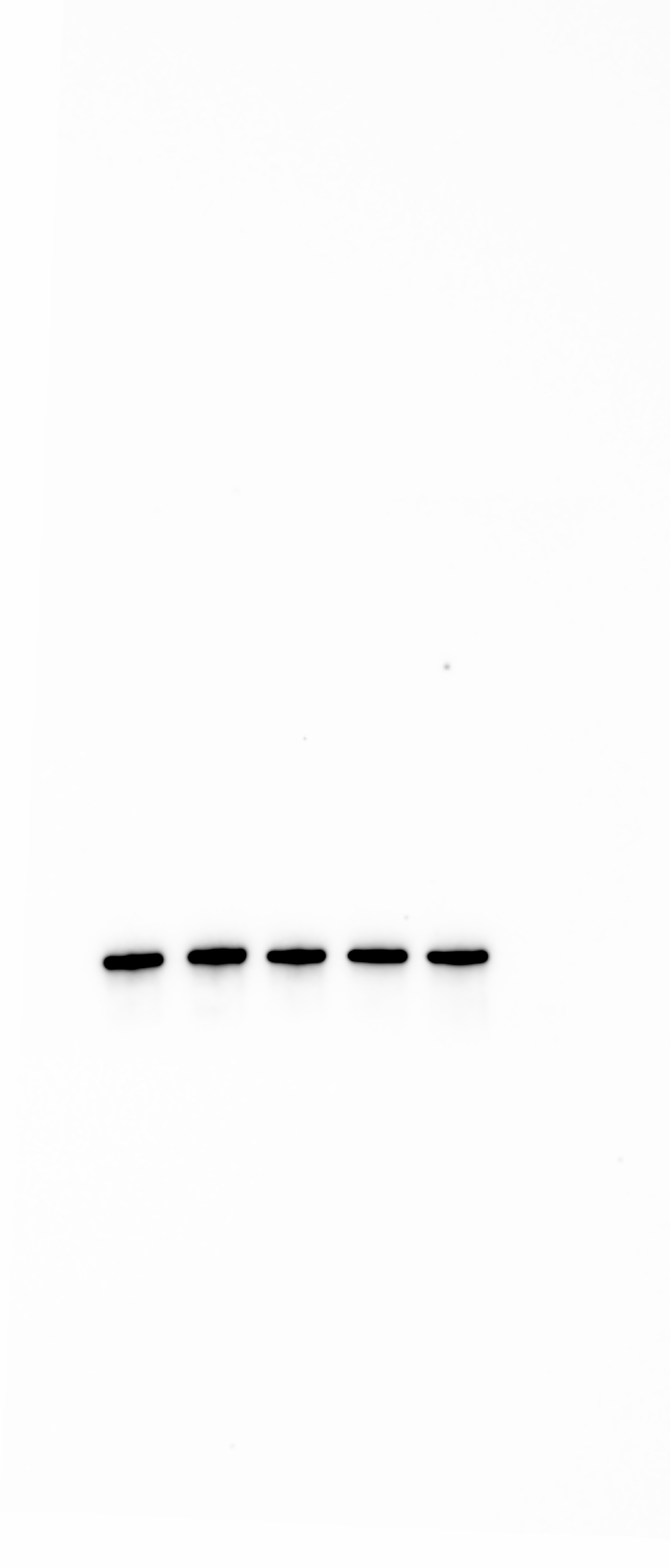

Supplement: Figure 7—source data 3. [file elife-93908-fig7-data3.zip › Figure 7C anti-Vinculin with WT ASGR1 transfection_Raw Data.tif]
